# Multifunctional evolution of B and *AGL6* MADS box genes in orchids

**DOI:** 10.1038/s41467-021-21229-w

**Published:** 2021-02-10

**Authors:** Hsing-Fun Hsu, Wei-Han Chen, Yi-Hsuan Shen, Wei-Han Hsu, Wan-Ting Mao, Chang-Hsien Yang

**Affiliations:** 1 Institute of Biotechnology, National Chung Hsing University, Taichung, Taiwan 40227 ROC; 2 Advanced Plant Biotechnology Center, National Chung Hsing University, Taichung, Taiwan 40227 ROC

**Keywords:** Gene expression, Plant morphogenesis, Flowering

## Abstract

We previously found that B and AGL6 proteins form L (OAP3-2/OAGL6-2/OPI) and SP (OAP3-1/OAGL6-1/OPI) complexes to determine lip/sepal/petal identities in orchids. Here, we show that the functional L’ (OAP3-1/OAGL6-2/OPI) and SP’ (OAP3-2/OAGL6-1/OPI) complexes likely exist and *AP3*/*PI/AGL6* genes have acquired additional functions during evolution. We demonstrate that the presumed L’ complex changes the structure of the lower lateral sepals and helps the lips fit properly in the center of the flower. In addition, we find that OAP3-1/OAGL6-1/OPI in SP along with presumed SP’ complexes regulate anthocyanin accumulation and pigmentation, whereas presumed L’ along with OAP3-2/OAGL6-2/OPI in L complexes promotes red spot formation in the perianth. Furthermore, the B functional proteins OAP3-1/OPI and OAGL6-1 in the SP complex could function separately to suppress sepal/petal senescence and promote pedicel abscission, respectively. These findings expand the current knowledge behind the multifunctional evolution of the B and *AGL6* genes in plants.

## Abstract (web summary)

B class AP3/PI and AGL6-like MADS proteins determine lips and sepals/petals identities in orchids. Here, the authors characterize the extended function of OAP3/OPI/OAGL6 in regulating the specific structure of the lateral sepals, pigmentation/senescence of the perianth and abscission of the pedicel.

## Introduction

In flowering plants, the four whorls of flower organs are regulated by the interaction of four classes of homeotic genes based on the ABCE model^1–5^. In angiosperms, B class proteins consist of two clades, APETALA3 (AP3)- and PISTILLATA (PI)-like proteins, which have been mainly found to control petal and stamen identity^6–9^. AP3/PI heterodimers could further interact with A/E- and C/E-function proteins to form higher-order heterotetrameric complexes in regulating petal and stamen development^6,10–13^. In addition to regulate floral organ identity1^6,14–16^, A/E-class *AGL6* gene has been found to control meristem identity and ovule development^14,15,17–19^. However, whether *AP3*/*PI/AGL6* have evolved additional functions in different plant species during evolution remains unknown.

Based on the conserved principle of the perianth (P) code in orchids, the higher-order SP complex (OAP3-1/OAGL6-1/OAGL6-1/OPI) specifies sepal/petal identity, whereas the L complex (OAP3-2/OAGL6-2/OAGL6-2/OPI) is exclusively required for lip formation^6^. Since the L complex gene *OAGL6-2* is also expressed in lateral sepals whereas *OAP3-2* is also expressed in petals^6^, these genes may have other, still unknown roles in determining the morphological identity of lateral sepals or petals beyond the P code. Furthermore, L and SP complex genes are continuously expressed throughout flower development^6^, suggesting that *OAP3*, *OPI* and *OAGL6* may have additional functions during organ morphogenesis. To uncover these questions, we used virus-induced gene silencing (VIGS)-based gene knockdown to comprehensively functionally characterize the genes from the L and SP complexes and their target networks in *Phalaenopsis* orchids.

Here, we show the existence of L′ (OAP3-1/OAGL6-2/OPI) and SP’ (OAP3-2/OAGL6-1/OPI) complexes. The L′ complex has the function in regulating the structure of the lower part of the lateral sepals and helps the lips fit properly in the center of the flower. We also find that B and AGL6 proteins in P-code harbor additional function during orchid flower development. OAP3-1/OAGL6-1/OPI in SP complexes regulate anthocyanin accumulation and pigmentation whereas OAP3-2/OAGL6-2/OPI in L complexes promotes red spot formation in the perianth. Furthermore, SP complex proteins OAGL6-1 is found to be able to promote pedicel abscission whereas OAP3-1/OPI could function to suppress sepal/petal senescence.

## Results

### The finding of L′/SP′ complexes in the advanced P code

**Fig. 1:**
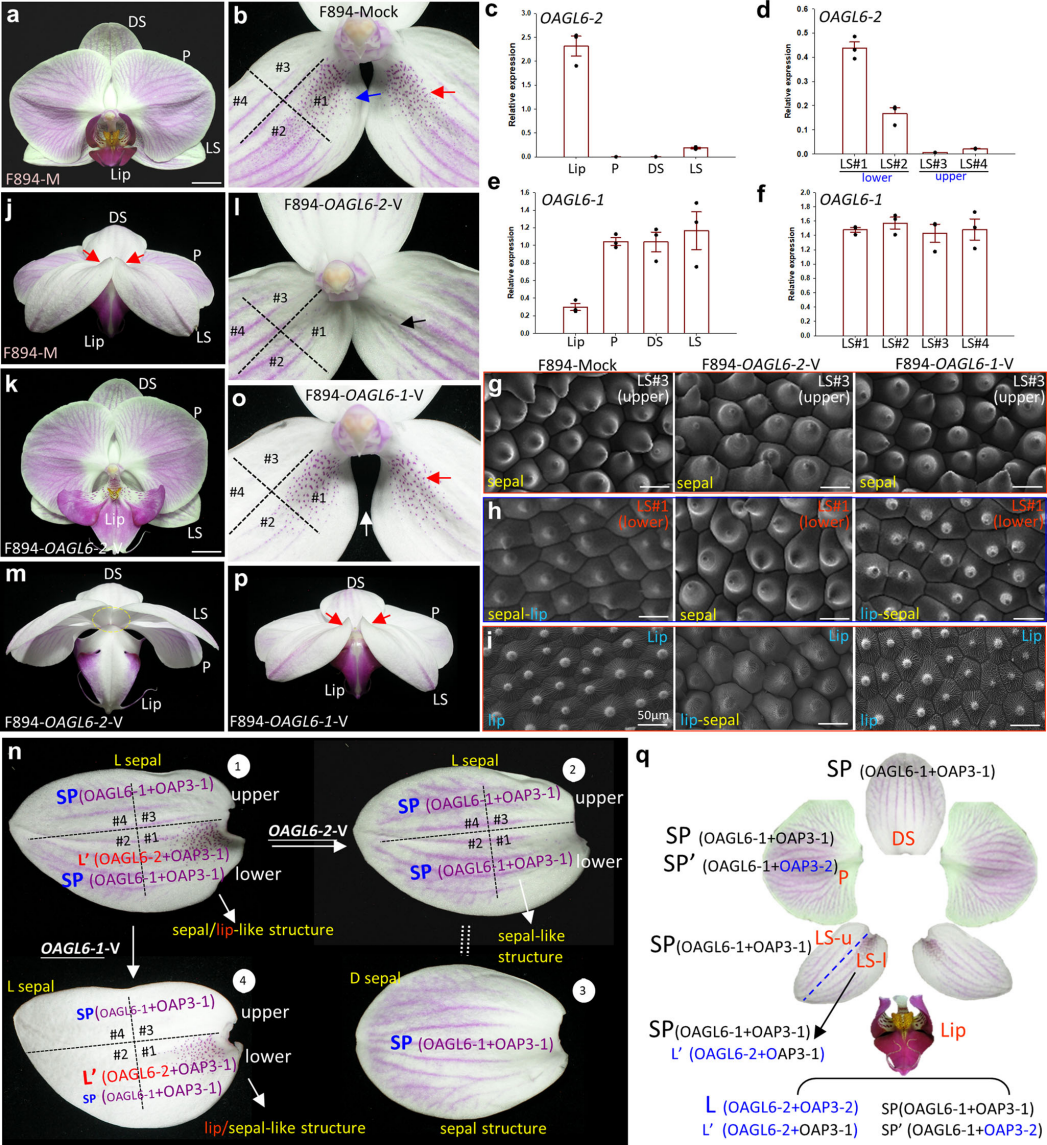
The emergence of L’ and SP’ complexes in orchid flowers revealed modified protein interactions in the P-code model. **a** A flower of wild-type control (mock) *Phalaenopsis* F894. LP lips, P petals, DS dorsal sepals, LS lateral sepals. Bars = 15 mm. **b** Close-up of the lateral sepals from (**a**), which are divided into a lower part (#1, 2) and an upper part (#3, 4). The red spots (red arrow) and curved edge (blue arrow) were observed in portion #1. **c**–**f** The expression of *OAGL6-2* (**c**) and *OAGL6-1* (**e**) in lips (Lip), petals (P), dorsal sepals (DS) and lateral sepals (LS). The expression of *OAGL6-2* (**d**) and *OAGL6-1* (**f**) in four parts of lateral sepals. Error bars show ±SEM, *n* = 3 biologically independent samples. **g** The epidermal cells of the #3 portion in the upper lateral sepals of wild-type control (mock) (left), *OAGL6-2*-VIGS (middle) and *OAGL6-1*-VIGS (right) F894 flowers showing conical cell morphology. Bar = 50 µm. **h** The epidermal cells of the #1 portion in lower lateral sepals of wild-type control (left) and *OAGL6-1*-VIGS (right) flowers exhibit a flattened sepal/lip-like cell morphology, whereas those of *OAGL6-2*-VIGS (middle) F894 exhibits conical cell morphology. Bar = 50 µm. **i** The epidermal cells of the wild-type control (left) and *OAGL6-1*-VIGS (right) lips exhibit a flattened lip-like cell morphology, whereas those of *OAGL6-2*-VIGS (middle) F894 exhibit conical cell morphology. Bar = 50 µm. In (**g**, **h**, **i**), each experiment was repeated twice independently with similar results. **j** Back view of the lateral sepals from (**a**), which show curved edges in the lower portion (red arrow). **k** A flower of *OAGL6-2*-VIGS *Phalaenopsis* F894. Bars = 15 mm. **l** Close-up of the lateral sepals from (**k**), which show flattened edges without red spots (arrow) in portion #1. **m** Back view of the lateral sepals from (**k**), which show flattened edges in the lower portion (circle). **n** Summary of the protein interactions in four different parts of the lateral sepals of wild-type control (-1), *OAGL6-2*-VIGS (-2), and *OAGL6-1*-VIGS (-4) F894 as well as dorsal sepals of wild-type control (-3). In the wild-type control (-1), the upper lateral (L) sepals have only SP complex (OAGL6-1 + OAP3-1 + OPI) function, whereas the lower lateral sepals have both high SP (OAGL6-1 + OAP3-1 + OPI) and low L’ (OAGL6-2 + OAP3-1 + OPI) complex functions, and they are converted to a sepal/lip-like structure. In *OAGL6-2*-VIGS (-2), both the upper and lower lateral (L) sepals have only SP complex (OAGL6-1 + OAP3-1 + OPI) function, and the whole lateral sepals are converted into a dorsal sepal-like structure (-3), which has only SP complex (OAGL6-1 + OAP3-1 + OPI) activity. In *OAGL6-1*-VIGS (-4), the upper lateral (L) sepals have only SP complex (OAGL6-1 + OAP3-1 + OPI) function, whereas the lower lateral sepals have low SP (OAGL6-1 + OAP3-1 + OPI) and high L’ (OAGL6-2 + OAP3-1 + OPI) complex functions, and they are converted to a more lip/sepal-like structure. **o** Close-up of the lateral sepals of an *OAGL6-1*-VIGS *Phalaenopsis* F894 flower. The red spots (red arrow) and enhanced curved edge (white arrow) were observed in portion #1. **p** Back view of the lateral sepals from (**o**), which show curved edges in the lower portion (red arrow). **q** Summary of the modified P-code protein interactions in orchid flowers. The dorsal and upper parts of the lateral sepals (LS-u) are exclusively regulated by the SP complex (OAGL6-1 + OAP3-1). The lower part of the lateral sepal (LS-l) is regulated by a strong SP complex (OAGL6-1 + OAP3-1) and a weak L’ complex (OAGL6-2 + OAP3-1). The petals (P) are regulated by both SP (OAGL6-1 + OAP3-1) and SP’ (OAGL6-1 + OAP3-2) complexes. The lips (Lip) are primarily regulated by the L complex (OAGL6-2 + OAP3-2) and, to a small degree, by the L’/SP/SP’ complexes. Source data underlying Fig. 1c–f are provided as a Source Data file.

*OAGL6*-2 in the L complex is not only highly expressed in the lip but also expressed at a very low level in the lateral sepals of orchid flowers^6^ (Fig. 1a–c). *OAGL6-2* is mainly expressed in the lower part of the lateral sepals in different *Phalaenopsis* varieties (Fig. 1d) (Supplementary Fig. 1a–o) and in various species of orchids (Supplementary Fig. 2a–r), whereas the SP complex-associated gene *OAGL6-1* is expressed evenly in all parts of the lateral sepals (Fig. 1e, f) (Supplementary Fig. 1p–t). Interestingly, *OAGL6-2*-VIGS not only caused the conversion of the lip into a petal-like structure (Fig. 1k) but also caused the loss of the distinct characteristics such as red spots or a greenish color (Fig. 1b) (Supplementary Fig. 1b, e, h, k, n) and the curved edge (Fig. 1b, j) (Supplementary Fig. 1x) on the lower part of the lateral sepals (Fig. 1l, m) (Supplementary Fig. 1y). In addition to converting lip cells into upper sepal epidermal cells (Fig. 1i, middle) (Supplementary Fig. 1w, middle), the epidermis in *OAGL6-2*-VIGS lower lateral sepals (Fig. 1h, middle) (Supplementary Fig. 1v, middle) was clearly converted into upper sepal epidermis (Fig. 1g, left and middle) (Supplementary Fig. 1u, left and middle). This result indicated that *OAGL6-2* must function in determining lower lateral sepal identity.

**Fig. 2:**
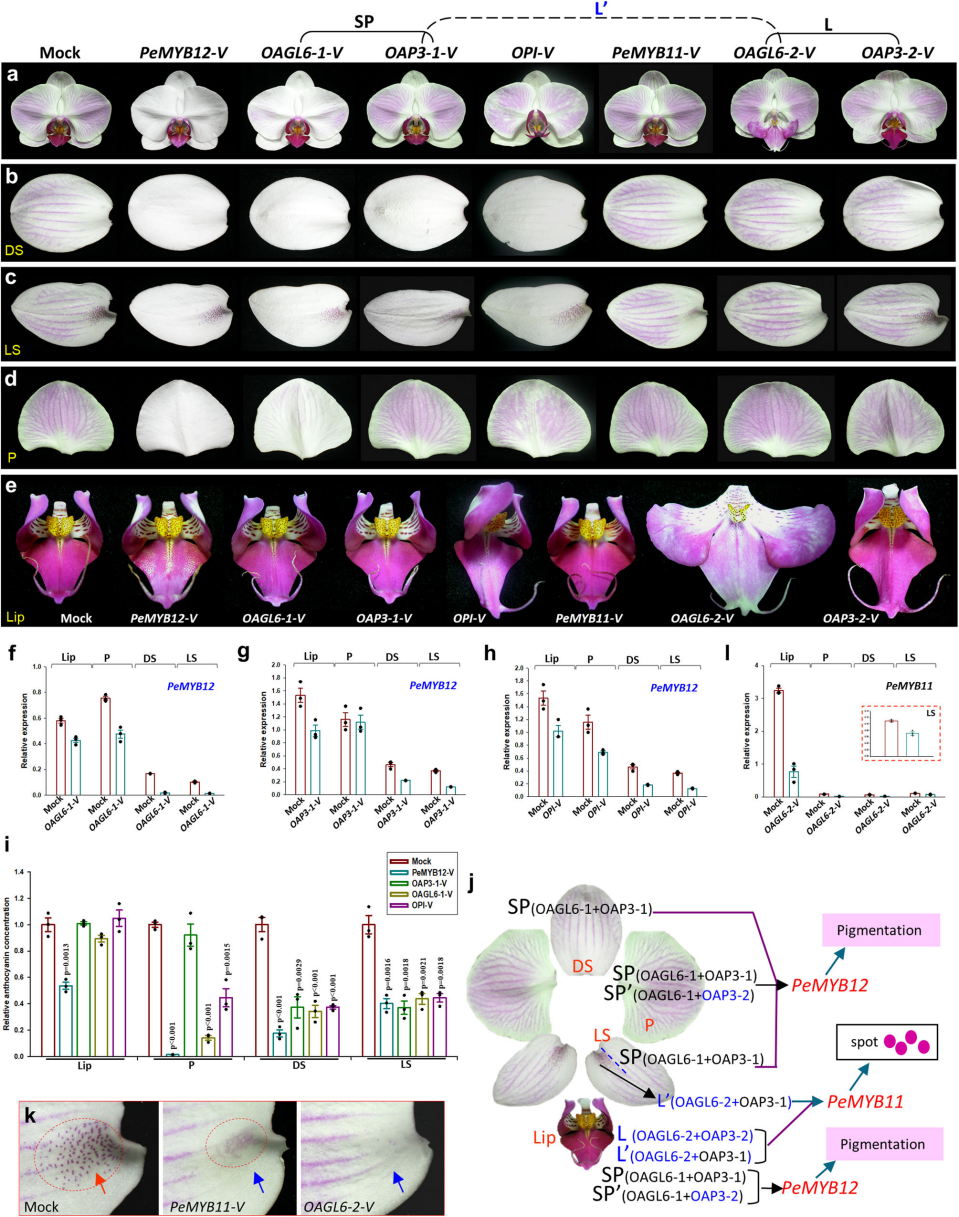
The SP/SP’ complexes regulated pigmentation, and the L/L’ complexes regulated red spot formation in orchid flowers. **a–e** Flowers (**a**), dorsal sepals (**b**), lateral sepals (**c**), petals (**d**) and lips (**e**) of wild-type control (Mock), *PeMYB12*-VIGS, *OAGL6-1*-VIGS, *OAP3-1*-VIGS, *OPI*-VIGS, *PeMYB11*-VIGS, *OAGL6-2*-VIGS and *OAP3-2*-VIGS *Phalaenopsis* F894 flowers. **f**–**h** The expression of *PeMYB12* in the lips (Lip), petals (P), dorsal sepals (DS) and lateral sepals (LS) of *OAGL6-1*-VIGS (**f**), OAP3*-1*-VIGS (**g**) and *OPI*-VIGS (**h**) F894. Error bars show ± SEM. *n* = 3 biologically independent samples. **i** The detection of anthocyanin content in the lips (Lip), petals (P), dorsal sepals (DS) and lateral sepals (LS) of wild-type control (Mock), *PeMYB12*-VIGS, *OAP3-1*-VIGS, *OAGL6-1*-VIGS and *OPI*-VIGS F894. Error bars show ±SEM. *n* = 3 biologically independent samples. The *p* value indicates significant differences from the control (Mock) value. The statistical test used was two-sided according to Student’s *t*-test. **j** Summary of the protein complexes regulating pigmentation and red spot formation in orchid flowers. The SP complex is exclusively required for the activation of *PeMYB12* expression and anthocyanin accumulation in dorsal (DS) and lateral (LS) sepals. The SP and SP’ complexes have complementary functions in regulating anthocyanin accumulation in the petals (P) and lips (Lip). The L/L’ complexes control *PeMYB11* expression and spot formation in the lower part of the lateral sepals and lips. **k** Close-up of the lower part of the lateral sepals from (**c**). The wild-type control (Mock) contained red spots (circle and red arrow) that were absent in *PeMYB11*-VIGS and *OAGL6-2*-VIGS (blue arrows). **l** The expression of *PeMYB11* in the lips (Lip), petals (P), dorsal sepals (DS) and lateral sepals (LS) of *OAGL6-2*-VIGS F894. The expression of *PeMYB11* in lateral sepals (LS) is enlarged in the box. Error bars show ±SEM. *n* = 3 biologically independent samples. Source data underlying Figs. 2f–i and 2l are provided as a Source Data file.

**Fig. 3:**
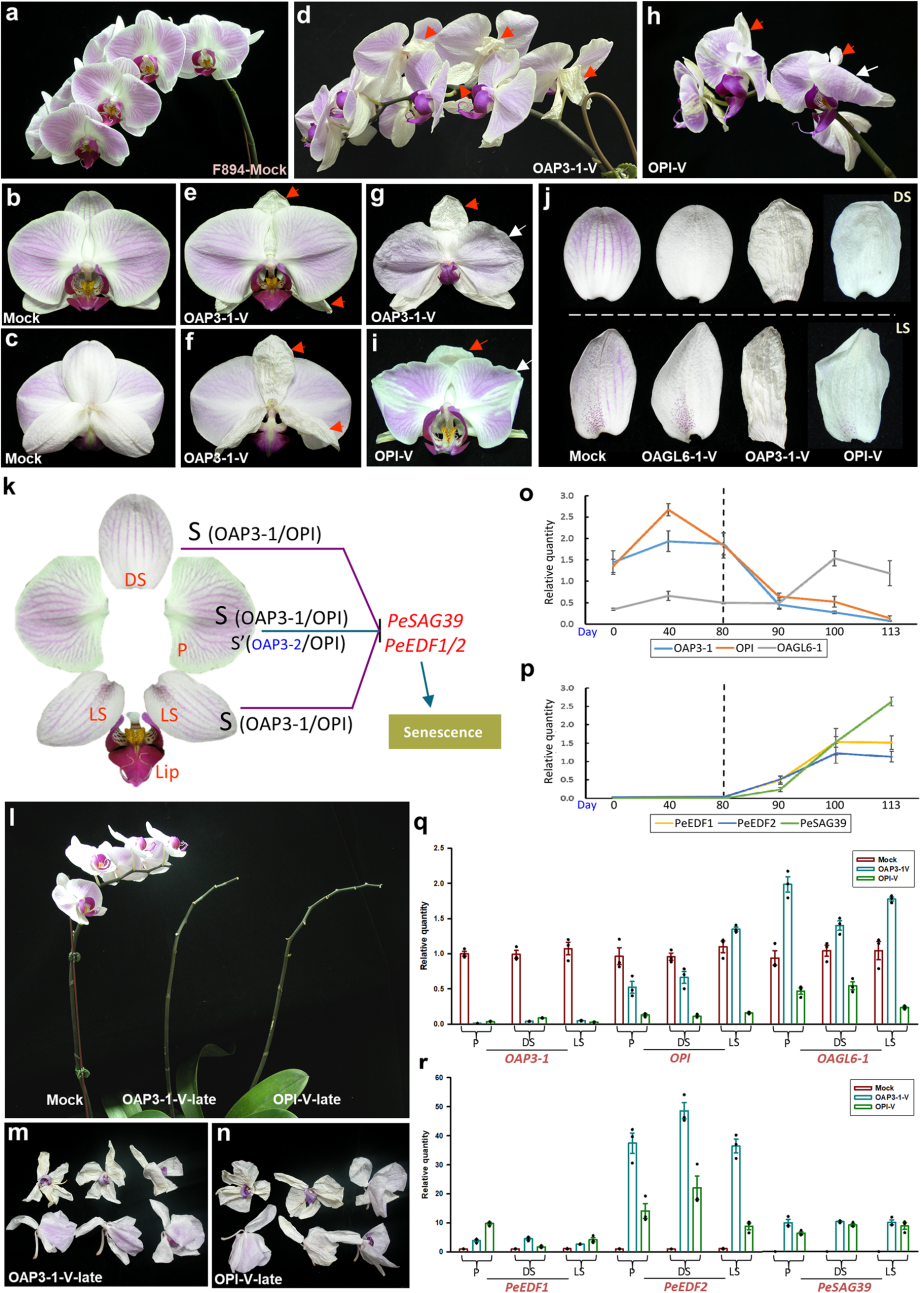
The OAP3-1 and OPI B functional proteins regulated sepal/petal senescence. **a**–**c** The inflorescence (**a**), front view (**b**) and back view (**c**) of flowers of the control (Mock) *Phalaenopsis* F894. **d**–**f** The inflorescence (**d**), front view (**e**) and back view (**f**) of flowers of *OAP3-1*-VIGS F894, which showed early senescence of the sepals (red arrows). **g** The senescence of the *OAP3-1*-VIGS petal (white arrow) occurred later than did that of the dorsal/lateral sepals (red arrow). **h**–**i** The inflorescence (**h**) and front view (**i**) of flowers of *OPI*-VIGS F894, which showed early senescence of the sepals (red arrow) and petals (white arrow). **j** The dorsal (DS) and lateral (LS) sepals of control (Mock), *OAGL6-1*-VIGS, *OAP3-1*-VIGS, and *OPI*-VIGS F894 (from left to right) at the same stage. **k** Summary of the protein complexes that regulate sepal/petal senescence in orchid flowers. The OAP3-1/OPI B functional proteins may form a senescence complex (S), which is exclusively required for the suppression of *PeSAG39/PeEDF1/2* expression and senescence in dorsal (DS) and lateral (LS) sepals. S and minor S’ (OAP3-2/OPI) complexes have complementary functions in regulating petal (P) senescence. **l**–**n** At the same stage, all the flowers were senescent and abscised from the *OAP3-1*-VIGS-late (**l**, middle) (**m**) and *OPI*-VIGS-late (**l**, right) (**n**) inflorescences, whereas the flowers of the control (Mock) inflorescences showed no sign of senescence or abscission (**l**, left). **o**–**p** Analysis of the expression profile for *OAP3-1/OPI/OAGL6-1* (**o**) and *PeSAG39/PeEDF1/2* (**p**) throughout flower development. The dashed lines indicate that the senescence of the F894 flowers starts at 80–90 days after flower opening. Error bars show ±SD. *n* = 3 biologically independent samples. **q**, **r** Analysis of the expression of *OAP3-1/OPI/OAGL6-1* (**q**) and *PeSAG39/PeEDF1/2* (**r**) in the petals (P), dorsal sepals (DS) and lateral sepals (LS) of control (Mock), *OAP3-1*-VIGS-late and *OPI*-VIGS-late F894 flowers. Error bars show ±SEM. *n* = 3 biologically independent samples. Source data underlying Fig. 3o–r are provided as a Source Data file.

How can OAGL6-2 function in determining lower lateral sepal identity without the presence of its L complex partner OAP3-2? Is it possible that OAGL6-2 interacts with OAP3-1 in the SP complex to form a new L′ complex (OAGL6-2 + OAP3-1) that has a similar function as the L complex? To test this assumption, a FRET analysis for orchid proteins was performed in tobacco leaf cells^1,9^. The result indicated that the OAGL6-2 could not only form a L complex (OAGL6-2 + OAP3-2 + OPI) (Supplementary Fig. 3a) but also likely interacts with OAP3-1 in the SP complex to form a new L′ complex (OAGL6-2 + OAP3-1) (Supplementary Fig. 3b). Thus, the lower lateral sepals likely have both high SP (OAGL6-1 + OAP3-1+OPI) and low L′ (OAGL6-2 + OAP3-1 + OPI) complex functions (Fig. 1n-1). In *OAGL6-2*-VIGS, the reduced *OAGL6*-2 in the lower lateral sepals likely caused reduced L′ complex activity and proportionally increased SP complex activity, resulting in a more upper lateral sepal structure/morphology (Fig. 1n-2) and turning the whole lateral sepals to a nearly identical dorsal sepal structure (Fig. 1n-3).

**Fig. 4:**
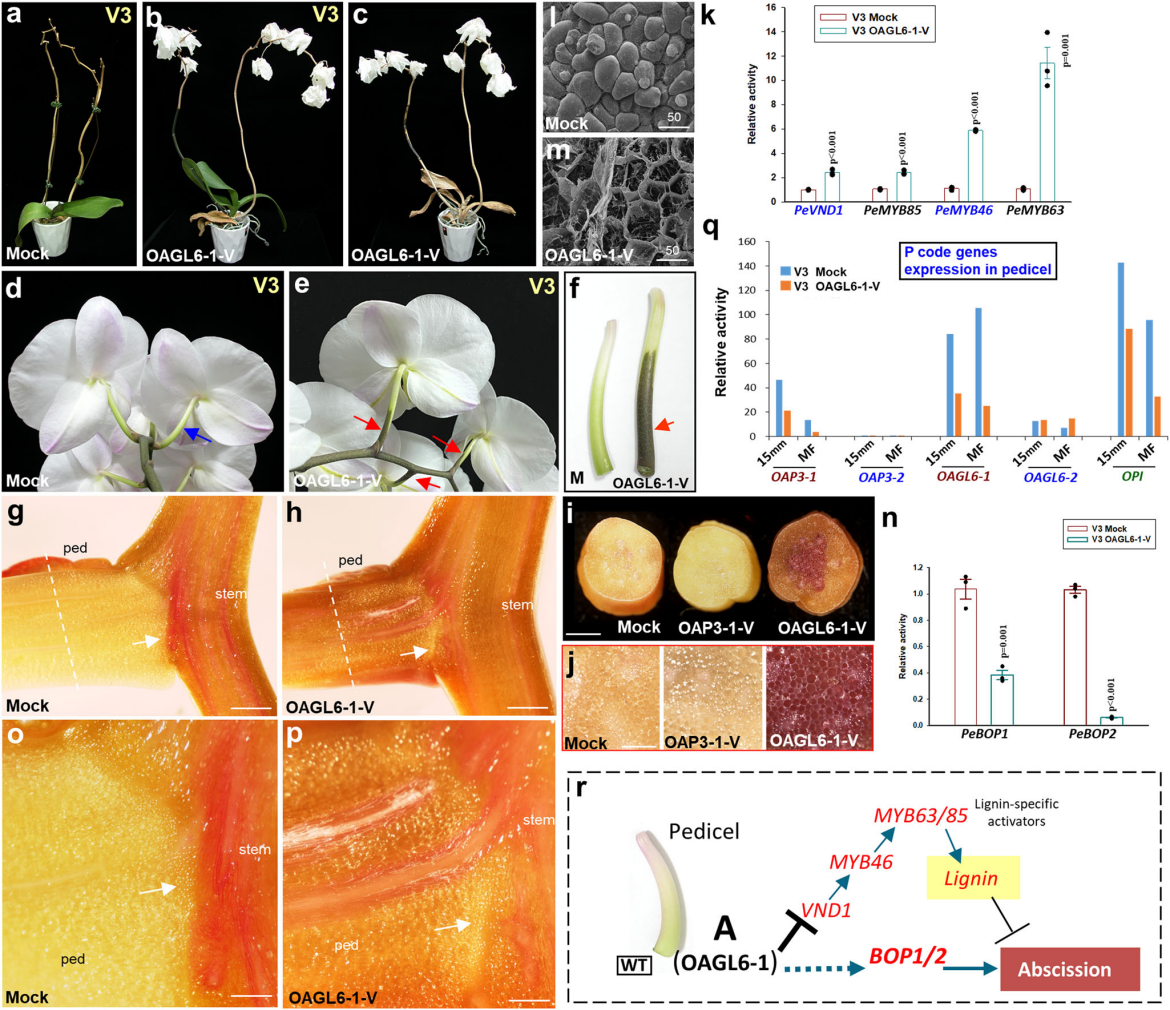
The OAGL6-1 protein regulated pedicel abscission in orchids. **a**, **b** The flowers were senescent and abscised completely from the control (Mock) inflorescence (**a**), whereas senescent flowers were not abscised from the *OAGL6-1*-VIGS inflorescence (**b**) of *Phalaenopsis* V3. **c** The senescent flowers were still not abscised three months after the plant (**b**) had completely dried. **d**–**f** The back views of flowers of the control (Mock) (**d**) and *OAGL6-1*-VIGS (**e**) *Phalaenopsis* V3. A dark brown color was observed in the pedicel of *OAGL6-1*-VIGS (red arrow in (**e**, **f**)) but was absent in the control (Mock) pedicel (blue arrow in (**d**)) (**f**). **g**–**h** The longitudinal views for pedicels (ped) and the connected stems of the control (Mock) (**g)** and *OAGL6-1*-VIGS V3 (**h**) after staining by phloroglucinol. The arrows indicate the AZ in the junction of the pedicel and stem. The dashed lines indicate the place used for the cross view in (**i**, **j**). Bars = 1 mm. **i** Cross view of the pedicels of the control (Mock), *OAP3-1*-VIGS and *OAGL6-1*-VIGS V3 (from left to right) after staining by phloroglucinol. Bars = 1 mm. **j** Close-up image of the phloroglucinol-stained pedicels from (**i**). Bars = 100 µm. **k**, **n** Analysis of the expression levels of *PeVND1*, *PeMYB85*, *PeMYB46*, and *PeMYB63* (**k**) as well as *PeBOP1* and *PeBOP2* (**n**) in the pedicels of control (Mock) and *OAGL6-1*-VIGS V3 flowers. Error bars show ±SEM. *n* = 3 biologically independent samples. The *p* value indicates significant differences from the control (Mock) value. The statistical test used was two-sided according to Student’s *t*-test. **l**, **m** SEM image of pedicel AZs in control (Mock) (**l**) and *OAGL6-1*-VIGS (**m**) V3 flowers. Bars = 50 µm. **o**, **p** Close-up of the phloroglucinol-stained junctions for the pedicel (ped) and the stem of the control (Mock) (**o**) and *OAGL6-1*-VIGS V3 (**p**) from (**g**, **h**). The arrows indicate the AZ region with the small differentiated cells formed. The cells in the AZ region in (**p**) were altered by the penetration of lignin from the pedicel to the stem. Bars = 300 µm. In (**j**, **l**, **m**, **o**, **p**), each experiment was repeated twice independently with similar results. **q** Analysis of the expression levels of *OAP3-1*, *OAP3-2*, *OAGL6-1*, *OAGL6-2*, and *OPI* in 15 mm and mature flower pedicels of control (Mock) and *OAGL6-1*-VIGS V3 flowers. The data were derived from one NGS data. **r** In wild-type orchid flowers, OAGL6-1 proteins may form an abscission complex (A) to promote the abscission of pedicels by suppressing the expression of *VND1/MYB46/63/85* and lignin formation and to ensure the normal *BOP1/2* expression and subsequent AZ formation. Source data underlying Figs. 4k, 4n, and 4q are provided as a Source Data file.

In the *OAGL6-1*-VIGS flowers, only the structure/morphology of the lower lateral sepals was affected (Fig. 1o), in which SP complex activity was reduced and in which the proportion of the L′ complex increased (Fig. 1n-4), resulting in a flatter lip-like epidermis in the lower lateral sepals (Fig. 1h-3) (Supplementary Fig. 1v-3) with an enhanced curvature of the edge (Fig. 1o, p) (Supplementary Fig. 1z). FRET analysis showed that OAGL6-1 could not only form an SP complex with OAP3-1 (Supplementary Fig. 3c) but also interacts with OAP3-2 (an L complex component) to form the SP’ complex (OAGL6-1 + OAP3-2 + OPI) (Supplementary Fig. 3d).

Our results revealed that OAGL6 proteins are the determining factors that form either the L or SP complex in controlling orchid perianth identity. OAGL6-1 determines SP (with OAP3-1) or SP′ (with OAP3-2) complexes, and OAGL6-2 determines L (with OAP3-2) or L′ (with OAP3-1) complexes. In orchid flowers (Fig. 1q), the dorsal and upper parts of lateral sepals are exclusively regulated by the SP complex. The lower part of the lateral sepals showed a sepal/lip-like morphology since it was regulated by a strong SP complex and a weak L′ complex. Petals are regulated by both SP and SP’ complexes. The lips were regulated mainly by the L complex and weakly by L′/SP/SP′ complexes.

One interesting question is why the L′ complex coexists with the SP complex in the lower lateral sepals of orchid flowers. We found that the flat lip-like epidermis (Fig. 1h-1) altered the structure/morphology and caused a curved edge in both lower lateral sepals (Fig. 1b, j, n-1) (Supplementary Fig. 1n, x), which created space to fit the lip more properly in the center, without being too prominent (Fig. 1b, j). Thus, the production of the lips should have coevolved with the emergence of the L′ complex in the lower lateral sepals during orchid flower evolution. This structural change does not occur in the dorsal sepals since their position in the flower does not affect the development of the lips.

### The SP/SP’ complexes regulate pigmentation in the perianth

A complete reduction of full-red pigmentation in the dorsal/lateral sepals was observed in *OAGL6-1*-VIGS (Fig. 2a, b, c, left 3), *OAP3-1*-VIGS (Fig. 2a, b, c, left 4) and *OPI*-VIGS (Fig. 2a, b, c, left 5) F894 flowers compared with the *Phalaenopsis* F894 control flowers (Fig. 2a, b, c, left 1), and this pigmentation was similar to that observed in *PeMYB12*-VIGS F894 plants (Fig. 2a, b, c, left 2). A significant downregulation of *PeMYB12*, which has been reported to regulate flower pigmentation and anthocyanin accumulation^20–26^, was observed in the dorsal/lateral sepals of *OAGL6-1*-VIGS (Fig. 2f), *OAP3-1*-VIGS (Fig. 2g) and *OPI*-VIGS (Fig. 2h) F894 flowers. The level of anthocyanins was clearly reduced in the *OAGL6-1*-VIGS, *OAP3-1*-VIGS F894 and *OPI*-VIGS dorsal/lateral sepals, similar to that in the *PeMYB12*-VIGS F894 flowers (Fig. 2i). Since only *OAGL6-1*, *OAP3-1* and *OPI* in SP complex expressed in the dorsal/lateral sepals, indicating that the SP complex (OAGL6-1 + OAP3-1 + OPI) is exclusively required for anthocyanin accumulation in dorsal/lateral sepals by activating the *PeMYB12* expression (Fig. 2j). Silencing of either *OAGL6-1*, *OAP3-1* or *OPI* expression caused the same disruption of the SP complex and the reduction in pigmentation in dorsal/lateral sepals (Fig. 2b, c, left 3 to 5). In contrast, silencing of *OAGL6-2* or *OAP3-2* expression in the L complex did not affect the pigmentation (Fig. 2a, b, c, left 7 and 8) in the dorsal/lateral sepals.

Different from that observed in dorsal/lateral sepals, a significant and relative weak reduction in full-red pigmentation, downregulation of *PeMYB12* expression and accumulation of anthocyanins in petals was observed in *OAGL6-1*-VIGS (Fig. 2d, left 3, f, i) and *OPI*-VIGS F894 flowers (Fig. 2d, left 5, h, i) and not in *OAP3-1*-VIGS (Fig. 2d, left 4, g, i) or *OAP3-2*-VIGS (Fig. 2d, left 8) F894 flowers. This result indicated that silencing of *OAP3-1* or *OAP3-2* expression caused the disruption of only the function of either the SP or SP’ complex and had no or very weak effect on the reduction in pigmentation in the petals (Fig. 2d, left 4 and 8). This result actually supported the coexistence of SP and SP’ complexes in petals, which have complementary functions in regulating both petal identity and pigmentation (Figs. 1q, 2j).

Silencing of *OAGL6-1* (Fig. 2e, left 3), *OAP3-1* (Fig. 2e, left 4), *OPI* (Fig. 2e, left 5) and *OAP3-2* (Fig. 2e, left 8) caused a similar weaker reduction in full-red pigmentation, *PeMYB12* expression (Fig. 2f, g, h) and anthocyanin accumulation (Fig. 2i) in the lips than did the silencing of *PeMYB12-VIGS* (Fig. 2e, left 2, 2i). This result revealed that SP and SP’ complexes are involved in the regulation of anthocyanin accumulation in the lips, although to different degrees (Fig. 2j).

We also examined the VIGS of *Phalaenopsis* KA38501, which has yellow/red-orange perianth pigmentation (Supplementary Fig. 4a, top/left). Similar to *PeMYB12*-VIGS KA38501 flowers (Supplementary Fig. 4a, top/middle and top/right), a reduction in red-orange pigmentation and anthocyanin content in the dorsal/lateral sepals of *OAGL6-1/OAP3-1/OPI*-VIGS KA38501 (Supplementary Fig. 4a, bottom, b, c, e) and in the flower petals of *OAGL6-1/OPI*-VIGS KA38501 (Supplementary Fig. 4a, bottom/left, bottom/right, d, e), as well as a relatively weak effect on lips of *OAGL6-1/OAP3-1/OPI*-VIGS KA38501 (Supplementary Fig. 4a, e) flowers, were observed. These results strongly support that SP and SP’ complexes are involved in the regulation of anthocyanin accumulation and pigmentation in the orchid perianth.

### The L′ complex regulates red spot formation

In addition to causing the conversion of lips to sepal/petal-like structures (Fig. 2a, e, left 7), *OAGL6-2*-VIGS also resulted in a significant reduction in red spots (Fig. 2k, left) in the lateral sepals (Figs. 1l, 2c, left 7, k, right), similar to that observed in *PeMYB11*-VIGS (Fig. 2c, left 6, k, middle) *Phalaenopsis* F894 flowers. Interestingly, similar to that which occurred in *OAGL6-2* (Fig. 1c), *PeMYB11* was also exclusively expressed in the lips (high) and lateral sepals (low) (Fig. 2l), and its expression was significantly downregulated in the *OAGL6-2*-VIGS flowers (Fig. 2l). This result revealed that OAGL6-2 formed an L′ complex with OAP3-1 in the lateral sepals; in turn, this complex controls the activation of *PeMYB11* (Fig. 2j), which has been thought to be associated with red spot formation^22,27^. Silencing *OAGL6-2* caused a severe reduction in L′ complex function, subsequently downregulating *PeMYB11* expression (Fig. 2l) and reducing red spot formation (Fig. 2c, left 7, k, right). Since *OAP3-1* was expressed at a much higher level than *OAGL6-2* was in the lateral sepals, the reduced amount of *OAP3-1* in the *OAP3-1*-VIGS lateral sepals is still able to form enough numbers of L′ complexes with a low amount of *OAGL6-2*, with no effect on the red spot formation (Fig. 2c, left 4). This result supported that OAGL6-2 is the major component in the formation of L/L′ complexes in the control of *PeMYB11* expression in lateral sepal and lips (Fig. 2j). Red spot formation was not affected in the *OAGL6-1*-VIGS lateral sepals (Fig. 2c, left 3, 1o), indicating the SP complex is not involved in regulating this process (Fig. 2j).

### OAP3-1 and OPI suppress senescence of sepals/petals

In addition to see the alteration in pigmentation, significantly earlier senescence for the dorsal/lateral sepals compared with that of the control (Fig. 3a–c) was observed for the *OAP3-1*-VIGS (Fig. 3d–f) F894 flowers. In the controls, the senescence of the sepals and petals occurred simultaneously. The senescence of the *OAP3-1*-VIGS petals occurred later than that of the dorsal/lateral sepals (Fig. 3d–f), although it occurred earlier than that of the control F894 petals (Fig. 3g). Similar early senescence for both dorsal/lateral sepals and petals was observed in *OPI*-VIGS flowers (Fig. 3h–j). This early senescence phenotype was not observed in *OAGL6-1*-VIGS (Fig. 3j), *OAP3-2*-VIGS or *OAGL6-2*-VIGS flowers. These results indicated that only the B class proteins (OAP3-1 + OPI) from the SP complex may form an independent senescence-associated complex (S complex) in suppressing sepal/petal senescence in orchids (Fig. 3k). Since the senescence of the petals occurred later than that of the sepals in *OAP3-1*-VIGS flowers (Fig. 3d–f), OAP3-2+OPI from the SP′ complex might form an S’ complex in petals with a complementary function in suppressing petal senescence with the SP complex, albeit in a weaker manner (Fig. 3k).

To avoid any influence on flower senescence due to the alteration of flower organ identity in VIGS of *OAP3-1/OPI*, a VIGS experiment was performed by late infection after flower organs were evaluated during the later stage (VIGS-late). When all the flowers were senescent and had abscised from the *OAP3-1*-VIGS-late (Fig. 3l, m) or *OPI*-VIGS-late (Fig. 3l, n) F894 inflorescences, the flowers of the control inflorescences did not yet show senescence (Fig. 3l). A similar early senescence phenotype was also observed in the *OAP3-1*-VIGS-late and *OPI*-VIGS-late V3 (Supplementary Fig. 5) and KA38501 (Supplementary Fig. 6) *Phalaenopsis* flowers. This result supports that orchid B class proteins (OAP3-1 + OPI) suppress the senescence of sepals/petals (Fig. 3k).

In wild-type F894, flower senescence starts at 80–90 days after flower opening (AFO). Interestingly, *OAP3-1/OPI* expression starts to decrease at 80 days AFO and is continuously downregulated afterward to a basal level until flower senescence is complete at 110 days AFO (Fig. 3o). In contrast, an opposite expression pattern, starting to increase at 80 days AFO until reaching the highest level at 110 days AFO, was observed for the senescence-associated gene *PeSAG39*^28^ and for downstream genes involved in ethylene signaling (*PeEDF1/*2)^29–31^ (Fig. 3p). A similar downregulation of *OAP3-1/OPI* (Fig. 3q) and upregulation of *PeSAG39/PeEDF1/2* (Fig. 3r) was observed in the sepals/petals of both *OAP3-1*-VIGS-late and *OPI*-VIGS-late flowers. A similar gene expression profiles were also observed in the *OAP3-1*-VIGS-late and *OPI*-VIGS-late V3 (Supplementary Fig. 5) and KA38501 (Supplementary Fig. 6) *Phalaenopsis* flowers. These results support that *OAP3-1* and *OPI* function in suppressing orchid flower senescence by negatively regulating *PeSAG39/PeEDF1/2*.

The expression of *OAGL6-1* continuously increased 90 days AFO (Fig. 3o) during flower development, which is opposite that of *OAP3-1/OPI*, and *OAGL6-1* expression only slightly decreased or even increased in *OAP3-1/OPI*-VIGS-late senescent flowers (Fig. 3q), respectively, further supporting that *OAGL6*-1 in the SP complex is not involved in the regulation of orchid flower senescence.

### OAGL6-1 regulates the abscission of pedicels

A significant defect in abscission of the pedicel was observed in *OAGL6-1*-VIGS *Phalaenopsis* V3 (Fig. 4b, c) and F894 (Supplementary Fig. 7a) flowers. In contrast to the flowers of the control plants (Fig. 4a) (Supplementary Fig. 7b), all the senescent *OAGL6-1*-VIGS flowers remained attached to the inflorescence (Fig. 4b) (Supplementary Fig. 7a), even after the plants were completely dry and had died (Fig. 4c).

A darker brown color in the pedicel of *OAGL6-1*-VIGS *Phalaenopsis* V3 (Fig. 4e, f) and F894 (Supplementary Fig. 7a, c, d, f) flowers than in the control flowers (Fig. 4d, f) (Supplementary Fig. 7e, f) was observed. When the pedicel was treated with phloroglucinol staining, which turns lignin a dark red color^32^, a significantly darker red color was observed in the pedicels of *OAGL6-1*-VIGS *Phalaenopsis* V3 (Fig. 4h, i, j, right) and F894 (Supplementary Fig. 7g) flowers than in the control flowers (Fig. 4g, i, j, left) (Supplementary Fig. 7g) and the *OAP3-1*-VIGS (Fig. 4i, j, middle) (Supplementary Fig. 7g) pedicel. The strong lignin formation in the *OAGL6-1*-VIGS *Phalaenopsis* V3 pedicel was further confirmed by the strong yellow color staining by Auramine O (Supplementary Fig. 8a, b). This dark red or yellow color for lignin staining clearly extended throughout the pedicel and ran through into the stem of the *OAGL6-1*-VIGS *Phalaenopsis* V3 (Fig. 4h, Supplementary Fig. 8b). In contrast, no dark red or yellow color for lignin staining was observed throughout the pedicel until the junction of the stem in the control flower (Fig. 4g, Supplementary Fig. 8a). Furthermore, the expression levels of the secondary wall NAC master switch *PeVND1*, the secondary wall MYB master switch *PeMYB46* and the lignin-specific activator *PeMYB63/85* (involved in the lignin biosynthetic pathway)^33–39^ were significantly upregulated in the pedicel of *OAGL6-1*-VIGS flowers (Fig. 4k) (Supplementary Fig. 7h).

In the control abscised pedicel, a fully developed abscission zone (AZ) with cells displaying a fully rounded, intact appearance was observed (Fig. 4l) (Supplementary Fig. 7i). In contrast, upon manual pedicel removal from the senescent flower, broken cells at the fracture plane and a flattened cavity were observed in the *OAGL6-1*-VIGS pedicel AZs (Fig. 4m). The expression levels of *PeBOP1*/*2*, which are necessary for the formation of the floral AZ^40,41^, were significantly downregulated in the *OAGL6-1*-VIGS pedicel (Fig. 4n) (Supplementary Fig. 7j). Further analysis indicated that small differentiated cells that formed in the AZ of the control flower pedicel (Fig. 4o, Supplementary Fig. 8c, e) were also observed in the junction of the pedicel and stem in the *OAGL6-1*-VIGS pedicel (Fig. 4p, Supplementary Fig. 8d); however, their formation was clearly altered by the penetration of lignin throughout the pedicel to the stem (Fig. 4p, Supplementary Fig. 8d, f). These results revealed that the defect in abscission of the *OAGL6-1*-VIGS pedicel was likely due to the strong increase in lignin formation in the pedicel, which tightly connected the pedicel to the stem and subsequently prevented the abscission of the pedicel from the stem. During this process, AZ was likely formed, although its further development and function were altered.

When the expression levels of all the P-code genes in the pedicels were analyzed, the expression levels were *OAGL6-1* (SP) = *OPI* (SP, L) > *OAP3-1* (SP) > *OAGL6-2* (L), and expression was not detected for *OAP3-2* (L) (Fig. 4q). Similar expression patterns for *OAGL6-1*/*OAP3-1/OAP3-2* were also observed in the pedicels of various species of orchids (Supplementary Fig. 9). Since defects in pedicel abscission were not observed in VIGS *Phalaenopsis* flowers with the silencing of the other P-code genes (*OAP3-1/OPI/OAGL6-2*/*OAP3-2*), *OAGL6-1* is likely exclusively required for the regulation of pedicel abscission. Our results revealed that the OAGL6-1 protein has an additional function to ensure the final abscission of the pedicel through the downregulation of lignin formation and subsequent normal AZ development (Fig. 4r). In the *OAGL6-1*-VIGS pedicel, *OAGL6-1* knockdown caused the upregulation of lignin biosynthetic genes and a high level of lignin in the pedicel, which altered AZ development and resulted in the strong blocking of pedicel abscission.

## Discussion

**Fig. 5:**
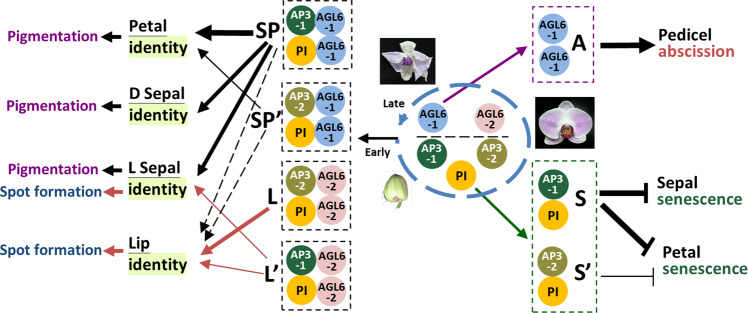
The multifunctional evolution of P-code genes in orchids. In orchids, two *AGL6* (*OAGL6-1*, *-2*), two *AP3* (*OAP3-1*, *-2*) and one *PI* (*OPI*) gene play key roles (blue dashed circle) in regulating perianth identity and other developmental processes in flowers. During early flower initiation, the L (OAP3-2/OAGL6-2/OPI) complex determines the lips, whereas the SP (OAP3-1/OAGL6-1/OPI) complex determines sepal/petal identity. In addition, an L’ complex (OAP3-1/OAGL6-2/OPI) is also likely involved in regulating the lips and lower parts of the lateral sepal identity/morphology, whereas an SP’ complex (OAP3-2/OAGL6-1/OPI) is likely involved in regulating the petal and lip identity/morphology. After flower organ identity has been determined, SP/SP’ complexes have an additional function in positively regulating (black arrow→) anthocyanin accumulation and pigmentation in sepals/petals, whereas L’/L complexes can regulate (red arrow→) red spot formation in lateral sepals and lips. During flower development, the OAP3-1/OPI B class proteins from the SP complex could form a separate senescence complex (S) to suppress (⊣) the senescence of the sepal/petal in orchids. An S’ complex (OAP3-2/OPI/X) with minor complementary function for the S complex could suppress (⊣) petal senescence. The orchid OAGL6-1 from the SP complex may form an abscission complex (A) to ensure (→) the abscission of the pedicel from senescent flowers during the late stage of flower development. AGL6-1: OAGL6-1, AGL6-2: OAGL6-2, AP3-1: OAP3-1, AP3-2: OAP3-2, PI: OPI.

B and AGL6 proteins form L (OAP3-2/OAGL6-2/OPI) and SP (OAP3-1/OAGL6-1/OPI) complexes to determine lip and sepal/petal identities in orchids^6^ (Fig. 5). This study further showed the involvement of the proposed L′ complex (OAP3-1/OAGL6-2/OPI) in regulating the lower part of the lateral sepals and lip identity/morphology during orchid flower evolution to help the evolved lips fit properly in the center of the flowers (Fig. 5). In addition, the SP complex (OAP3-1/OAGL6-1/OPI) was found to regulate anthocyanin accumulation and pigmentation in sepals/petals, whereas an SP’ complex (OAP3-2/OAGL6-1/OPI) with the complementary function was identified in the petals (Fig. 5). L′/L complexes regulate the formation of red spots in the sepals/lips (Fig. 5). The roles of B and *AGL6* genes in the regulation of flower color have never been experimentally verified in plants. We found that anthocyanin accumulation is correlated with *LMYB12* but not *LAGL6/LAP3/LPI* expression in lily tepals (Supplementary Fig. 10), suggesting a possibly unique role for orchid *OAGL6-1/OAP3-1/OPI* in regulating flower pigmentation during evolution.

Unexpectedly, B functional *OAP3-1* and *OPI* could function separately from SP complex in suppressing the senescence of the sepals/petals of orchids, whereas OAP3-2/OPI with complementary function to that of the OAP3-1/OPI is present in the petals (Fig. 5). The ability of *AP3/PI* orthologs to regulate flower organ senescence is surprising. A similar downregulation of *AP3/PI* expression during petal/tepal development/senescence was also observed in other various species of orchids (Supplementary Fig. 11), the dicot *Arabidopsis* and the monocot lily (Supplementary Fig. 12), revealing the possibly conserved role of *AP3/PI* orthologs in regulating flower senescence in flowering plants throughout evolution. The finding of the functions for B genes outside of floral organ identity specification is interesting. In addition to the orchid *OAP3-1/OPI* in this study, it has been reported that mutation in the apple B-function *PI* ortholog *MdPI* resulted in the production of parthenocarpic fruit^42^. Thus, evolutionary modification of the functions for the B class genes should occur and remains to be explored in more plant species.

We found that OAGL6-1 in the SP complex also has additional features. The orchid OAGL6-1 could function to promote the abscission of the pedicel (Fig. 5). This function is clearly independent from that of the B proteins OAP3-1/OPI in the SP complex (Fig. 5). Since orchid flowers abscised from the pedicel, which is different from the process in plant species such as *Arabidopsis* and lily (in which abscission occurs from the base of the flower organs), *OAGL6-1* orthologs may have evolved an additional expression pattern in the pedicel to control lignin levels during the evolution of this type of flower and to further develop this additional function to control pedicel abscission. Interestingly, it has been reported that two E-function SEP proteins (SLMBP21 and SEP4/J2) were involved in the regulation of pedicel abscission zone development in tomato^43,44^. Mutations in *SLMBP21* or *SEP4/J2* caused the lack of the abscission zone on the pedicels (jointless pedicels) of flowers^43,44^. However, the mechanism regulated by *OAGL6-1* in orchids is clearly different from that for tomato *SLMBP21*, since *OAGL6-1* suppressed lignin levels in pedicels whereas *SLMBP21* affected common meristem activity genes such as *LeWUS* and *LATERAL SUPPRESSOR* in the AZ^43^.

In conclusion, a scenario was proposed to elucidate the evolutionary modification of the functions of the B class *AP3*/*PI* and *AGL6* genes. In the eudicot *Arabidopsis*, AP3/PI proteins form an SP-like complex with A/E proteins in regulating petal formation. In the monocots lily (Liliales) and orchid (Orchidaceae), the AP3/PI proteins specifically interact with the AGL6-like protein to form SP-like complexes in lily or SP/SP′L/L′ complexes in orchid to further regulate tepal (lily) or sepal/petal/lip (orchids) identity. AP3/PI proteins also likely evolved to have a conserved function in *Arabidopsis*, lily and orchid in regulating petal (*Arabidopsis*), tepal (lily) and sepal/petal (orchid) senescence. During orchid flower evolution, AP3/PI/AGL6 in SP/SP′/L/L′ complexes further developed a function involved in the specific regulation of pigmentation in the perianth, and AGL6-1 in the SP complex acquired an additional function in controlling pedicel abscission, which is absent in *Arabidopsis* and lily.

## Methods

### Plant materials

Species and cultivars of orchids used in this study, including the moth orchids (*Phalaenopsis* Sogo Yukidian ‘V3’, *P*. F894, *P*. A09705, *P*. A09617, *P*. A07068, *P*. KA38501), *Oncidium spp., Cymbidium spp., Dendrobium spp., Cattleya spp*., *Brassocattleya spp., Neofinetia spp., Epidendrum spp., Paphiopedilum spp*. (lady slipper orchids), were maintained in the greenhouse of National Chung-Hsing University, Taichung, Taiwan. Plants of Asiatic hybrid lilies (*Lilium spp*.) used in this study were grown in the field in Houli, Taiwan. The *Arabidopsis etr1-1* (CS237) *Arabidopsis* seeds were obtained from Dr. Long-Chi Wang, Department of Life Science, National Chung Hsing University, Taiwan.

### Real-time PCR analysis

Quantitative real-time PCR was conducted using a Mini OpticonReal-Time PCR Detection System and Optical System Software version 3.0a (Bio-Rad Laboratories). For transcript measurements, the KAPA SYBR® FAST qPCRMaster Mix Universal (KAPA BIOSYSTEMS) was used. The amplification condition was one cycle at 95 °C for 1 min, followed by 40 cycles of 95 °C (for 15 s), 58 °C (for 15 s), and 72 °C (for 30 s), and plate reading after each cycle. The name and the sequence of the gene-specific primers for MADS box genes (*OAP3-1, OAP3-2, OAGL6-1, OAGL6-2, OPI*) of different orchids, *PeMYB12, PeMYB11, PeSAG39, PeEDF1, PeEDF2, PeVND1, PeMYB85, PeMYB46, PeMYB63, PeBOP1* and *PeBOP2* of orchids, *LMYB12, LAGL6, LMADS1/LAP3, LMADS8/LPI, LMADS9/LPI* of lily, *AtAP3* and *AtPI* of *Arabidopsis* were listed in Supplementary Tables 1 to 2. The data were analyzed using CFX Manager^TM^Software (Version 3.0; Bio-Rad Laboratories, Inc.). The transcript levels for genes were determined using three replicates and were normalized using reference genes *ACTIN* for Phalaenopsis (*PACT4*)^6,45,46^, *α-tubulin* for other orchids (*OnAT*)^6,47^, *ACTIN* for lily (*LilyACTIN*)^48^ and *UBQ10* for *Arabidopsis* (*At UBQ10*)^49^ (Supplementary Table 2).

### Anthocyanin content analysis

Total anthocyanin content in the flower samples was determined according to the spectrophotometric method described previously^50^ with slight modifications. Briefly, 100 mg of fresh flower tissues were homogenized in liquid nitrogen and added to 1 ml of 3 M HCl:H_2_O:MeOH (1:3:16, by volume). The crude extracts were agitated gently in the dark for 24 h at 4 °C and then centrifuged at 21,000 × *g* for 15 min. Anthocyanin levels in the supernatant were measured as *A*_530_ – 0.24 *A*_653_ by the spectrophotometer TECAN Infinite M200PRO with The i-control microplate reader software (Mannedorf, Switzerland)^51^.

### Phloroglucinol-HCl staining

The junctions for the *Phalaenopsis* flower pedicel and stem were longitudinally cut, whereas 2-mm-thick *Phalaenopsis* pedicle slices were crossly cut from the base of the pedicel using a blade and put into 24-well plates. The staining solution contained 0.3 g phloroglucinol in 10 ml absolute ethanol and mixed with 5 ml concentrated HCl (37%). The sample slices were filled with staining solution and covered for 5 min, followed by changing the staining solution with water.

### Calcofluor White and Auramine O staining

The Phalaenopsis stems connected with the flower pedicel were collected and fixed with the fixative FAA. Samples were decolorized by alcohol dehydration and dissected in longitudinal sections using a blade. Tissue samples were mounted in ClearSee for one day and then stained with 0.1% Calcofluor White and 0.1% Auramine O (in ClearSee) overnight^52^. The tissue samples were washed in ClearSee for 2 h. Image acquisition was performed using an Olympus FV3000 confocal microscope (Nikon, Japan), and Calcofluor White used 405 nm excitation and was detected at 430–450 nm for cellulose (cyan color), while Auramine O used 488 nm excitation and was detected at 500–600 nm for lignin and cuticle (yellow color).

### Cryo-scanning electron microscopy

The procedure used for Cryo-SEM was described in a previous study^6^. Briefly, the perianths were dissected and frozen using liquid nitrogen and transferred to the sample preparation chamber at −160 °C. The samples were etched for 10 min at −85 °C and were observed under a cryo-scanning electron microscope after gold coating (FEI Quanta 200 SEM, Quorum Cryo System PP2000TR FEI; FEI Company, Hillsboro, Oregon).

### FRET analysis

The procedure used for FRET analysis was described in the previous studies^6,18^. Briefly, to fuse OAGL6-1/OAP3-1/OAGL6-2/OAP3-2/OPI with CFP or YFP, the cDNAs for *Phalaenopsis OAGL6-1/OAP3-1/OAGL6-2/OAP3-2/OPI* were obtained by PCR amplification using gene-specific primers (Supplementary Table 3) and cloned into the pEpyon-36K and pEpyon-37K vectors upstream of the CFP or YFP sequence under the control of the CaMV 35S promoter. The above constructs were transformed into the *Agrobacterium* strain C58C1. Different ectopic proteins were expressed in tobacco leaf cells through *Agrobacterium*-infiltrated *N. benthamiana* leaves by being infiltrated in 10 mM MgCl2 at room temperature until immersed. A confocal microscope was used to detect the fluorescence signals in the nucleus. The procedure used to visualize fluorophores was described in a previous study^9^. Briefly, an Olympus FV1000 confocal microscope (Olympus FV1000, Tokyo, Japan) and the soft FV-ASW 3.0 software were used to visualize fluorophores and to calculate the raw FRET and FRET efficiency values. The mean value of FRET efficiency in the nucleus was calculated to evaluate the variation in protein interaction distances among different protein complexes (*n* > 4).

### Virus-induced gene silencing experiment

Since two DNA fragments in different independent nonconserved regions used for each of the *OAGL6-2*-VIGS, *OAGL6-1*-VIGS, *OAP3-1*-VIGS, *OAP3-2*-VIGS, *OPI-*VIGS, *PeMYB12*-VIGS, and *PeMYB11-*VIGS experiments generated the similar mutant phenotypes (Supplementary Figs. 13–19), we thus used one specific DNA fragment for each target gene in subsequent VIGS experiments. To ensure the specificity of VIGS silencing effects for each target gene, DNA fragments contained the sequences specific for *OAGL6-1 (PeMADS10), OAGL6-2 (PeMADS9), OAP3-1 (PeMADS2), OAP3-2 (PeMADS3)*, and *OPI (PeMADS6)* in the highly variable C-terminal region (Supplementary Figs. 20, 21), and for *PeMYB12* and *PeMYB11*, the variable regions located downstream from the highly conserved R2R3 MYB DNA-binding domain (Supplementary Fig. 22) were obtained by PCR amplification using gene-specific primers (Supplementary Table 4) and were inserted into the VIGS vector pCymMV-Gateway^53^. The *att*B sites (sequences in bold letters in Supplementary Table 3) were for in vitro recombination with *att*P sites in the VIGS vector pCymMV-Gateway to generate recombinant clones using Gateway^®^ BP Clonase II Enzyme Mix (Invitrogen™, Life Technologies, Carlsbad, CA, USA). pCymMV-Gateway-OAGL6-1, pCymMV-Gateway-OAGL6-2, pCymMV-Gateway-OAP3-1, pCymMV-Gateway-OAP3-2, pCymMV-Gateway-OPI, pCymMV-Gateway-PeMYB12, pCymMV-Gateway-PeMYB11 and the empty pCymMV-Gateway as a control were transformed into *Agrobacterium tumefaciens* EHA105 for further inoculation. The leaf infiltration experiments in *Phalaenopsis* orchids were performed as described previously^6,54^. Briefly, suspensions were injected into the leaf just above the site where the inflorescence emerged. At least three plants were inoculated with each pCymMV-Gateway construct for every infiltration. Flower samples were collected and analyzed at 45 DPI (days post inoculation), when the last bud of the *Phalaenopsis* inflorescences bloomed. The total number of VIGS experiments and plants analyzed for *OAGL6-1 (PeMADS10), OAGL6-2 (PeMADS9), OAP3-1 (PeMADS2), OAP3-2 (PeMADS3), OPI (PeMADS6), PeMYB12*, and *PeMYB11* in three different varieties of *Phalaenopsis* orchids are listed in Supplementary Tables 5–7.

### Reporting summary

Further information on research design is available in the Nature Research Reporting Summary linked to this article.

## Supplementary information

Supplementary Information

Peer Review File

Reporting Summary

## Data Availability

The data supporting the findings of this work are available within the paper and the Supplementary Information files. A reporting summary for this article is available as a Supplementary Information file. The data sets generated and analyzed during this study are available from the corresponding author upon request. Source data are provided with this paper.

## Source data

Source Data
