# Supplementary material for: Multifunctional evolution of B and AGL6 MADS box genes in orchids
Source: Nat Commun. 2021 Feb 10;12:902. doi: 10.1038/s41467-021-21229-w (PMC7876132; doi:10.1038/s41467-021-21229-w)
Supplement: Supplementary file 1 — Supplementary Information [file 41467_2021_21229_MOESM1_ESM.pdf]

Multifunctional evolution of B and *AGL6* MADS box genes in orchids

Hsu *et al.*

Supplementary Fig. 1

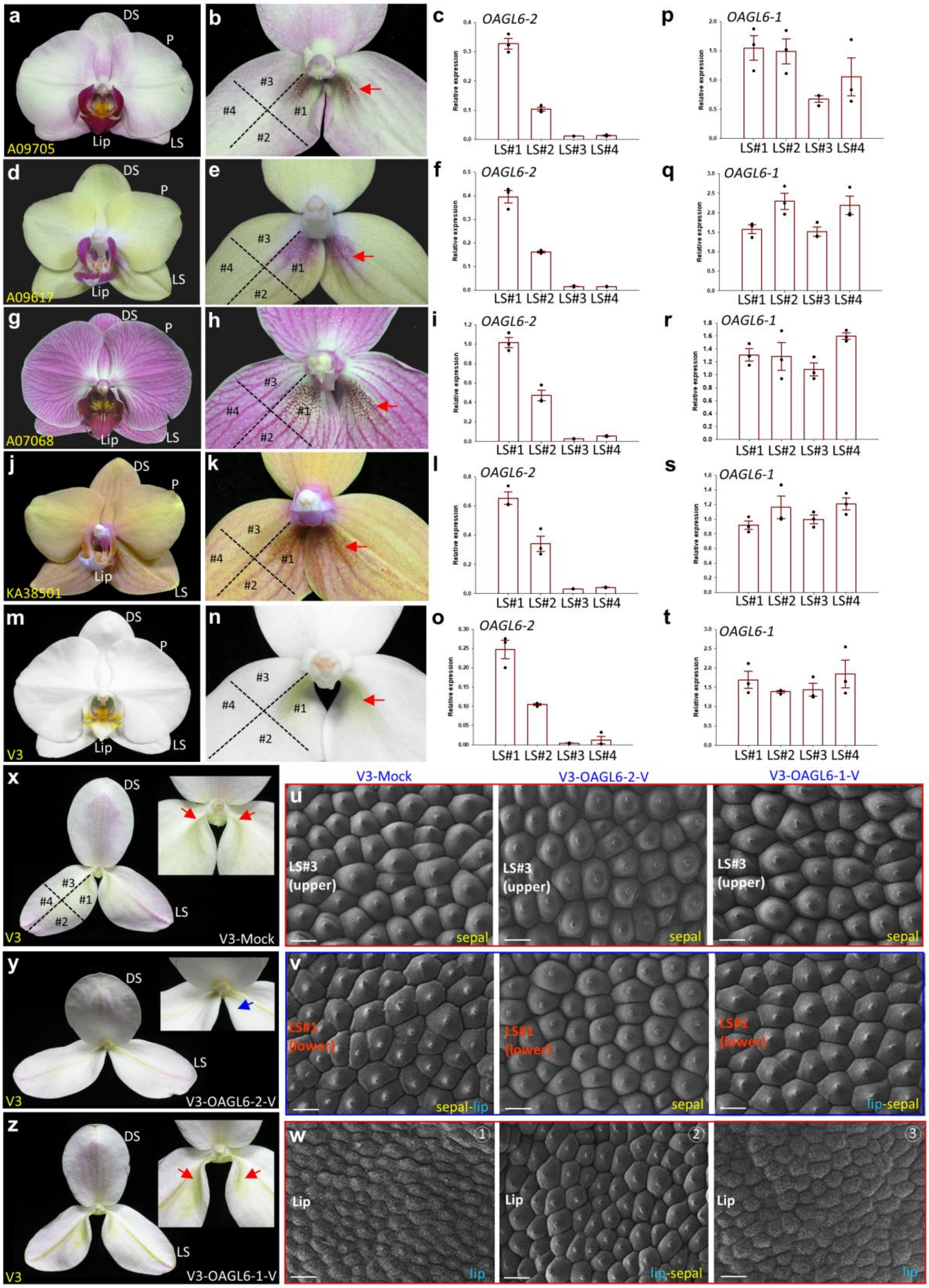

Supplementary Figure 1. The emergence of L' in the lower lateral sepals changed its structure and morphology.

a-t A flower (**a, d, g, j, m**), close-up of the lateral sepals (**b, e, h, k,** n), and the expression pattern of *OAGL6-2* (**c, f, i, l, o**) and *OAGL6-2* (**p-t**) in four parts of lateral sepals (LS #1 to #4) of wild-type *Phalaenopsis* A09705 (**a, b, c, p**), A09617 (**d, e, f, q**), A07068 (**g, h, i, r**), KA38501 (**j, k, l, s**) and V3 (**m, n, o, t**). Lip: lips, P: petals, DS: dorsal sepals, LS: lateral sepals. The red spots in portion #1 in the lower lateral sepals (**b, e, h, k, n**) were indicated as red arrows. In (**c, f, i, l, o, p-t**), error bars show ± SEM. n=3 biologically independent samples.

u The epidermal cells of the #3 portion in the upper lateral sepals of wild-type control (left), *OAGL6-2*-VIGS (middle) and *OAGL6-1*-VIGS (right) V3 flowers exhibit conical cell morphology. Bar = 50 µm.

v The epidermal cells of the #1 portion in the lower lateral sepals of wild-type control (left) and *OAGL6-1*-VIGS (right) flowers exhibit a flattened sepal/lip-like cell morphology, whereas *OAGL6-2*-VIGS (middle) V3 exhibits conical cell morphology. Bar = 50 µm.

w The epidermal cells of the wild-type control (left) and *OAGL6-1*-VIGS (right) lips exhibit a flattened lip-like cell morphology, whereas those of *OAGL6-2*-VIGS (middle) V3 exhibit a conical cell morphology. Bar = 50 µm. In (**u, v, w**), each experiment was repeated twice independently with similar results.

x Back view of the dorsal (DS) and lateral (LS) sepals of wild-type control V3, which show curved edges in the lower portion of the lateral sepals (red arrow).

y Back view of the dorsal (DS) and lateral (LS) sepals of *OAGL6-2*-VIGS V3, which show flattened edges in the lower portion of the lateral sepals (blue arrow).

z Back view of the dorsal (DS) and lateral (LS) sepals of *OAGL6-1*-VIGS V3, which show enhanced curved edges in the lower portion (red arrow).

Source data underlying Supplementary Figure 1c, 1f, 1i, 1l, and 1o-t are provided as a Source Data file.

Supplementary Fig. 2

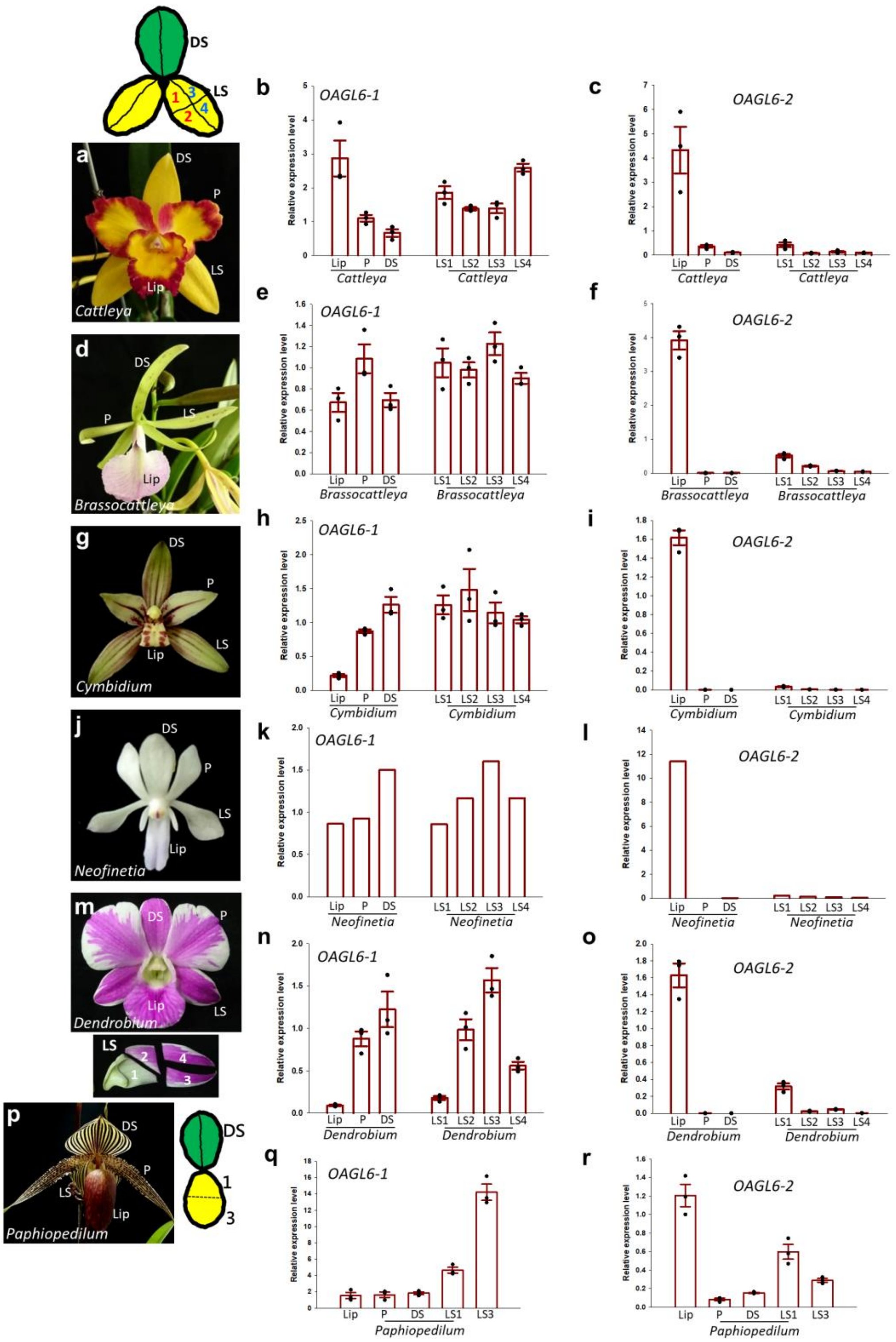

Supplementary Figure 2. The expression of *OAGL6-1* and *OAGL6*-2 in the lips, petals, dorsal sepals and four parts of lateral sepals of various orchid flowers.
a-r A flower (**a, d, g, j, m, p**) and the expression pattern of *OAGL6-1* (**b, e, h, k, n, q**) and *OAGL6-2* (**c, f, i, l, o, r**) in the lips (Lip), petals (P), and dorsal sepals (DS) as well as four parts of lateral sepals (LS #1 to 4) in wild-type *Cattleya* spp. (**a-c**), *Brassocattleya* spp. (**d-f**), *Cymbidium* spp. (**g-i**), *Neofinetia* spp. (**j-l**), *Dendrobium* spp. (**m-o**) and two parts of the lateral sepals (LS #1 and 3) of *Paphiopedilum* spp. (**p-r**) orchids. Lip: lips, P: petals, DS: dorsal sepals, LS: lateral sepals. In (**b, e, h, n, q**, **c, f, i, o, r**), error bars show ± SEM. n=3 biologically independent samples. In (**k, l**), n=1 biologically sample. Source data underlying Supplementary Figure 2b, 2c, 2e, 2f, 2h, 2i, 2k, 2l, 2n, 2o, 2q, and 2r are provided as a Source Data file.

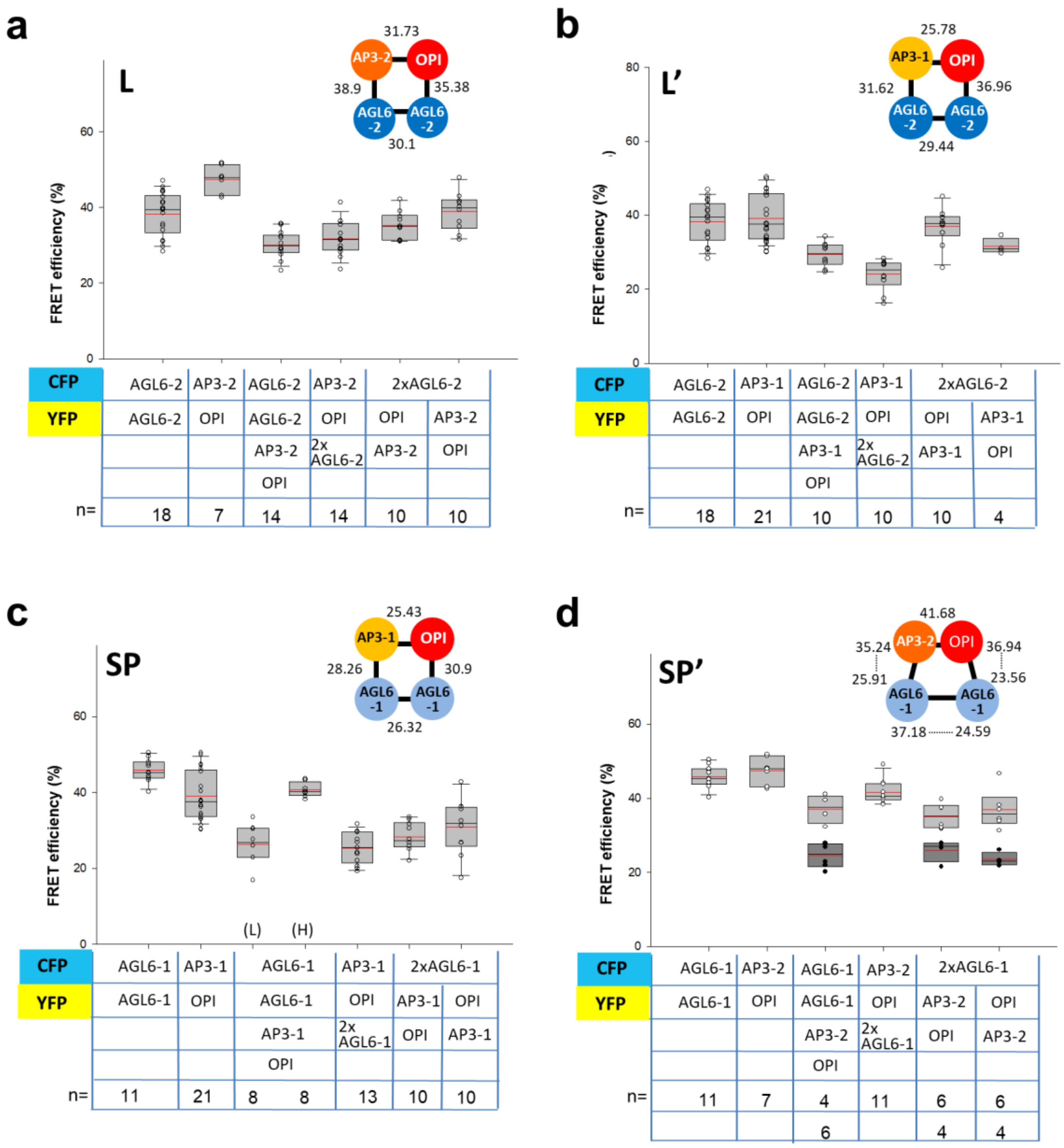

Supplementary Figure 3. FRET analysis of the emergence of L' and SP' complexes in the P code model.

Boxplots of the standardized FRET efficiency of L (OAP3-2/OAGL6-2/OPI) **(a)**, L' (OAP3-1/OAGL6-2/OPI) **(b)**, SP (OAP3-1/OAGL6-1/OPI) **(c)** and SP' (OAP3-2/OAGL6-1/OPI) **(d)** MADS proteins. (Red line: mean). Source data are provided as a Source Data file.

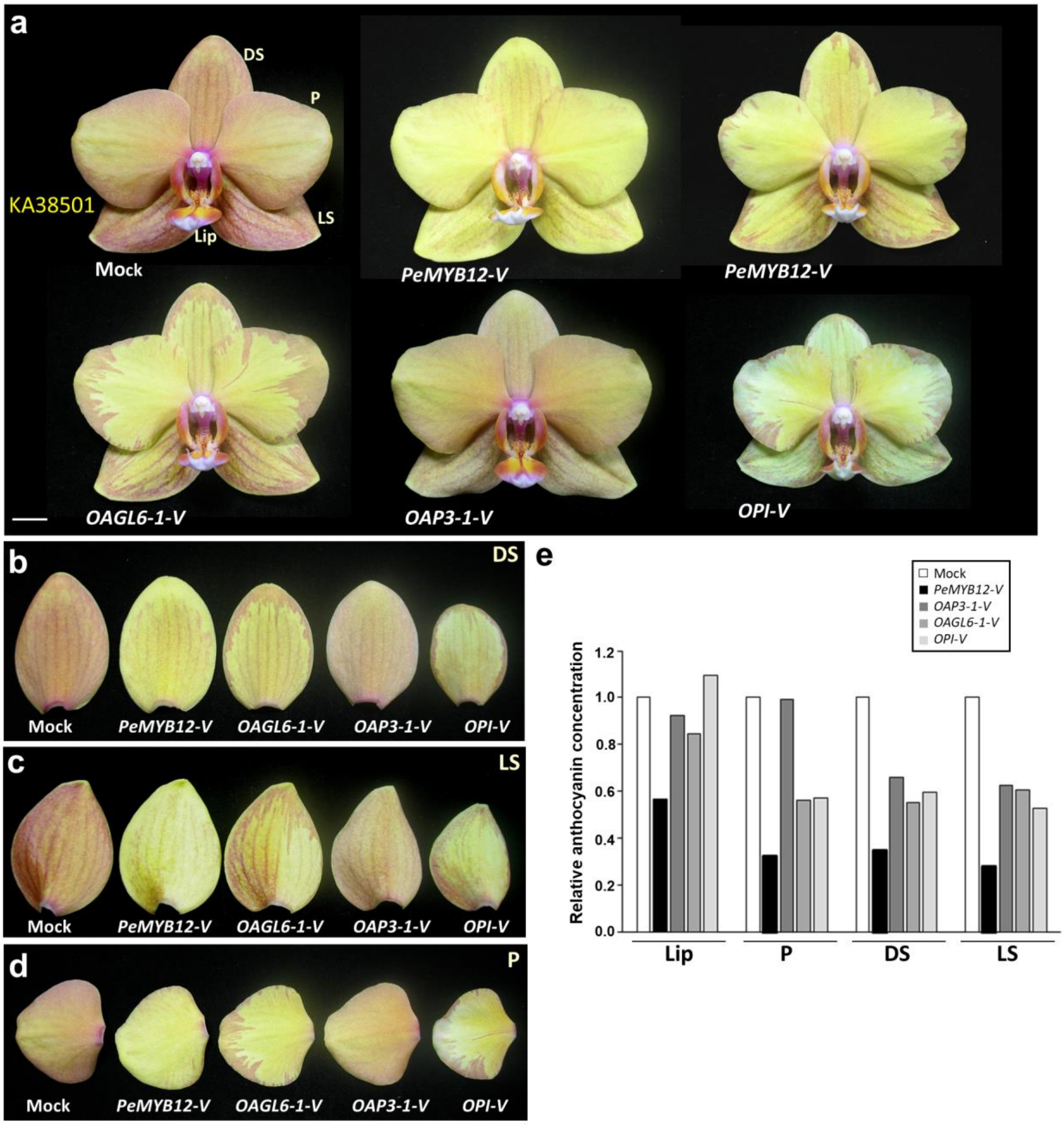

Supplementary Figure 4. Analysis of pigmentation in *PeMYB12/OAGL6-1/OAP3-1/OPI*-VIGS *Phalaenopsis* KA38501 flowers.

a-d Flowers **(a)**, dorsal sepals (DS) **(b)**, lateral sepals (LS) **(c)** and petals (P) **(d)** of wild-type control (Mock), strong and medium *PeMYB12*-VIGS, *OAGL6-1*-VIGS, *OAP3-1*-VIGS and *OPI*-VIGS *Phalaenopsis* KA38501 flowers. Bars = 10 mm.

e The detection of anthocyanin contents in the lips (Lip), petals (P), dorsal sepals (DS) and lateral sepals (LS) of wild-type control (mock), *PeMYB12*-VIGS, *OAP3-1*-VIGS, *OAGL6-1*-VIGS and *OPI*-VIGS flowers. n=1 biologically sample. Source data underlying Supplementary Figure 4e are provided as a Source Data file.

Supplementary Fig. 5

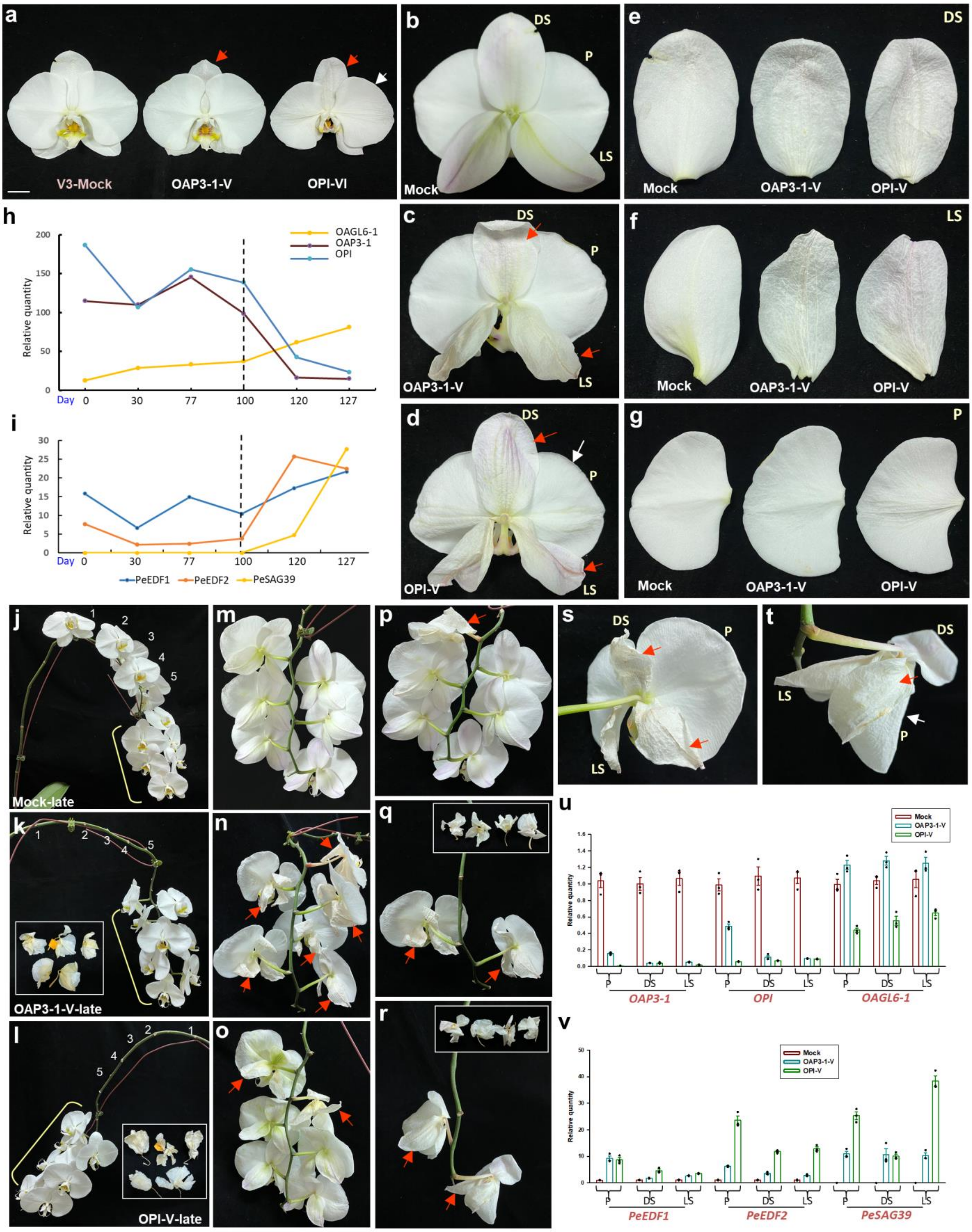

Supplementary Figure 5. Analysis of sepal/petal senescence in *OAP3-1* and *OPI*-VIGS *Phalaenopsis* V3 flowers.

a The front view of a flower of control (left), *OAP3-1*-VIGS (middle) and *OPI*-VIGS (right) *Phalaenopsis* V3. The sepals showed senescence (red arrow) in *OAP3-1*-VIGS (middle) and *OPI*-VIGS (right) flowers, whereas petals showed senescence (white arrow) only in *OPI*-VIGS (right) flowers. Bars = 20 mm.

b-d The back view of a flower of (**a**) control (**b**), *OAP3-1*-VIGS (**c**) and *OPI*-VIGS (**d**) *Phalaenopsis* V3. The dorsal (DS) and lateral (LS) sepals showed senescence (red arrow) in *OAP3-1*-VIGS (**c**) and *OPI*-VIGS (**d**) flowers, whereas the petals showed senescence (white arrow) only in *OPI*-VIGS (**d**) flowers.

e-g The dorsal sepals (DS) **(e)**, lateral sepal (LS) **(f)** and petals (P) (**g**) of control, *OAP3-1*-VIGS and *OPI*-VIGS V3 (from left to right) at the same developmental stage.

h-i Analysis of the expression profile from NGS data for *OAP3-1/OPI/OAGL6-1* **(h)** and *PeSAG39/PeEDF1/2* **(i)** throughout V3 flower development. The dashed lines indicate that the senescence of the V3 flowers starts at 100 days after flower opening. The data was derived from one NGS data.

j-l The flowers of control **(j)**, *OAP3-1*-VIGS-late **(k)** and *OPI*-VIGS-late **(l)** V3 at the same stage. The first five flowers (boxed) were senescent and had abscised from *OAP3-1*-VIGS-late **(k)** and *OPI*-VIGS-late **(l)** V3 inflorescences, whereas five flowers of the control at the same stage did not show signs of senescence and were still attached to the inflorescences **(j).** The newly emerged flowers after VIGS infection are indicated in yellow.

m-o The back view of flowers from (**j, k, l**) of control **(m)**, *OAP3-1*-VIGS-late **(n)** and *OPI*-VIGS-late **(o)** V3, which were grown one more month past those shown in (**j, k, l**). The sepals from *OAP3-1*-VIGS-late **(n)** and *OPI*-VIGS-late **(o)** V3 flowers were senescent (red arrow), whereas flowers of the control (mock) **(m)** did not show signs of senescence.

p-r The back view of flowers from (**m, n, o**) of control **(p)**, *OAP3-1*-VIGS-late **(q)** and *OPI*-VIGS-late **(r)** V3, which were grown 10 more days past those of (**m, n, o**). Four flowers (boxed) were already senescent and had abscised, whereas the remaining two flowers from *OAP3-1*-VIGS-late **(q)** and *OPI*-VIGS-late **(r)** V3 flowers also showed signs of senescence (red arrow). Only one flower showed signs of senescence (red arrow) in the control (mock) **(p)** at the same stage.

s-t The back view of the last flower from (**q, r**) of *OAP3-1*-VIGS (**s**) and *OPI*-VIGS (**t**) *Phalaenopsis* V3. The dorsal (DS) and lateral (LS) sepals were completely senescent (red arrow) in the *OAP3-1*-VIGS (**s**) and *OPI*-VIGS (**t**) flowers, whereas the petals were senescent (white arrow) in *OPI*-VIGS (**t**) and showed signs of senescence in *OAP3-1*-VIGS (**s**) flowers.

u-v Analysis of the expression of *OAP3-1/OPI/OAGL6-1* **(u)** and *PeSAG39/PeEDF1/2* **(v)** in the petals (P), dorsal sepals (DS) and lateral sepals (LS) of control (mock), *OAP3-1*-VIGS-late and *OPI*-VIGS-late V3 flowers. Error bars show ± SEM. n=3 biologically independent samples. Source data underlying Supplementary Figure 5h, 5i, 5u, and 5v are provided as a Source Data file.

Supplementary Fig. 6

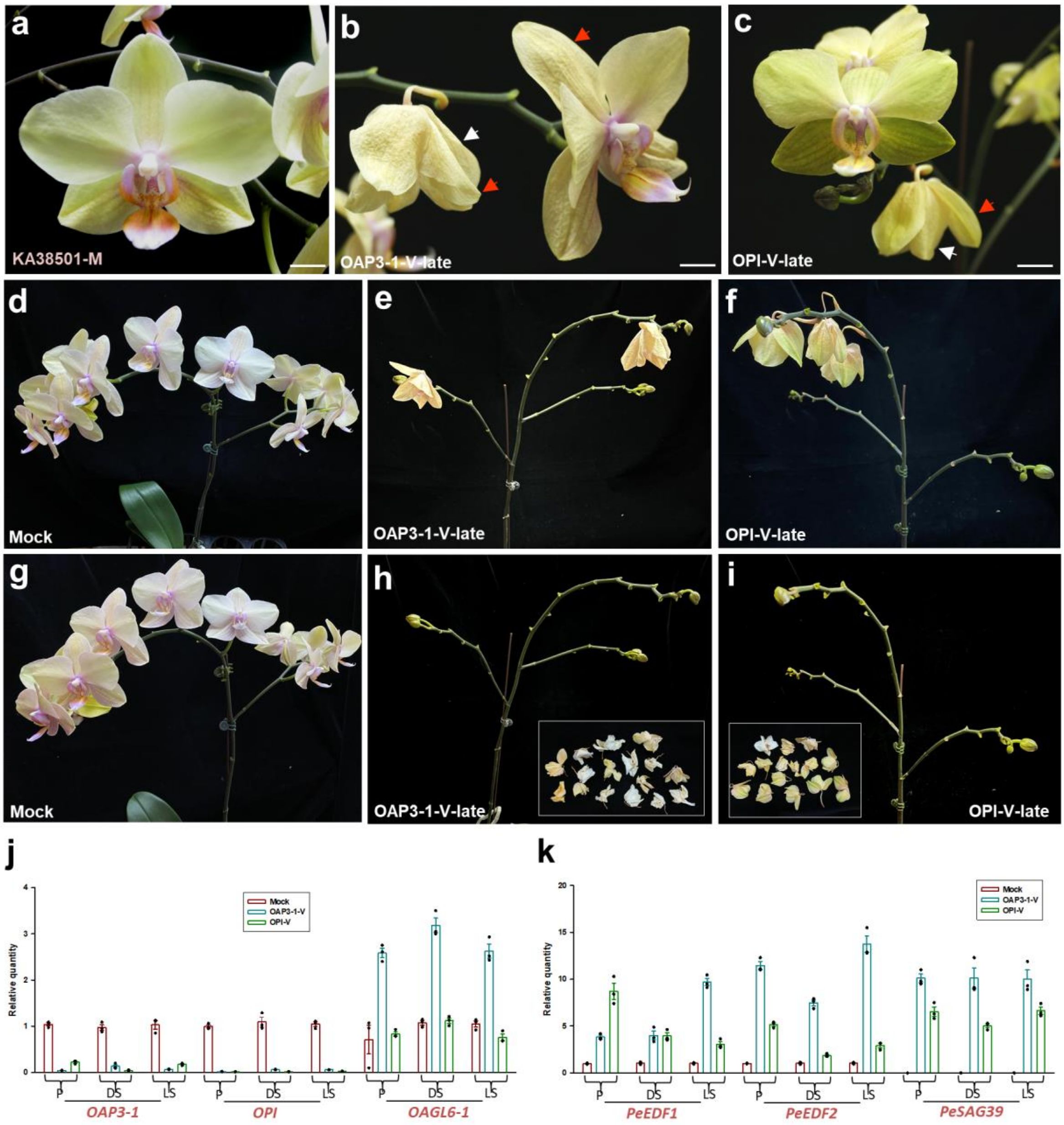

Supplementary Figure 6. Analysis of sepal/petal senescence in *OAP3-1* and *OPI*-VIGS *Phalaenopsis* KA38501 flowers.

a-c The front view a flower of control (**a**), *OAP3-1*-VIGS-late (**b**) and *OPI*-VIGS-late (**c**) *Phalaenopsis* KA38501. The sepals (red arrow) and petals (white arrow) showed senescence in *OAP3-1*-VIGS (**b**) and *OPI*-VIGS (**c**) senescent flowers. Bars = 10 mm. **d-f** At the same stage, all flowers were senescent from *OAP3-1*-VIGS-late (**e**) and *OPI*-VIGS-late (**f**) *Phalaenopsis* KA38501, whereas the flowers of the control (mock) inflorescence showed no sign of senescence (**d**).

g-i Later, from (**d-f**), all the flowers were senescent and had abscised (boxed) from the *OAP3-1*-VIGS-late (**h**) and *OPI*-VIGS-late (**i**) inflorescences, whereas the flowers of the control inflorescence still showed no sign of senescence or abscission (**g**).

j-k Analysis of the expression of *OAP3-1/OPI/OAGL6-1* **(j)** and *PeSAG39/PeEDF1/2* **(k)** in the petals (P), dorsal sepals (DS) and lateral sepals (LS) of control (mock), *OAP3-1*-VIGS-late and *OPI*-VIGS-late F894 flowers. Error bars show ± SEM. n=3 biologically independent samples. Source data underlying Supplementary Figure 6j and 6k are provided as a Source Data file.

Supplementary Fig. 7

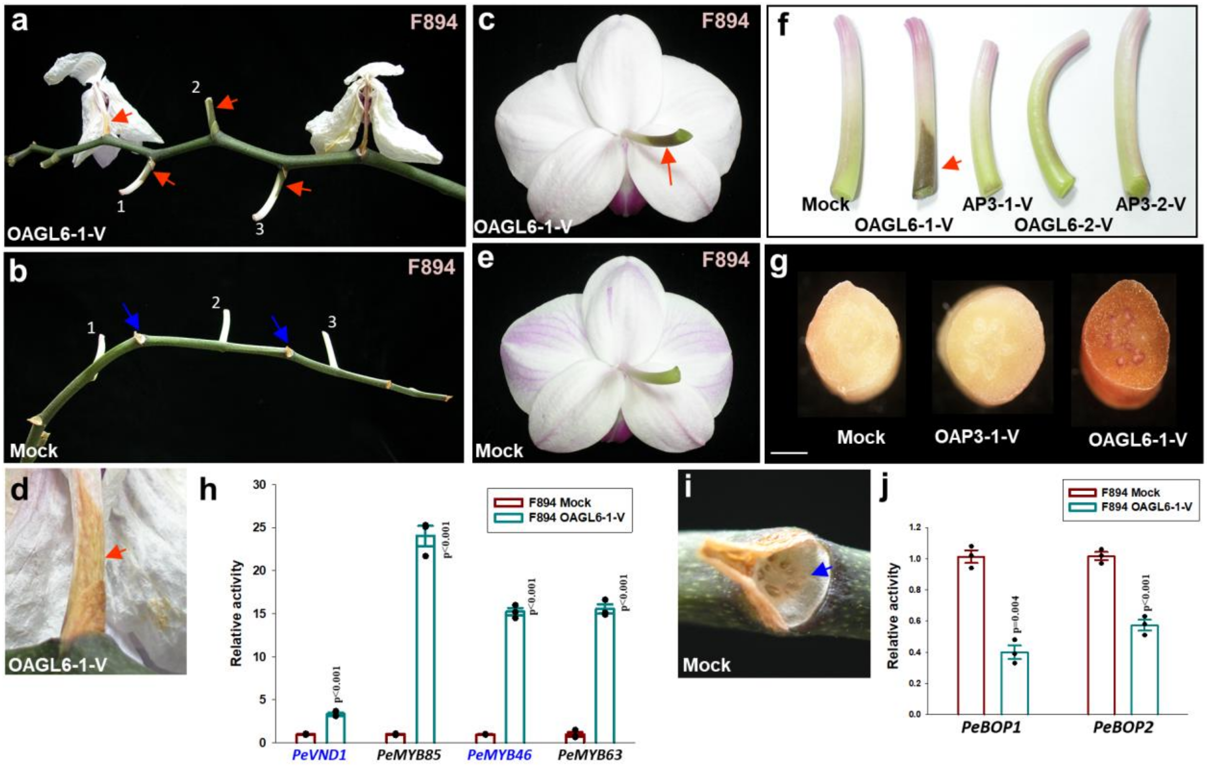

Supplementary Figure 7. Analysis of the abscission of pedicels in *OAGL6-1* VIGS *Phalaenopsis* F894 flowers.

a-b The flowers were senescent and had abscised completely from the control (mock) inflorescence **(b,** blue arrow**),** whereas senescent flowers had not abscised from the *OAGL6-1*-VIGS inflorescence **(a)** of *Phalaenopsis* F894. The three pedicels (#1, 2, and 3) remaining in (**a, b**) were manually detached from the flowers used for other experiments. The red arrow indicates the dark brown color in the pedicel of *OAGL6-1*-VIGS F894 flowers.

c-f The back view of flowers of control (mock) **(e)** and *OAGL6-1*-VIGS **(c)** *Phalaenopsis* F894. A dark brown color was observed in the pedicel of *OAGL6-1*-VIGS (red arrow in **c, d, f**) but was absent in the control **(e, f)** and *OAP3-1*, *OAGL6-2*, and *OAP3-2* VIGS **(f)** pedicels. (**d**) is a close-up a pedicel from a non-abscised flower in (**a**).

g The pedicel of control (mock), *OAP3-1*-VIGS and *OAGL6-1*-VIGS V3 (from left to right) flowers after staining with phloroglucinol. Bars = 1 mm.

h Analysis of the expression of *PeVND1*, *PeMYB85*, *PeMYB46* and *PeMYB63* in the pedicel of control (mock) and *OAGL6-1*-VIGS F894 flowers.

i Close-up of the AZ in a control (mock) abscised pedicel from **(b)**.

j Analysis of the expression of *PeBOP1* and *PeBOP2* in the pedicel AZ of control and *OAGL6-1*-VIGS F894 flowers. In (**h,j**), error bars show ± SEM. n=3 biologically independent samples. The *p* value indicates significant differences from the control (Mock) value. Statistical test used was two-sided according to Student's *t*-test. Source data underlying Supplementary Figure 7h and 7j are provided as a Source Data file.

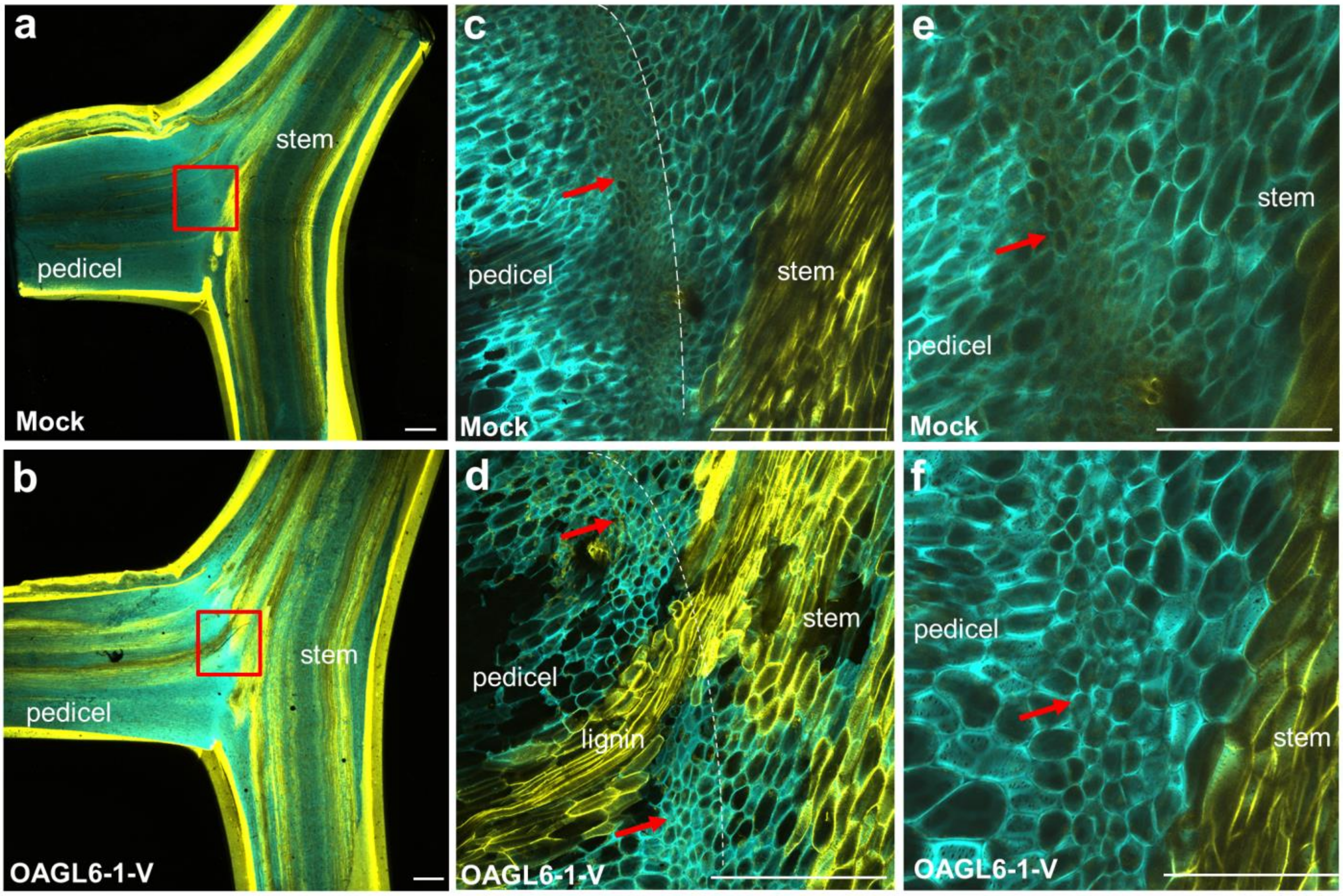

Supplementary Figure 8. Analysis of the lignin formation and the AZ cell formation in pedicels of *OAGL6-1* VIGS *Phalaenopsis* V3 flowers.

a-b Longitudinal views of the pedicels and the connected stems of the control (Mock) **(a)** and *OAGL6-1*-VIGS V3 **(b)** flowers after staining with Calcofluor White for cellulose (in cyan) and Auramine O for lignin and cuticle (in yellow). Bars = 500 µm.

c-d Close-up images of the junctions (dashed line) of the pedicels and stems of the control (Mock) **(c)** and *OAGL6-1*-VIGS V3 **(d)** from the boxed regions in **(a-b)**. The arrows indicate the AZ region with the formation of small differentiated cells. The cells in the AZ region in **(d)** were altered by the penetration of lignin from the pedicel to the stem. Bars = 500 µm.

e-f Close-up images of the AZ region (arrowed) in the junctions for the pedicel and stem of the control (Mock) **(e)** and *OAGL6-1*-VIGS V3 **(f)**. Compared to that in **(e)**, the formation of the cells in the AZ region in **(f)** was clearly altered. Bars = 250 µm. In (**a-f**), each experiment was repeated twice independently with similar results.

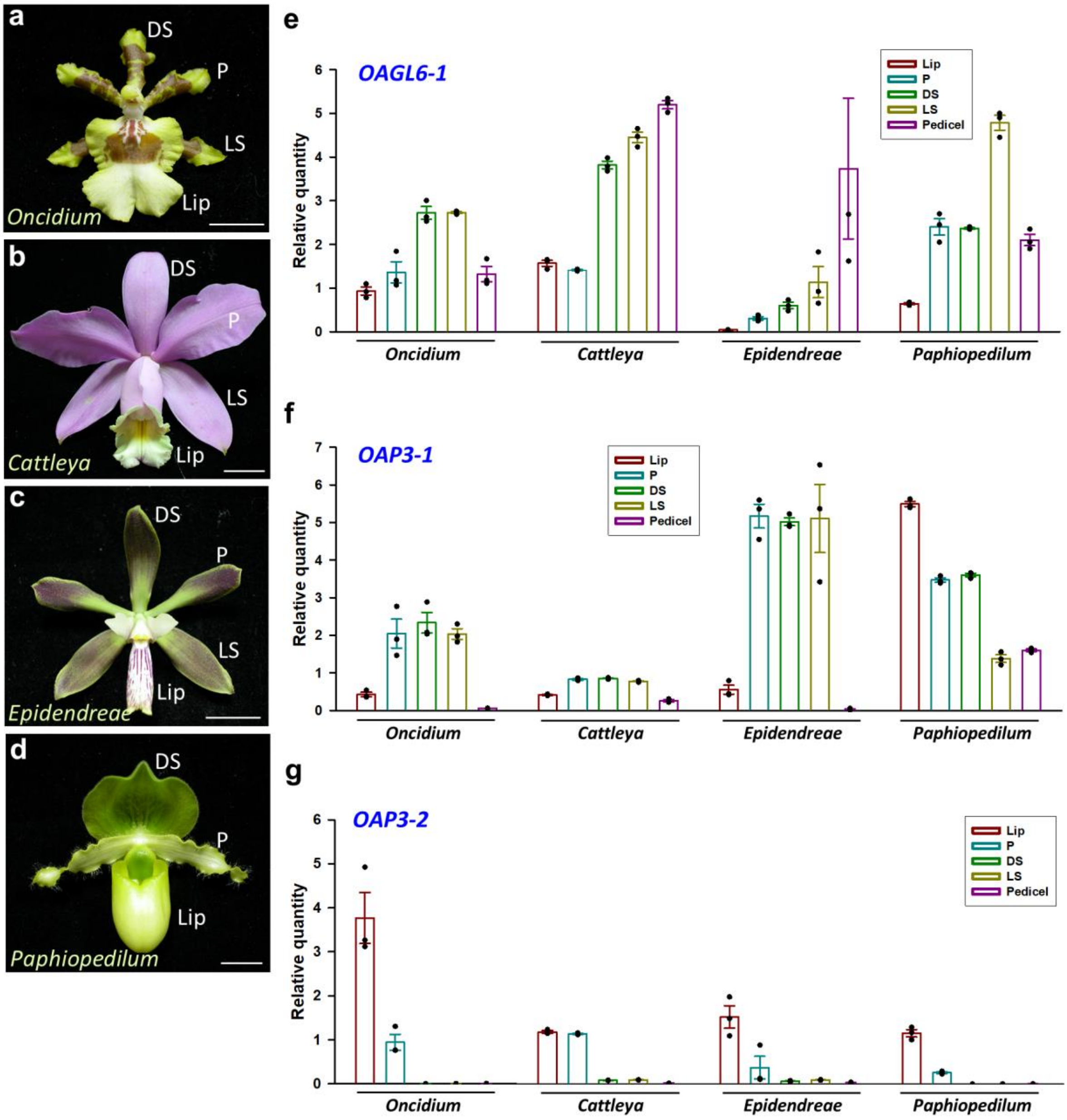

Supplementary Figure 9. Analysis of the expression of *OAGL6-1*, *OAP3-1* and *OAP3*-2 in the perianth and pedicel of various orchid flowers.

A flower (**a-d**) and the expression pattern of *OAGL6-1* (**e**), *OAP3-1* (**f**) and *OAP3-2* (**g**) in the lips (Lip), petals (P), dorsal sepals (DS) and lateral sepals (LS) of wild-type *Oncidium* spp. (**a**), *Cattleya* spp. (**b**), *Epidendreae* spp. (**c**) and *Paphiopedilum* spp. (**d**) orchids. Bars = 10 mm. In (**e,f, g**), error bars show ± SEM. n=3 biologically independent samples. Source data underlying Supplementary Figure 9e-g are provided as a Source Data file.

Supplementary Fig. 10

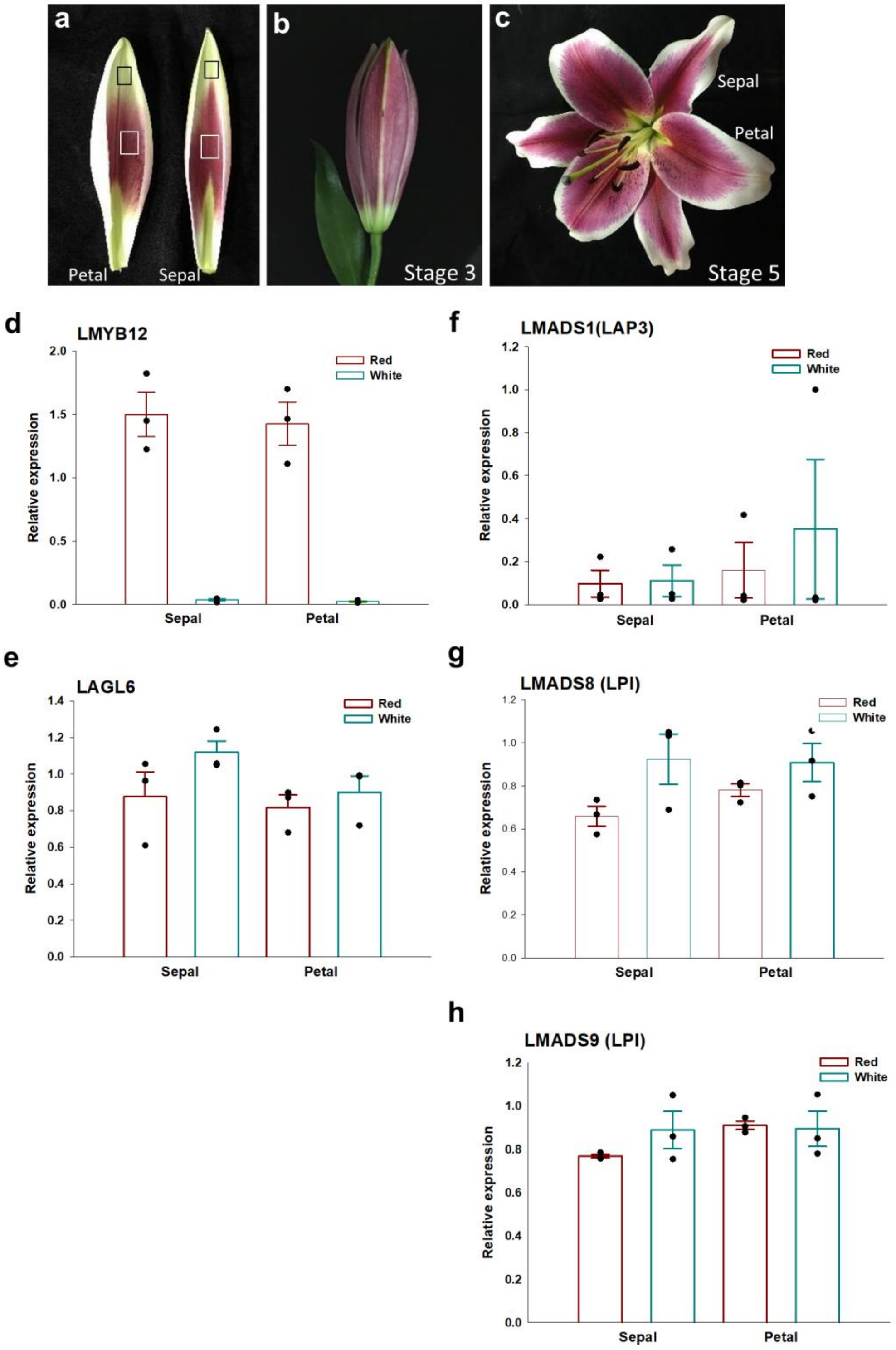

Supplementary Figure 10. Analysis of the expression of *LMYB12*, *LAGL6*, and *LMADS1/8/9* in the sepals and petals of lily flowers.

a-c Stage 3 (**b**) and stage 5 (**c**) flowers described by Suzuki *et al.*[1] for Asiatic hybrid lilies (*Lilium* spp.). The sepals/petals from stage 3 flowers (**a**) in which the red pigmentation occurred in the center portion. The center red portion (white box) and the upper colorless portion (black box) of the sepals/petals were used for the experiments to detect gene expression[1].

d-h Analysis of the expression of *LMYB12* (**d**), *LAGL6* (**e**), *LMADS1/LAP3* (**f**), *LMADS8/LPI* (**g**) and *LMADS9/LPI* (**h**) in the center red (Red) and upper colorless (White) portions of the sepals/petals from stage 3 Asiatic hybrid lily (*Lilium* spp.) flowers. The results indicated that *LMYB12* expression was significantly higher in the center red (Red) portion than in the upper colorless (White) portion **(d)**. By contrast, *LAGL6* (**e**), *LMADS1/LAP3* (**f**), *LMADS8/LPI* (**g**) and *LMADS9/LPI* (**h**) were expressed similarly or even higher in the upper colorless (White) portion than in the center red (Red) portion. Error bars show ± SEM. n=3 biologically independent samples. Source data underlying Supplementary Figure 10d-h are provided as a Source Data file.

Supplementary Fig. 11

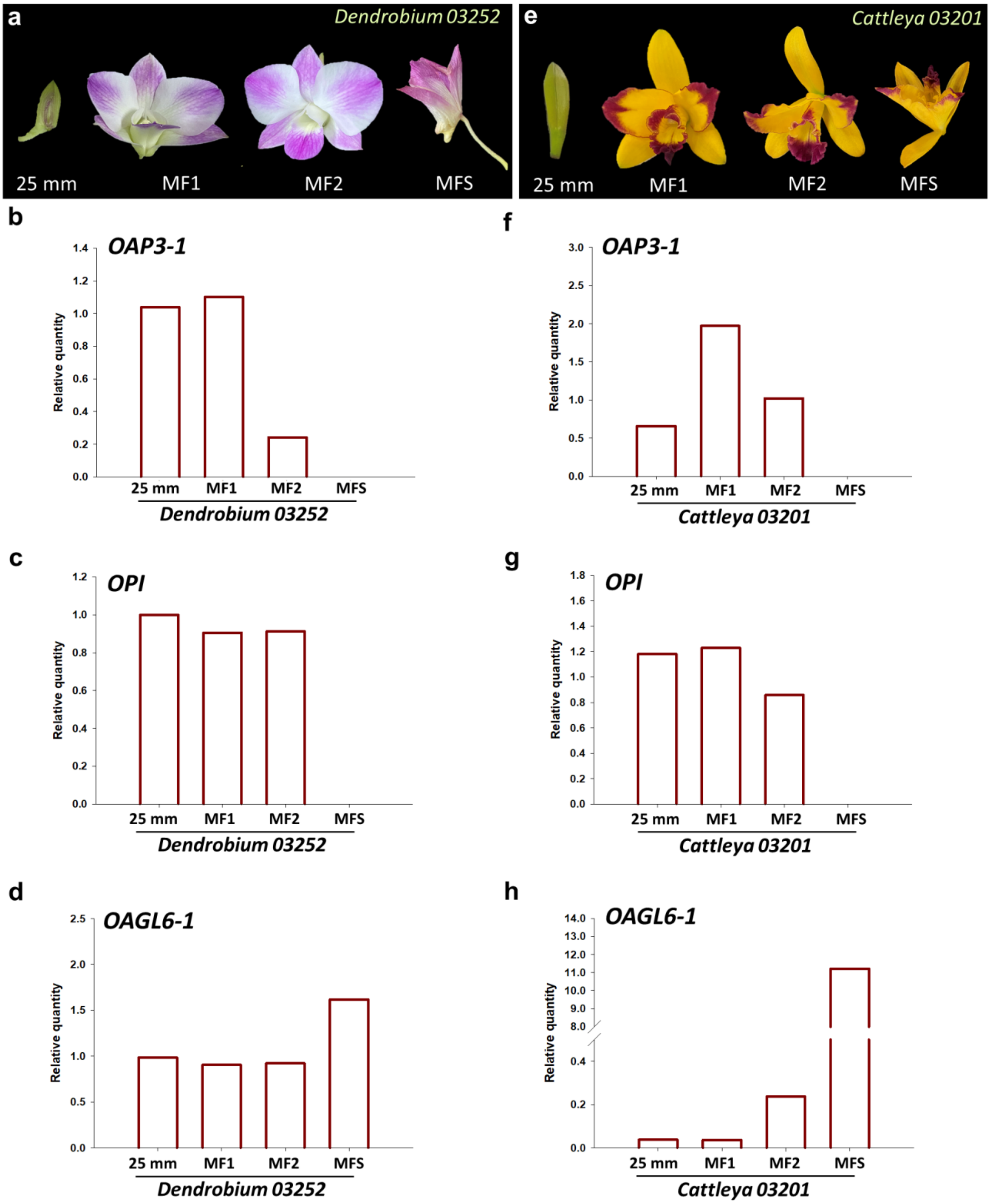

Supplementary Figure 11. Analysis of the expression of *OAP3-1, OPI* and *OAGL6-1* in the perianth during different developmental stages of various orchid flowers.

A flower (**a, e**) and the expression pattern of *OAP3-1* (**b, f**), *OPI* (**c, g**) and *OAGL6-1* (**d, h**) at different developmental stages of flowers, 25 mm buds (25 mm), mature flowers 1 and 2 (MF1 and MF2) and mature senescent flowers (MFS) of wild-type *Dendrobium* 03252 (**a-d**) and *Cattleya* 03201 (**e-h**) orchids. The results indicated that *OAP3-1* and *OPI* expression was downregulated and reached the lowest level at the stage when the flowers were senescent. In contrast, the expression of *OAGL6-1* significantly increased in the senescent flowers. In (**b-d, f-h**), n=1 biologically sample. Source data underlying Supplementary Figure 11b-d and 11f-h are provided as a Source Data file.

Supplementary Fig. 12

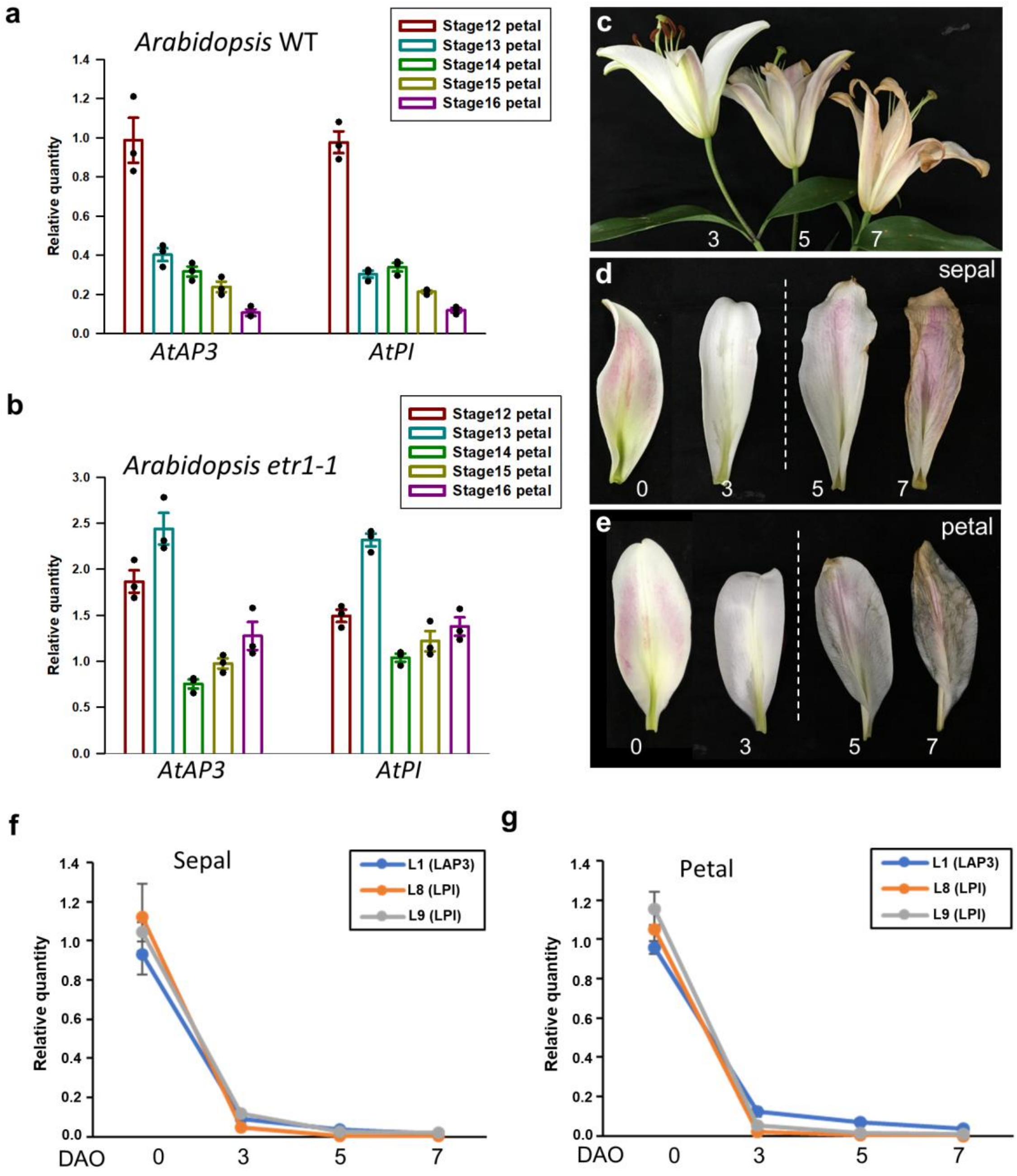

Supplementary Figure 12. Analysis of the expression of *AP3* and *PI* orthologs during flower development and senescence in *Arabidopsis* and lily.

a-b Detection of the expression of *Arabidopsis AtAP3* and *AtPI* in wild-type (**a**) and *etr1-1* mutant (**b**) flowers at 5 different developmental stages (12 to 16). The results indicated that the expression of both *AtAP3* and *AtPI* was significantly downregulated throughout flower development and that it reached its lowest levels at stage 16 when the flowers were senescent **(a)**. In contrast, in the *etr1-1* mutant, in which the senescence of the flower was delayed, the expression of *AtAP3* was decreased; however, it remained at a certain high level even in stage 16, whereas *AtPI* expression remained at the same level from stage 12 to 16 throughout flower development **(b)**. Error bars show ± SEM. n=3 biologically independent samples.

c-e Lily flowers (**c**), sepals (**d**) and petals (**e**) at 0, 3, 5 and 7 days after flower opening. The sepals and petals showed clear signs of senescence at 3-5 days after flower opening and were completely senescent at 7 days after flower opening.

f-g Analysis of the expression of *LMADS1/LAP3*, *LMADS8/LPI* and *LMADS9/LPI* in sepals (**f**) and petals (**g**) at 0, 3, 5 and 7 days after flower opening. The results indicated that the expression of *LMADS1/LAP3*, *LMADS8/LPI* and *LMADS9/LPI* was significantly downregulated at 3 days after flower opening and remained at the same low level when the flowers were senescent. Error bars show ± SEM. n=3 biologically independent samples. Source data underlying Supplementary Figure 12a, 12b, 12f, and 12g are provided as a Source Data file.

Supplementary Fig. 13

a

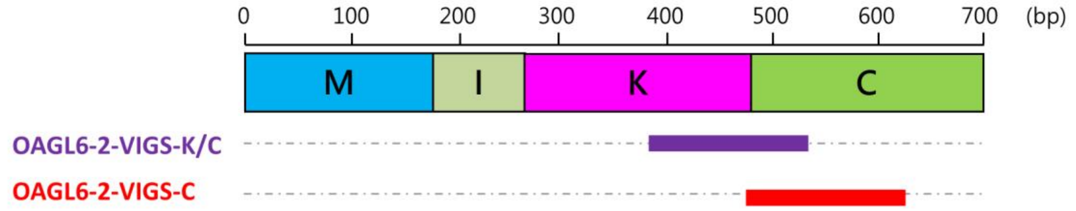

b

Genes	Primer name	Sequence (sequences in bold letters represent recombination sites)
OAGL6-2 (K+C)	PeM9-VIGS-KF	5'- **GGGGACAAGTTTGTACAAAAAAGCAGGCTCA**TTGAATCATCTCTATCTCAAGCCAGAC -3'
	PeM9-VIGS-KR	5'- **GGGGACCACTTTGTACAAGAAAGCTGGGTC**AGAGCTCTCATCGATCCACCATCTGCC -3'
OAGL6-2 (C)	PeM9-VIGS-F	5'- **GGGGACAAGTTTGTACAAAAAAGCAGGCTCA**GGTGATATTAACAAGCAGCTTAAACA -3'
	PeM9-VIGS-R	5'- **GGGGACCACTTTGTACAAGAAAGCTGGGTC**TTTGAAGAGTGGGTTCTGTATCCATG -3'

c

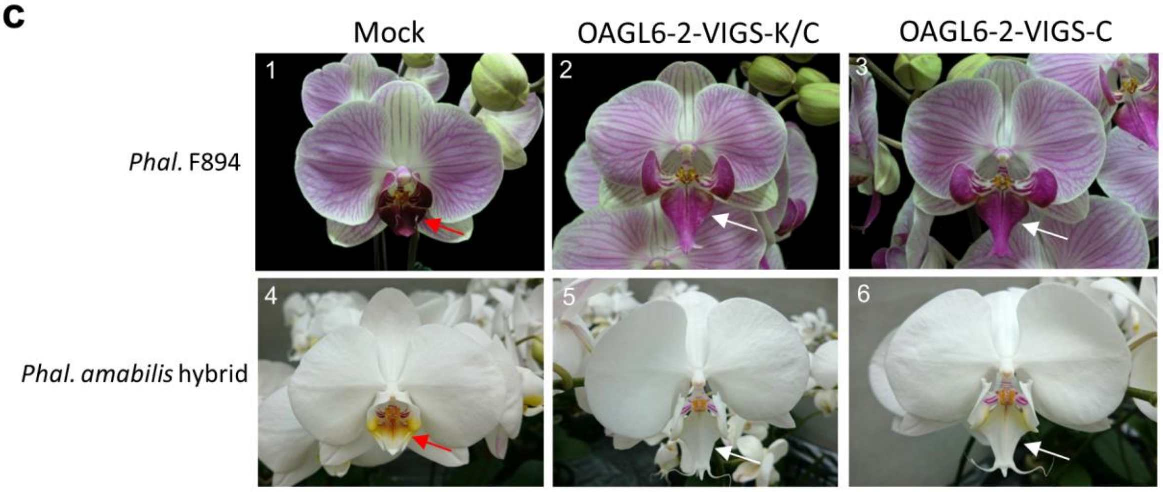

Supplementary Figure 13. The analysis of the two DNA fragments used for the *OAGL6-2* VIGS experiment.

a Schematic diagram of the two DNA fragments (one in the K/C domain, one in the C domain) from *OAGL6-2* used for VIGS experiments for *OAGL6-2*-VIGS-K/C and *OAGL6-2*-VIGS-C.

b Primers used to amplify two DNA fragments in **(a).**

c Similar big lips phenotypes (lips converted into sepal/petal) (white arrows) were observed in both *OAGL6-2*-VIGS-K/C and *OAGL6-2*-VIGS-C flowers for *Phalaenopsis* F894 (-2, -3) and *Phalaenopsis amabilis* hybrid (-5, -6). The mock controls produced normal small lips (red arrows in -1, -4).

Supplementary Fig. 14

a

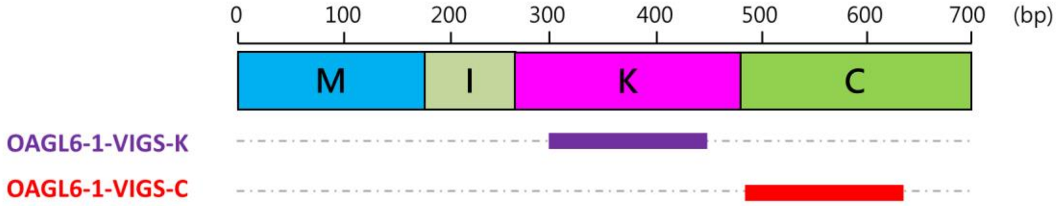

b

Genes	Primer name	Sequence (sequences in bold letters represent recombination sites)
OAGL6-1 (K)	PeM10-VIGS-KF	5'- **GGGGACAAGTTTGTACAAAAAAGCAGGCTCA**ATTACAACGCTCCCACAGGAATTTACTTG -3'
	PeM10-VIGS-KR	5'- **GGGGACCACTTTGTACAAGAAAGCTGGGTC**ACGGAGCTCCTCCATTTGATCCAGCATTAT -3'
OAGL6-1 (C)	PeM10-VIGS-F	5'- **GGGGACAAGTTTGTACAAAAAAGCAGGCTCA**ATAAGCAGCTAAAAATGAAGCTTGAG -3'
	PeM10-VIGS-R	5'- **GGGGACCACTTTGTACAAGAAAGCTGGGTC**GTAGAGTCGGCTCGCATTCCAATGAA -3'

c

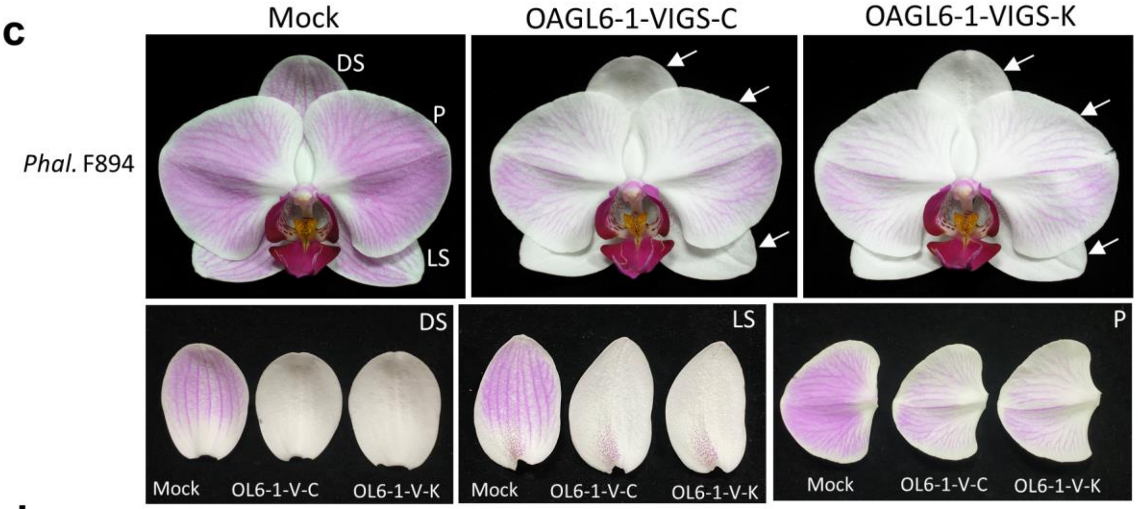

d

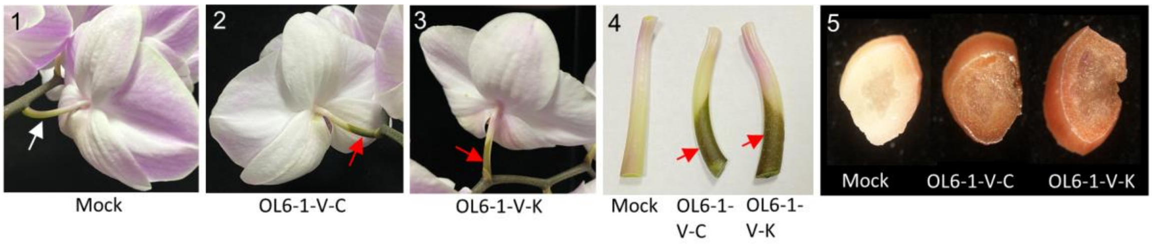

Supplementary Figure 14. The analysis of the two DNA fragments used for the *OAGL6-1* VIGS experiment.

a Schematic diagram of the two DNA fragments (one in the K domain, one in the C domain) from *OAGL6-1* used for VIGS experiments for *OAGL6-1*-VIGS-K and *OAGL6-1*-VIGS-C.

b Primers used to amplify two DNA fragments in **(a).**

c Similar reduced pigmentation phenotypes in dorsal sepal (DS), lateral sepal (LS) and petal (P) (arrows) were observed in both *OAGL6-1*-VIGS-C and *OAGL6-1*-VIGS-K flowers for *Phalaenopsis* F894.

d Similar increased dark color (red arrows) (left 1 to left 4) and lignin formation (left 5) phenotypes in pedicels were observed in both *OAGL6-1*-VIGS-C and *OAGL6-1*-VIGS-K flowers for *Phalaenopsis* F894.

Supplementary Fig. 15

a

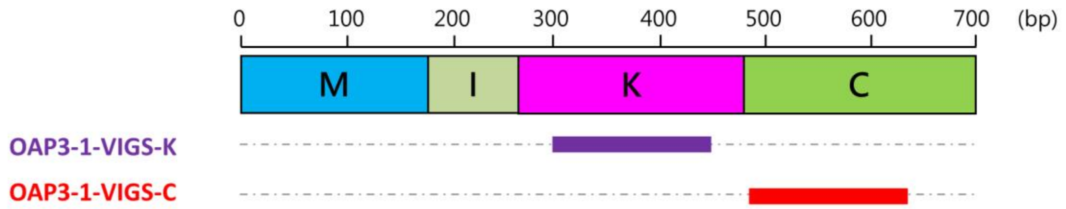

b

Genes	Primer name	Sequence (sequences in bold letters represent recombination sites)
OAP3-1 (K)	PeM2-VIGS-KF	5'- **GGGGACAAGTTTGTACAAAAAAGCAGGCTCA**TATGGAATGCTCAGTATGAGAGGATGCAG -3'
	PeM2-VIGS-KR	5'- **GGGGACCACTTTGTACAAGAAAGCTGGGTC**TCCAAAGTTTGCTCAAGACCGCGCAGTTGC -3'
OAP3-1 (C)	PeM2-VIGS-F	5'- **GGGGACAAGTTTGTACAAAAAAGCAGGCTCA**AGGGAAACTTACCGCGCTCTAATAC -3'
	PeM2-VIGS-R	5'- **GGGGACCACTTTGTACAAGAAAGCTGGGTC**TGAACAACCCTAAAGGAAAACATCT -3'

c

Phal. F894

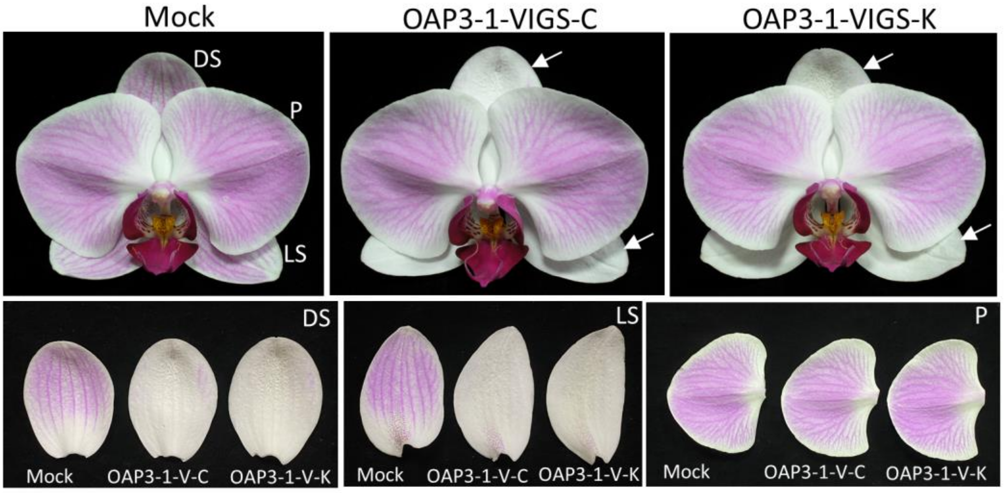

d

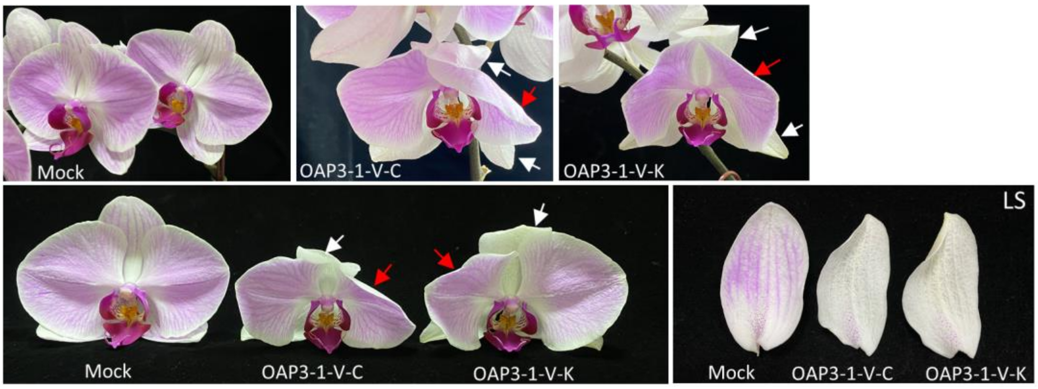

Supplementary Figure 15. The analysis of the two DNA fragments used for the *OAP3-1* VIGS experiment.

a Schematic diagram of the two DNA fragments (one in the K domain, one in the C domain) from *OAP3-1* used for VIGS experiments for *OAP3-1*-VIGS-K and *OAP3-1*-VIGS-C.

b Primers used to amplify two DNA fragments in **(a).**

c Similar reduced pigmentation phenotypes in dorsal sepal (DS) and lateral sepal (LS) (arrows) were observed in both *OAP3-1*-VIGS-C and *OAP3-1*-VIGS-K flowers for *Phalaenopsis* F894.

d Similar early senescence phenotypes in sepals (white arrows) and petals (red arrows) were observed in both *OAP3-1*-VIGS-C and *OAP3-1*-VIGS-K flowers for *Phalaenopsis* F894.

a

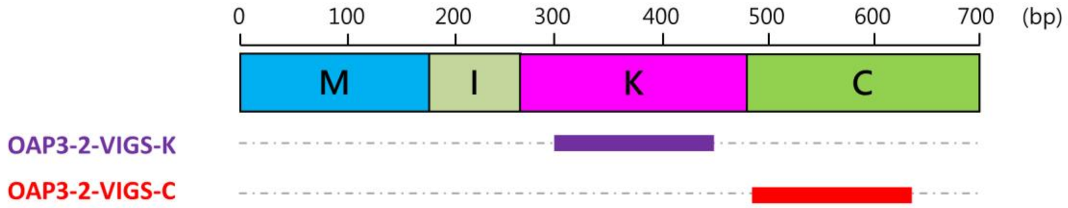

b

Genes	Primer name	Sequence (sequences in bold letters represent recombination sites)
OAP3-2 (K)	PeM3-VIGS-KF	5'- **GGGGACAAGTTTGTACAAAAAAGCAGGCTCA**ATAAGGCAGAGGATGGGCGAGGATCTTGA -3'
	PeM3-VIGS-KR	5'- **GGGGACCACTTTGTACAAGAAAGCTGGGTC**CAACTTTTTTTTGAATGTATCTGTCTGGGT -3'
OAP3-2 (C)	PeM3-VIGS-F	5'- **GGGGACAAGTTTGTACAAAAAAGCAGGCTCA**AGAAACCCACAGGAACTTACTC -3'
	PeM3-VIGS-R	5'- **GGGGACCACTTTGTACAAGAAAGCTGGGTC**GTTCGGCTGGCTTGGTTGGGTACGA -3'

c

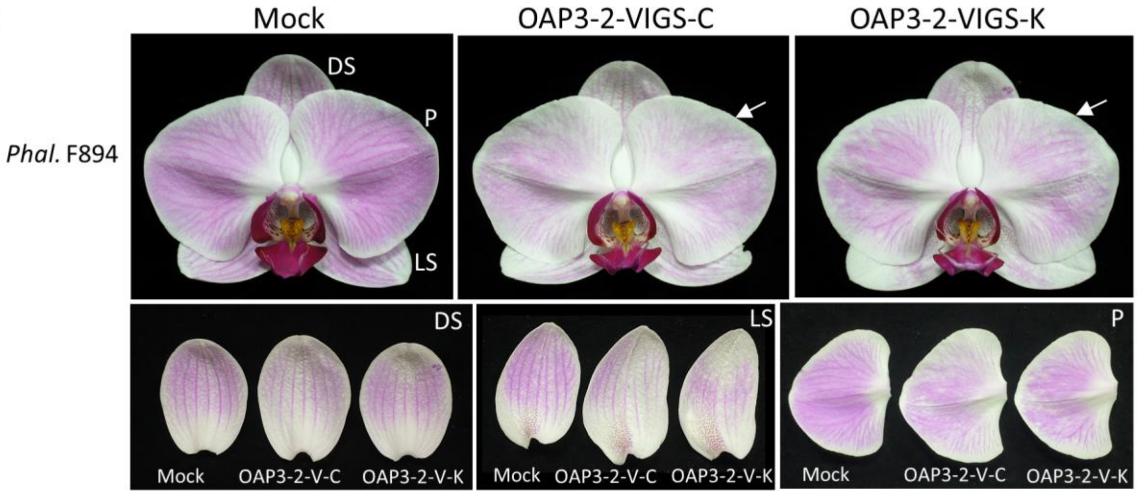

Supplementary Figure 16. The analysis of the two DNA fragments used for the *OAP3-2* VIGS experiment.

a Schematic diagram of the two DNA fragments (one in the K domain, one in the C domain) from *OAP3-2* used for VIGS experiments for *OAP3-2*-VIGS-K and *OAP3-2*-VIGS-C.

b Primers used to amplify two DNA fragments in **(a).**

c Similar reduced pigmentation phenotypes in petal (P) (arrows) were observed in both *OAP3-2*-VIGS-C and *OAP3-2*-VIGS-K flowers for *Phalaenopsis* F894.

Supplementary Fig. 17

a

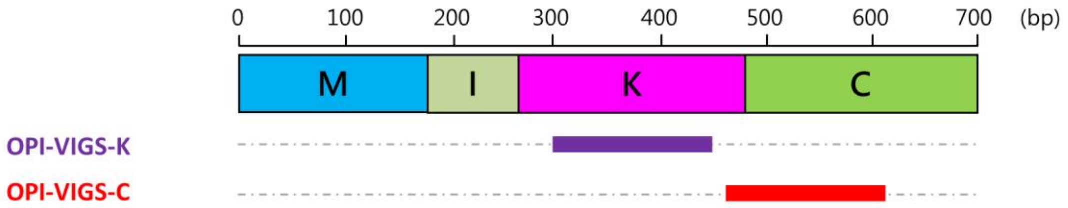

b

Genes	Primer name	Sequence (sequences in bold letters represent recombination sites)
OPI (K)	PeM6-VIGS-KF	5'- **GGGGACAAGTTTGTACAAAAAAGCAGGCTCA**GATTGATCGTATCAAGAAGGAAAATGATA -3'
	PeM6-VIGS-KR	5'- **GGGGACCACTTTGTACAAGAAAGCTGGGTC**ATTTGTTTATCCCGAACGCTGGTGAGACCA -3'
OPI (C)	PeM63-VIGS-F	5'- **GGGGACAAGTTTGTACAAAAAAGCAGGCT**AATGGACTACTTGAAGATGCTAAAAAAGA -3'
	PeM63-VIGS-R	5'- **GGGGACCACTTTGTACAAGAAAGCTGGGTC**GCATACTCGCGATCTTTATGATGATAGCC -3'

c

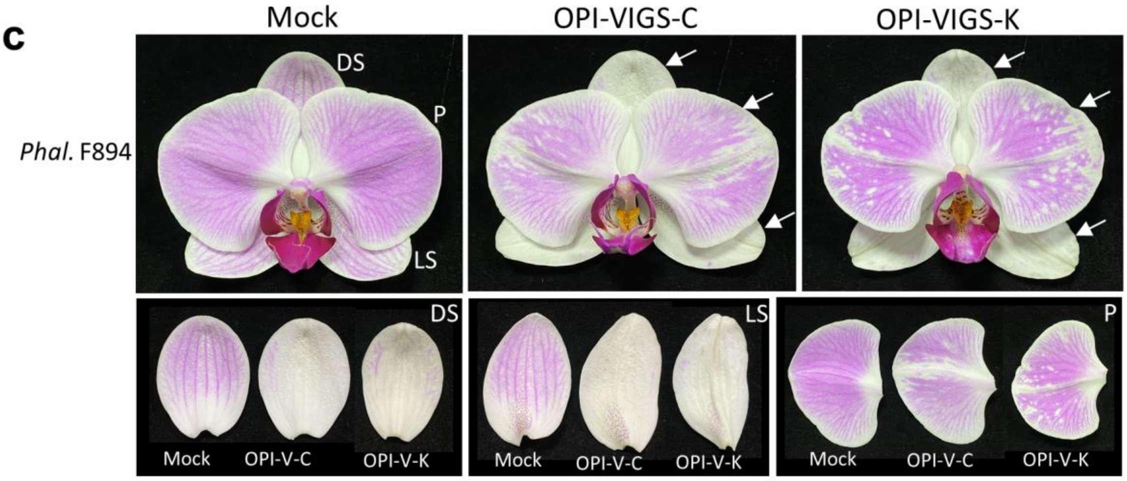

d

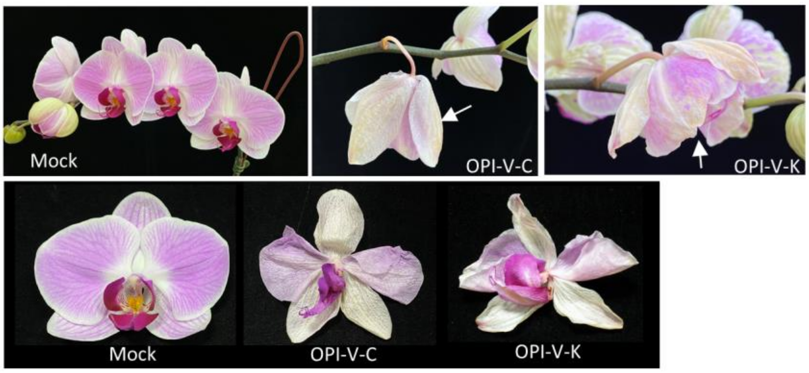

Supplementary Figure 17. The analysis of the two DNA fragments used for the *OPI* VIGS experiment.

a Schematic diagram of the two DNA fragments (one in the K domain, one in the C domain) from *OPI* used for VIGS experiments for *OPI*-VIGS-K and *OPI*-VIGS-C.

b Primers used to amplify two DNA fragments in **(a).**

c Similar reduced pigmentation phenotypes in dorsal sepal (DS), lateral sepal (LS) and petal (P) (arrows) were observed in both *OPI*-VIGS-C and *OPI*-VIGS-K flowers for *Phalaenopsis* F894.

d Similar early senescence phenotypes in sepals/petals (arrows) were observed in both *OPI*-VIGS-C and *OPI*-VIGS-K flowers for *Phalaenopsis* F894.

Supplementary Fig. 18

a

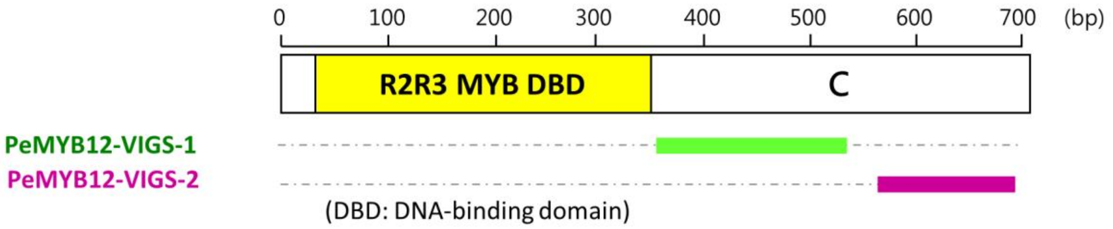

b

Genes	Primer name	Sequence (sequences in bold letters represent recombination sites)
PeMYB12 (C1)	PeMYB12-VIGS-F	5'- **GGGGACAAGTTTGTACAAAAAAGCAGGCTCA**ACCTTAGGTAAAAAGCTTCGCAATCAG -3'
	PeMYB12-VIGS-R	5'- **GGGGACCACTTTGTACAAGAAAGCTGGGTC**GGAGAAGGAACATTTGGAAGGTTTCT -3'
PeMYB12 (C2)	PeMYB12-VIGS-F2	5'- **GGGGACAAGTTTGTACAAAAAAGCAGGCTCA**GCTGTGGCGGTTGAAGCAGCCGCTCAAAA -3'
	PeMYB12-VIGS-R2	5'- **GGGGACCACTTTGTACAAGAAAGCTGGGTC**CTAGATCCAACCGTCAATGTCCATTTCCC -3'

c

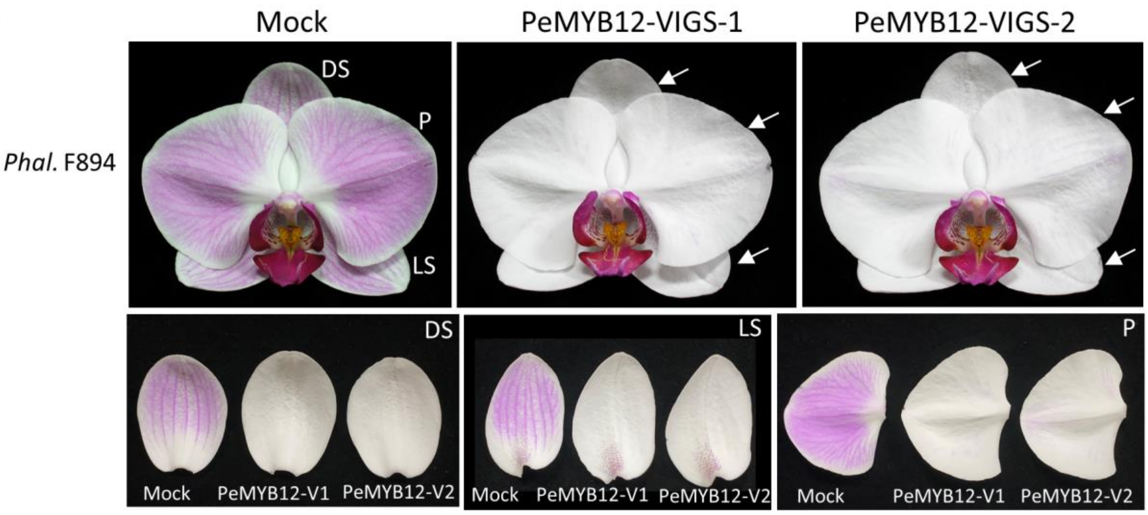

Supplementary Figure 18. The analysis of the two DNA fragments used for the *PeMYB12* VIGS experiment.

a Schematic diagram of the two DNA fragments from *PeMYB12* used for VIGS experiments for *PeMYB12*-VIGS-1 and *PeMYB12*-VIGS-2.

b Primers used to amplify two DNA fragments in **(a).**

c Similar reduced pigmentation phenotypes in dorsal sepal (DS), lateral sepal (LS) and petal (P) (arrows) were observed in both *PeMYB12*-VIGS-1 and *PeMYB12*-VIGS-2 flowers for *Phalaenopsis* F894.

Supplementary Fig. 19

a

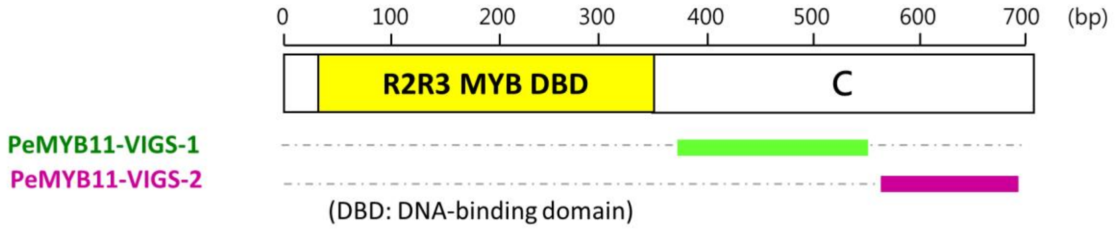

b

Genes	Primer name	Sequence (sequences in bold letters represent recombination sites)
PeMYB11 (C1)	PeMYB11-VIGS-F	5'- **GGGGACAAGTTTGTACAAAAAAGCAGGCTCA**ATCAAGGCATGGAAGCCTAAATCAAAT -3'
	PeMYB11-VIGS-R	5'- **GGGGACCACTTTGTACAAGAAAGCTGGGTC**GCTGGCTAATTTGTTGGGTTTGAAT -3'
PeMYB11 (C2)	PeMYB11-VIGS-F2	5'- **GGGGACAAGTTTGTACAAAAAAGCAGGCTCA**AAGACAAAGTATTAGATTGGGATTCATTT -3'
	PeMYB11-VIGS-R2	5'- **GGGGACCACTTTGTACAAGAAAGCTGGGTC**ATTCCGTCAAAGATTTGCGTCAAATAAGC -3'

c

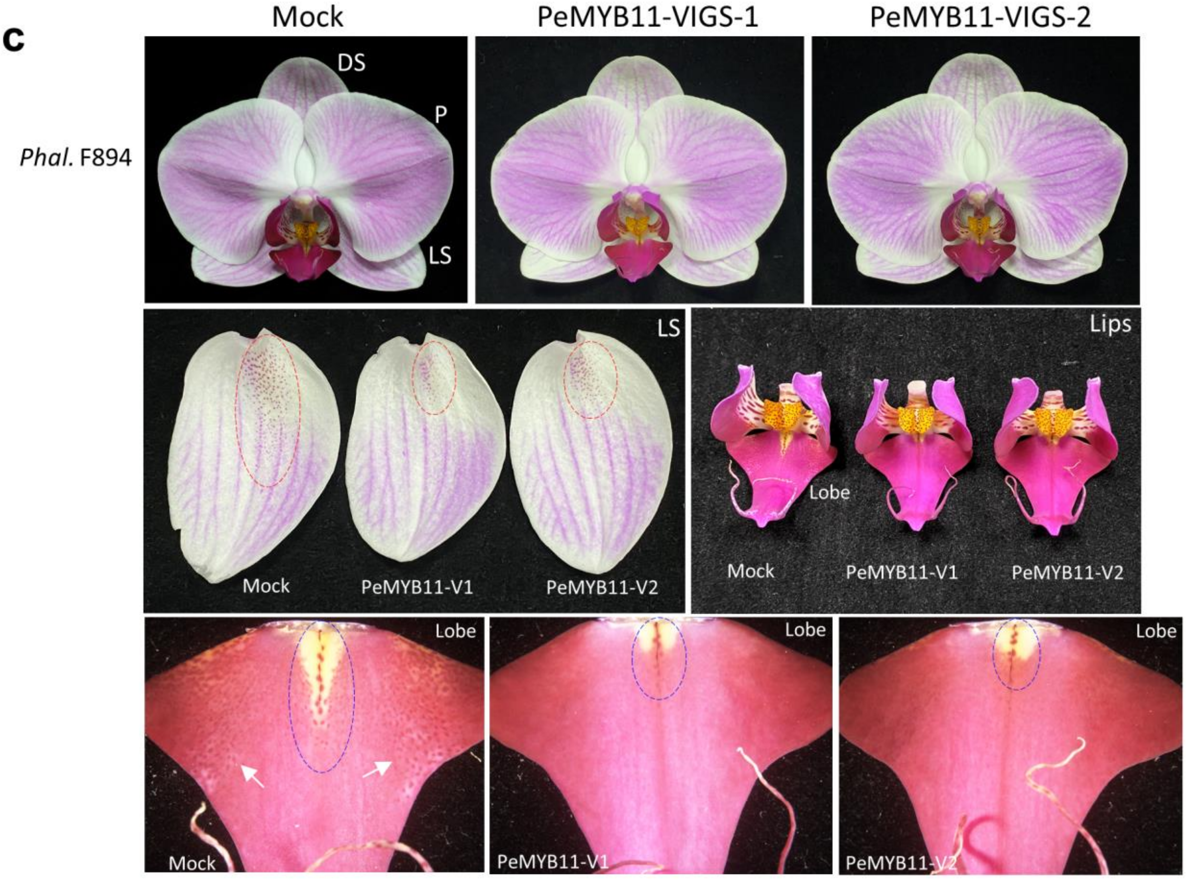

Supplementary Figure 19. The analysis of the two DNA fragments used for the *PeMYB11* VIGS experiment.

a Schematic diagram of the two DNA fragments from *PeMYB11* used for VIGS experiments for *PeMYB11*-VIGS-1 and *PeMYB11*-VIGS-2.

b Primers used to amplify two DNA fragments in **(a).**

c Similar reduced red spot formation phenotypes in lateral sepal (LS) (red cycles) and lobes of lips (arrows and blue cycles) were observed in both *PeMYB11*-VIGS-1 and *PeMYB11*-VIGS-2 flowers for *Phalaenopsis* F894.

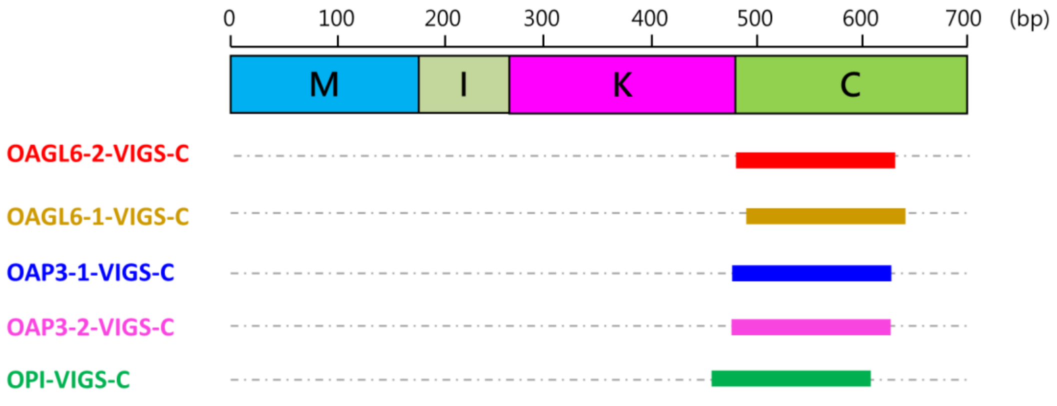

Supplementary Figure 20. Schematic diagram of the DNA fragments used for PeMADS box genes VIGS fragments.

The positions of DNA fragments (in different colors) containing the sequences specific to the C-terminal for *OAGL6-2* (OAGL6-2-VIGS-C), *OAGL6-1* (OAGL6-1-VIGS-C), *OAP3-1* (OAP3-1-VIGS-C), *OAP3-2* (OAP3-2-VIGS-C) and *OPI* (OPI-VIGS-C), which were used for the VIGS experiment, as shown corresponding to their MADS box proteins. M: MADS box domain; I: Intervening domain; K: Keratin-like domain; C: C-terminal domain.

Supplementary Fig. 21

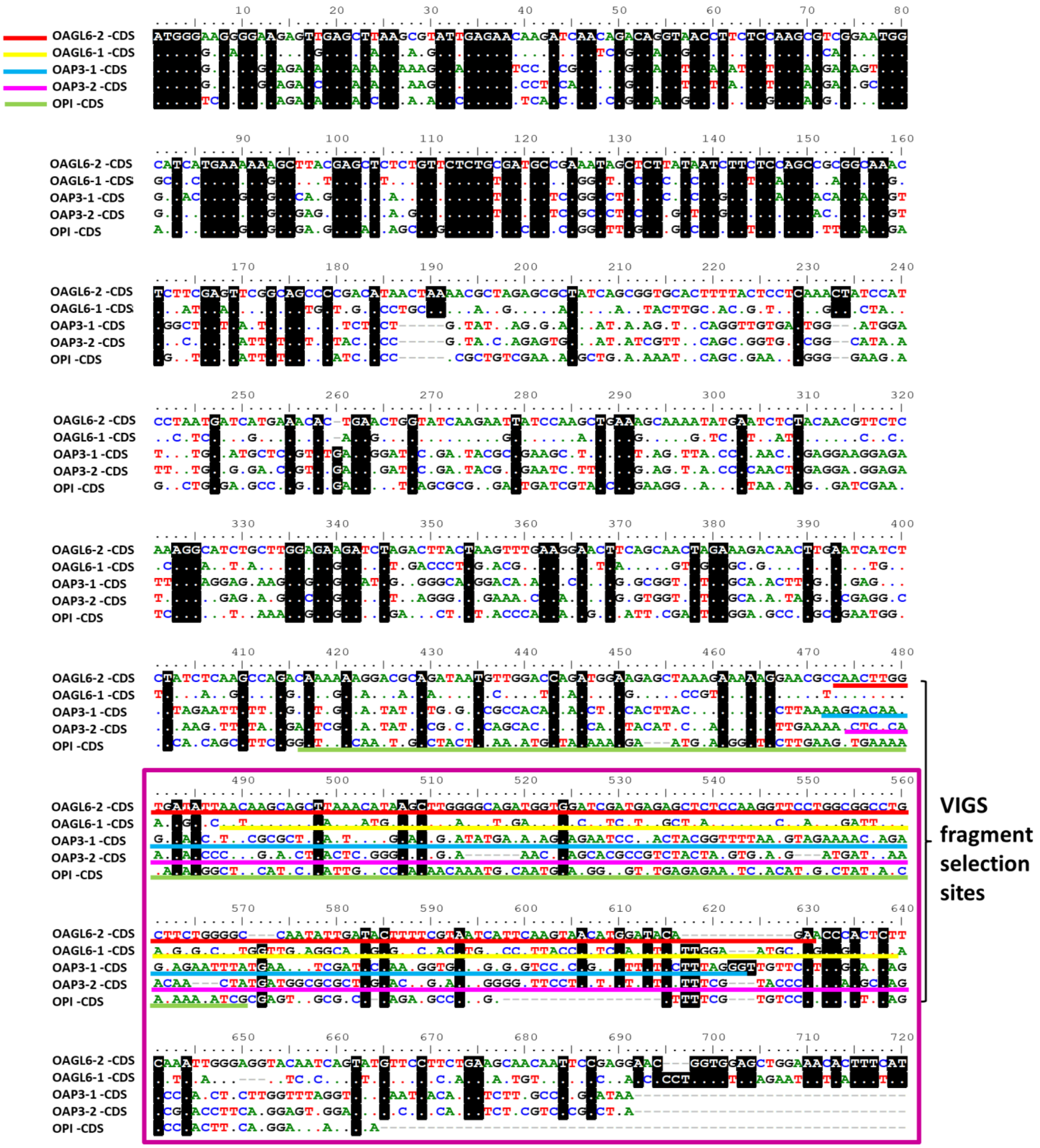

Supplementary Figure 21. DNA sequences used for *PeMADS* box genes VIGS fragments.

The positions of the DNA fragments (underlined in different colors) containing the specific sequences used for VIGS experiments for *OAGL6-2*, *OAGL6-1*, *OAP3-1*, *OAP3-2* and *OPI* in the highly variable region (as VIGS fragment selection sites) in the C-terminal (boxed) regions of their MADS box proteins.

Supplementary Fig. 22

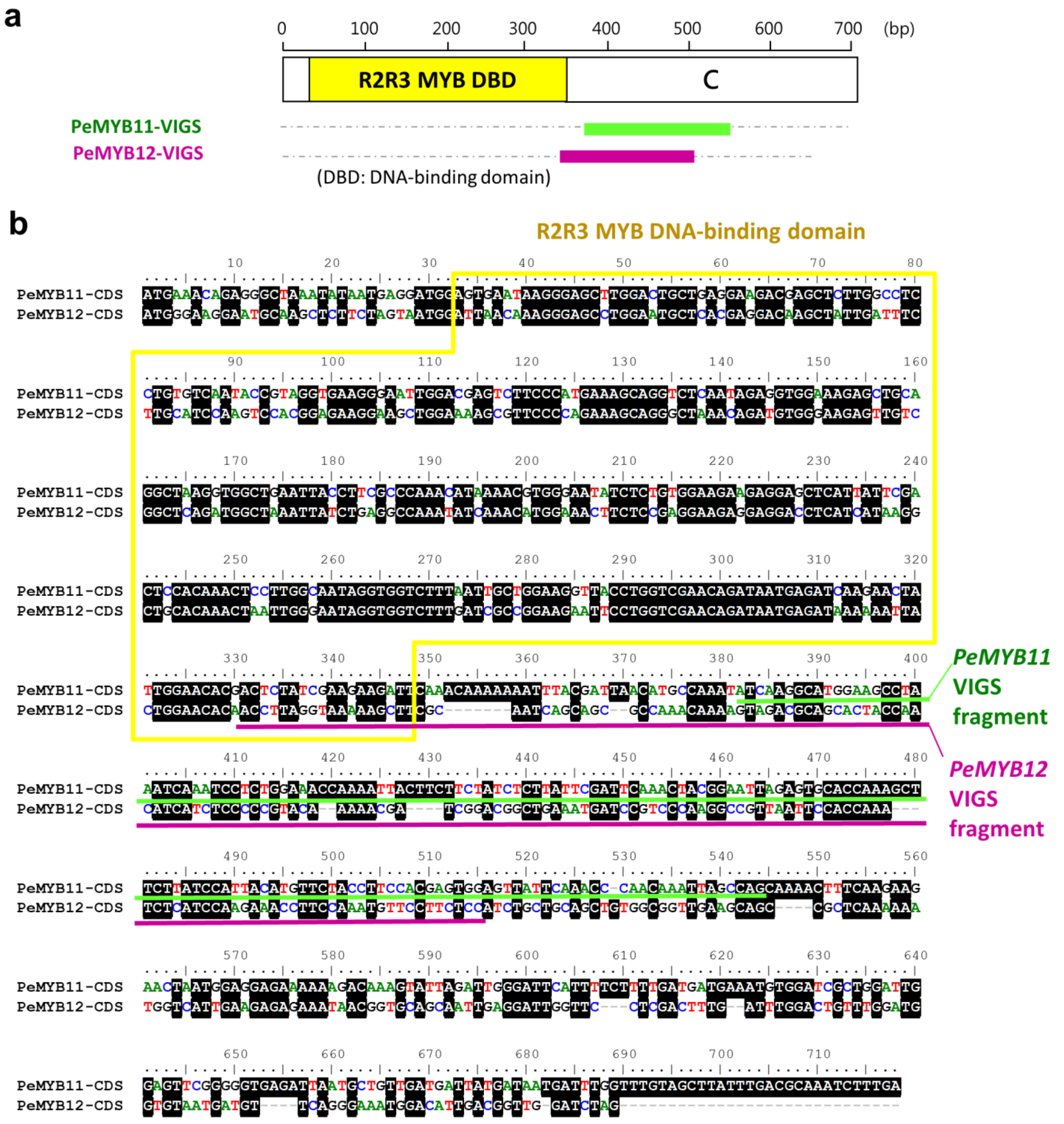

Supplementary Figure 22. The DNA sequences used for *PeMYB11/12* VIGS fragments.

a Schematic diagram for DNA fragments (in different color) used for VIGS experiments for *PeMYB11* and *PeMYB12*.

b Positions of the DNA fragments (underlined in green and purple color) containing the specific sequences used for VIGS experiments for *PeMYB11* and *PeMYB12* were located downstream from the highly conserved R2R3 MYB DNA-binding domain (yellow box).

Supplementary Table 1. Primers used for Q-PCR for MADS box genes of different orchids in this study.

Genes / Species	Primer directions	*OAP3-1*	*OAGL6-1*	*OAP3-2*	*OAGL6-2*	*OPI*
Phalaenopsis spp	forward	PeM2-RT-F	O7-RT-1	PeM3-RT-F	PeM9-RT-F	PeM6-RT-F
	reverse	PeM2-RT-R	PeM10-RT-R	PeM3-RT-R	PeM9-RT-R	PeM6-RT-R
Oncidium spp	forward	O5-RT-1	O7-RT-1	O9-RT-1	O1-RT-1	O8-RT-1
	reverse	O5-RT-2	O7-RT-2	O9-RT-2	O1-RT-2	O8-RT-2
Cattleya spp	forward	PeM2-RT-F	O7-RT-1	PeM3-RT-F	O1-RT-3	O8-RT-1
	reverse	PeM2-RT-R	PeM10-RT-R	PeM3-RT-R	O1-RT-4	O8-RT-3
Paphiopedilum spp	forward	PaphAP33-RT-1	O7-RT-1	PaphAP31-RT-1	O1-RT-3	O8-RT-1
	reverse	PaphAP33-RT-2	PeM10-RT-R	PaphAP31-RT-2	O1-RT-4	O8-RT-3
Dendrobium spp	forward	PeM2-RT-F	O7-RT-1	DenAP32-RT-1	DenAGL6-RT-1	O8-RT-1
	reverse	PeM2-RT-R	O7-RT-2	DenAP32-RT-2	DenAGL6-RT-2	O8-RT-3

Supplementary Table 2. Primer sequences for Q-PCR.

Gene	Primer name	Sequence
OAP3-1	PeM2-RT-F	5'- AGGAAGGAGATTAGGAGGAGGAAG -3'
	PeM2-RT-R	5'- TTCTTGTAAGTGTCAGTTTGTGTGG -3'
	O5-RT-1	5'- ACGAACTGGAGATGAAAGACGAG -3'
	O5-RT-2	5'- GTAGATTGGGTTGAATTGGCTGAG -3'
	PaphAP33-RT-1	5'- GCCACACAAACTGACACTTACAAG -3'
	PaphAP33-RT-2	5'- GCGAATGCTCATCCTTCATCTCC -3'
OAGL6-1	O7-RT-1	5'- CCAGGCAAAGAAAGACACAAATAATG -3'
	O7-RT-2	5'- TGCCATCAACCACAGTATCAGATTCC -3'
	PeM10-RT-R	5'- TGCCTTCAACCACCGCCGC -3'
OAP3-2	PeM3-RT-F	5'- ACTCTCAAGAAACCCACAGGAAC -3'
	PeM3-RT-R	5'- ACGAAATGAATACAAGTAGGAAGCC -3'
	O9-RT-1	5'- TGATGATCCGAACAATTATGATGGTG -3'
	O9-RT-2	5'- TTTGGCTGGCTTGGTTGGG -3'
	PaphAP31-RT-1	5'- CAAGAAACCCACAAGAACCTCATAC -3'
	PaphAP31-RT-2	5'- AGCCATAGCCCATTCCAAGAAAG -3'
	DenAP32-RT-1	5'- ACGGAGCACGCAGTCTATTATG -3'
	DenAP32-RT-2	5'- ACGGAATGAGTACAAGTAGGAAGC -3'
OAGL6-2	O1-RT-1	5'- GAGCACTTCAAGGTTCCAACTG -3'
	O1-RT-2	5'- TTGATCACATTCTCTTAAACAGCCCA -3'
	O1-RT-3	5'- TAAACATAAGCTTGGGGCAGATGGTG -3'
	O1-RT-4	5'- TTTCCAGCTCCACCGTTCCTCG -3'
	PeM9-RT-F	5'- CTTCCTGCAATGTAATTTCAGCTGT -3'
	PeM9-RT-R	5'- AACCAGTCATAGTGTAAAAGTTGAGAA -3'
	DenAGL6-RT-1	5'- GACACAGAACCCACTCTTCATATTG -3'
	DenAGL6-RT-2	5'- ATCAAACTCTTAAACAGCCCATCC -3'
OPI	PeM6-RT-F	5'- ATGGCAATGGAAGGGAGTATGAG -3'
	PeM6-RT-R	5'- TTAAACAACAGGAAAGCAGTAGGC -3'
	O8-RT-1	5'- ATGGAAGGCAGCATGAGAGAAC -3'
	O8-RT-2	5'- AAAGCGTTAGCATTGTTACTTGTTT -3'
	O8-RT-3	5'- TTTCCCTGCAAGTTGGGCTGAAT -3'
PeMYB12	PeMYB12-RT-F	5'- AAATGATCCGTCCCAAGGCCG -3'
	PeMYB12-RT-R2	5'- GCGGCTGCTTCAACCGCCACAGCTGCA -3'
PeMYB11	PeMYB11-RT-F	5'- CAAGAAGAACTAATGGAGGAGAAAAAAGAC -3'
	PeMYB11-RT-R	5'- CAACAGCATTAATCTCACCCCCG -3'
PeSAG39	RT-PeSAG39-F	5'- TGCCGTTTCAGTGTCGGCTT -3'
	RT-PeSAG39-R	5'- GTACACCCTCCCGTGCTTCG -3'

PeEDF1	RT-PeEDF1-F<br>RT-PeEDF1-R	5'- TGCTCCGCAAACACACTTAC -3'<br>5'- CTGCTTCGGAATCACGAGAC -3'
PeEDF2	RT-PeEDF2-F<br>RT-PeEDF2-R	5'- GGCGTCAACTTAGACCAACC -3'<br>5'- GGTGGTACCAAGGCAGAGAT -3'
PeVND1	RT-PeNAC43-F<br>RT-PeNAC43-R	5'- GGGTTCAACGATTGGGCTAC -3'<br>5'- CTGAAAGCCGCCGTTAAGAA -3'
PeMYB85	RT-PeMYB39-F<br>RT-PeMYB39-R	5'- GCATCAGTTGAGCAGAAGCA -3'<br>5'- CCATCCCACGCATCAAATGT -3'
PeMYB46	RT-PeMYB46-F<br>RT-PeMYB46-R	5'- AGCTTGCTGCACGATAATGG -3'<br>5'- TGGCACCATTCTCTTCTTGAC -3'
PeMYB63	RT-PeZM1-F<br>RT-PeZM1-R	5'- CCACAACTTGCTCGGAAACA -3'<br>5'- TTGATCTCGTTGTCGGTTCT -3'
PeBOP1	RT-PeBOP1-F<br>RT-PeBOP1-R	5'- CGGCTAGGCTTGATTGTGAT -3'<br>5'- AAGAAGAAGGCCCGATACCT -3'
PeBOP2	RT-PeBOP2-F<br>RT-PeBOP2-R	5'- GTGGGAAGGATGGACTGTGAC -3'<br>5'- AAGACAAGCCTATCCCACCA -3'
LMYB12	MYB12-RT-F2<br>MYB12-RT-R2	5'- GAAGAAACAGGGTGAAGCTG -3'<br>5'- CAACTTCGGAATCACTCCAAAG -3'
LAGL6	LAGL6-RT-F2<br>LAGL6-RT-R2	5'- GATGGACCAGATGGAAGAA -3'<br>5'- AAGGACAGCATTGGAGTT -3'
LMADS1/LAP3	L1-RT-F3<br>L1-RT-R3	5'- GGATTGGACATCAAGGAC -3'<br>5'- TTCTTGTAAGTCTCTGTCTG -3'
LMADS8/LPI	L8-RT-F2<br>L8-RT-R2	5'- AAGGATACTGGAAGAGGATAA -3'<br>5'- TGTAAATTAGGCTGGATTGG -3'
LMADS9/LPI	L9-RT-F3<br>L9-RT-R3	5'- ATCTGAACTCACTTCAGCCT -3'<br>5'- TGTCCCTCATATTCTCCCC -3'
AtAP3	RT-AtAP3-F<br>RT-AtAP3-R	5'- ATCGAAGGGTCACGTGCTTA -3'<br>5'- CATGAAGGCCATGGTTGGG -3'
AtPI	RT-AtPI-F<br>RT-AtPI-R	5'- GAGAAGATGATGGCGGAGGA -3'<br>5'- CCTCTTGCGTTGCTTGCTAT -3'
OnAT	OnAT-RT-3<br>OnAT-RT-4	5'- GGATTAGGCTCTCTGCTGTTGG -3'<br>5'- GTGTGGATAAGACGCTGTTGTATG -3'
PACT4	PACT4-RT-F<br>PACT4-RT-R	5'- CTTACTGAGGCACCGCTGAAC -3'<br>5'- CCATCACCAGAATCAAGAACAATACC -3'
LilyACTIN	Liact-F<br>Liact-R	5'- ATCCCAGCAGCGTCGCACATCC -3'<br>5'- GCCAGATCTTCTCCATGTCATCC -3'
AtUBQ10	UBQ10-F<br>UBQ10-R	5'- CTCAGGCTCCGTGGTGGTATG -3'<br>5'- GTGATAGTTTTCCCAGTCAACGTC -3'

Supplementary Table 3. Primer sequences for construction of FRET constructs.

Genes	Primer name	Sequence
OAGL6-1	PeM10-FRET-F-XbaI	5'- TCTAGAATGGGGAGAGGAAGAGTGGAGC -3'
(PeMADS10)	PeM10-FRET-R-ns-kpnI	5'- GGTACCGAGCATCCATCCAAGCATAAAATTATTATTC -3'
OAGL6-2	PeM9-FRET-F-pstI	5'- CTGCAGATGGGAAGGGGAAGAGTTGA -3'
(PeMADS9)	PeM9-FRET-R-ns-BamHI	5'- GGATCCAATGCGCCCCAGCCAGGC -3'
OAP3-1	PeM2-FRET-F-XbaI	5'- TCTAGAATGGGGAGGGGGAAGATA -3'
(PeMADS2)	PeM2-FRET-R-ns-BamHI	5'- GGATCCTGCAAGGCTAAGATCATGTGATTC -3'
OAP3-2	PeM3-new-FRET-F-XbaI	5'- TCTAGAATGGGGAGGGGGAAGATCGAG -3'
(PeMADS3)	PeM3-FRET-R-ns-kpnI	5'- GGTACCGGCGAGACGTAGATCATGAG -3'
OPI	PeM6-FRET-F-XbaI	5'- TCTAGAATGGGTCGGGGAAAGATAGA -3'
(PeMADS6)	PeM6-FRET-R-ns-kpnI	5'- GGTACCCTTATTTCCCTGCAAGTTGGGCT -3'

Supplementary Table 4. Primer sequences for VIGS target DNA insertions in pCymMV-Gateway .

Genes	Primer name	Sequence (sequences in bold letters represent recombination sites)
OAGL6-1	PeM10-VIGS-F	5'- **GGGGACAAGTTTGTACAAAAAAGCAGGCTCA**ATAAGCAGCTAAAAATGAAGCTTGAG -3'
(PeMADS10)	PeM10-VIGS-R	5'- **GGGGACCACTTTGTACAAGAAAGCTGGGTC**GTAGAGTCGGCTCGCATTCCAATGAA -3'
OAGL6-2	PeM9-VIGS-F	5'- **GGGGACAAGTTTGTACAAAAAAGCAGGCTCA**GGTGATATTAACAAGCAGCTTAAACA -3'
(PeMADS9)	PeM9-VIGS-R	5'- **GGGGACCACTTTGTACAAGAAAGCTGGGTC**TTTGAAGAGTGGGTTCTGTATCCATG -3'
OAP3-1	PeM2-VIGS-F	5'- **GGGGACAAGTTTGTACAAAAAAGCAGGCTCA**AGGGAAACTTACCGCGCTCTAATAC -3'
(PeMADS2)	PeM2-VIGS-R	5'- **GGGGACCACTTTGTACAAGAAAGCTGGGTC**TGAACAACCCTAAAGGAAAACATCT -3'
OAP3-2	PeM3-VIGS-F	5'- **GGGGACAAGTTTGTACAAAAAAGCAGGCTCA**AGAAACCCACAGGAACTTACTC -3'
(PeMADS3)	PeM3-VIGS-R	5'- **GGGGACCACTTTGTACAAGAAAGCTGGGTC**GTTCGGCTGGCTTGGTTGGGTACGA -3'
OPI	PeM63-VIGS-F	5'- **GGGGACAAGTTTGTACAAAAAAGCAGGCT**AATGGACTACTTGAAGATGCTAAAAAAGA -3'
(PeMADS6)	PeM63-VIGS-R	5'- **GGGGACCACTTTGTACAAGAAAGCTGGGT**CGCATACTCGCGATCTTTATGATGATAGCC -3'
PeMYB12	PeMYB12-VIGS-F	5'- **GGGGACAAGTTTGTACAAAAAAGCAGGCTCA**ACCTTAGGTAAAAAGCTTCGCAATCAG -3'
	PeMYB12-VIGS-R	5'- **GGGGACCACTTTGTACAAGAAAGCTGGGTC**GGAGAAGGAACATTTGGAAGGTTTCT -3'
PeMYB11	PeMYB11-VIGS-F	5'- **GGGGACAAGTTTGTACAAAAAAGCAGGCTCA**ATCAAGGCATGGAAGCCTAAATCAAAT -3'
	PeMYB11-VIGS-R	5'- **GGGGACCACTTTGTACAAGAAAGCTGGGTC**GCTGGCTAATTTGTTGGGTTTGAAT -3'

Supplementary Table 5. VIGS experiments *on Phalaenopsis* V3.

VIGS constructs	Experiments	Total inoculated plants	The number of affected plants	Observed phenotypes	Inoculation date
pCymMV	Exp. 1	3	0	Wild type-like	2017-05-16
	Exp. 2	3	0		2017-07-07
	Exp. 3	3	0		2017-09-01
	Exp. 4	3	0		2017-11-07
	Exp. 5	3	0		2017-11-28
	Exp. 6	3	0		2018-04-17
	Exp. 7	5	0		2018-08-07
	Exp. 8	4	0		2018-10-05
	Exp. 9	3	0		2018-12-07
	Exp. 10	3	0		2019-01-18
	Exp. 11	2	0		2019-06-11
	Exp. 12	3	0		2019-08-20
	Exp. 13	3	0		2019-11-15
	Exp. 14	3	0		2020-01-04
	Exp. 15	3	0		2020-03-31
Sum		47	0		
OAGL6-2 (PeMADS9)	Exp. 1	3	3	(1) Lips converted into enlarged petal/sepal-like structures; (2) Spots on lateral sepals disappeared	2017-05-16
	Exp. 2	3	3		2017-07-07
	Exp. 3	3	3		2017-09-01
	Exp. 4	5	5		2017-11-07
	Exp. 5	3	3		2017-11-28
	Exp. 6	3	3		2018-04-17
	Exp. 7	13	13		2018-08-07
	Exp. 8	12	12		2018-10-05
	Exp. 9	3	3		2018-12-07
	Exp. 10	3	3		2019-01-18
	Exp. 11	3	3		2019-06-11
	Exp. 12	3	3		2019-08-20
	Exp. 13	3	3		2019-11-15
	Exp. 14	3	3		2020-01-04
	Exp. 15	3	3		2020-03-31
Sum		66	66		
OAGL6-1 (PeMADS10)	Exp. 1	3	3	(1) Senescent flowers not abscised; (2) Lateral sepals with an enhanced curvature of the edge	2017-05-16
	Exp. 2	3	3		2017-07-07
	Exp. 3	3	3		2017-09-01
	Exp. 4	5	5		2017-11-07
	Exp. 5	3	3		2017-11-28
	Exp. 6	3	3		2018-04-17
	Exp. 7	3	3		2018-08-07
	Exp. 8	4	4		2018-10-05
	Exp. 9	3	3		2018-12-07
	Exp. 10	3	3		2019-01-18
	Exp. 11	3	3		2019-06-11
	Exp. 12	3	3		2019-08-20
	Exp. 13	3	3		2019-11-15
	Exp. 14	3	3		2020-01-04
	Exp. 15	3	3		2020-03-31
Sum		48	48		
OAP3-1 (PeMADS2)	Exp. 1	3	3	Early senescence of sepals	2018-12-07
	Exp. 2	3	3		2019-06-11
	Exp. 3	3	3		2019-08-20
	Exp. 4	3	3		2019-11-15
	Exp. 5	3	3		2020-01-04
	Exp. 6	3	3		2020-03-31
Sum		18	18		
OAP3-2 (PeMADS3)	Exp. 1	3	0	Wild type-like	2018-12-07
	Exp. 2	3	0		2019-06-11
	Exp. 3	3	0		2019-08-20
	Exp. 4	3	0		2019-11-15
	Exp. 5	3	0		2020-01-04
	Exp. 6	3	0		2020-03-31
Sum		18	0		
OPI (PeMADS6)	Exp. 1	5	5	Early senescence of sepals	2019-09-03
	Exp. 2	3	3		2019-11-15
	Exp. 3	3	3		2020-01-04
	Exp. 4	3	3		2020-03-31
Sum		14	14		

Supplementary Table 6. VIGS experiments *on Phalaenopsis* F894.

VIGS constructs	Experiments	Total inoculated plants	The number of affected plants	Observed phenotypes	Inoculation date
pCymMV	Exp. 1	3	0	Wild type-like	2017-09-01
	Exp. 2	5	0		2017-11-16
	Exp. 3	3	0		2018-01-19
	Exp. 4	3	0		2019-03-19
	Exp. 5	2	0		2019-06-28
	Exp. 6	3	0		2019-08-20
	Exp. 7	2	0		2019-11-15
	Exp. 8	3	0		2020-01-04
	Exp. 9	3	0		2020-03-31
Sum		27	0		
OAGL6-2 (PeMADS9)	Exp. 1	3	3	(1) Lips converted into enlarged petal/sepal-like structures; (2) Spots on lateral sepals disappeared	2017-09-01
	Exp. 2	5	5		2017-11-16
	Exp. 3	3	3		2018-01-19
	Exp. 4	3	3		2019-03-19
	Exp. 5	2	2		2019-06-28
	Exp. 6	3	3		2019-08-20
	Exp. 7	2	2		2019-11-15
	Exp. 8	3	3		2020-01-04
	Exp. 9	3	3		2020-03-31
Sum		27	27		
OAGL6-1 (PeMADS10)	Exp. 1	3	3	(1) Decreased pigmentations in petals/sepals; (2) Senescent flowers not abscised; (3) Lateral sepals with an enhanced curvature of the edge	2017-09-01
	Exp. 2	5	5		2017-11-16
	Exp. 3	3	3		2018-01-19
	Exp. 4	3	3		2019-03-19
	Exp. 5	3	3		2019-06-28
	Exp. 6	3	3		2019-08-20
	Exp. 7	3	3		2019-11-15
	Exp. 8	3	3		2020-01-04
	Exp. 9	3	3		2020-03-31
Sum		29	29		
OAP3-1 (PeMADS2)	Exp. 1	3	3	(1) Decreased pigmentations in sepals; (2) Early senescence of sepals	2019-03-19
	Exp. 2	3	3		2019-06-28
	Exp. 3	3	3		2019-08-20
	Exp. 4	3	3		2019-11-15
	Exp. 5	3	3		2020-01-04
	Exp. 6	3	3		2020-03-31
Sum		18	18		
OAP3-2 (PeMADS3)	Exp. 1	3	3	Decreased pigmentations in petals	2019-03-19
	Exp. 2	2	2		2019-06-28
	Exp. 3	3	3		2019-08-20
	Exp. 4	3	3		2020-01-04
	Exp. 5	3	3		2020-03-31
Sum		14	14		
OPI (PeMADS6)	Exp. 1	2	2	(1) Decreased pigmentations in petals/sepals; (2) Early senescence of sepals	2019-11-15
	Exp. 2	3	3		2020-01-04
	Exp. 3	3	3		2020-03-31
Sum		8	8		
PeMYB11	Exp. 1	3	3	Red spot formation in lateral sepals reduced	2019-04-23
	Exp. 2	3	3		2019-06-28
	Exp. 3	3	3		2019-08-20
	Exp. 4	2	2		2019-11-15
	Exp. 5	3	3		2020-01-07
Sum		14	14		
PeMYB12	Exp. 1	3	3	Decreased pigmentations in petals/sepals	2019-04-23
	Exp. 2	3	3		2019-06-28
	Exp. 3	3	3		2019-08-20
	Exp. 4	3	3		2019-11-15
	Exp. 5	3	3		2020-01-07
	Exp. 6	3	3		2020-03-31
Sum		18	18		

Supplementary Table 7. VIGS experiments *on Phalaenopsis* KA38501.

VIGS constructs	Experiments	Total inoculated plants	The number of affected plants	Observed phenotypes	Inoculation date
pCymMV	Exp. 1	3	0	Wild type-like	2019-09-03
	Exp. 2	2	0		2019-11-15
	Exp. 3	5	0		2020-01-21
Sum		10	0		
OAGL6-2 (PeMADS9)	Exp. 1	3	3	(1) Lips converted into enlarged petal/sepal-like structures; (2) Spots on lateral sepals disappeared	2019-09-03
	Exp. 2	2	2		2019-11-15
	Exp. 3	3	3		2020-01-21
Sum		8	8		
OAGL6-1 (PeMADS10)	Exp. 1	4	4	(1) Decreased pigmentations in petals/sepals; (2) Senescent flowers not abscised; (3) Lateral sepals with an enhanced curvature of the edge	2019-09-03
	Exp. 2	3	3		2019-11-15
	Exp. 3	5	5		2020-01-21
Sum		12	12		
OAP3-1 (PeMADS2)	Exp. 1	3	3	(1) Decreased pigmentations in sepals; (2) Early senescence of sepals	2019-11-15
	Exp. 2	5	5		2020-01-21
Sum		8	8		
OAP3-2 (PeMADS3)	Exp. 1	2	2	Decreased pigmentations in petals	2019-11-15
	Exp. 2	3	2		2020-01-21
Sum		5	4		
OPI (PeMADS6)	Exp. 1	2	2	(1) Decreased pigmentations in petals/sepals; (2) Early senescence of sepals	2019-11-15
	Exp. 2	5	5		2020-01-21
Sum		7	7		
PeMYB11	Exp. 1	3	2	Spot formation in lateral sepals reduced	2019-09-03
	Exp. 2	3	2		2019-11-15
	Exp. 3	3	2		2020-01-21
Sum		9	6		
PeMYB12	Exp. 1	3	3	Decreased pigmentations in petals/sepals	2019-09-03
	Exp. 2	3	3		2019-11-15
	Exp. 3	5	5		2020-01-21
Sum		11	11		

Supplementary Reference

1. Suzuki, K. et al. RNA-seq-based evaluation of bicolor tepal pigmentation in Asiatic hybrid lilies (*Lilium* spp.). BMC Genomics **17**, 611 (2016).
