# Supplementary material for: Multifunctional evolution of B and AGL6 MADS box genes in orchids
Source: Nat Commun. 2021 Feb 10;12:902. doi: 10.1038/s41467-021-21229-w (PMC7876132; doi:10.1038/s41467-021-21229-w)
Supplement: Supplementary file 2 — Peer Review File [file 41467_2021_21229_MOESM2_ESM.pdf]

Reviewer #1 (Remarks to the Author):

The manuscript by Hsu et al. aims to further elucidate the functions of B-class and AGL6 MADS-box proteins in orchids using VIGS, and proposes new roles for these genes in petal senescence, petal pigmentation and pedicel abscission. The manuscript is illustrated with beautiful figures and these results are potentially of broad interest for plant biology , but much to my regret, all of the conclusions and new findings are solely based on crude VIGS experiments, with the latter having a notorious reputation for causing off-target effects (note that a stretch of 21 nucleotides is sufficient to silence unrelated genes, and thus absolutely requiring independent proof).

Since at least more than a decade, it has become completely unacceptable in plant science to present gene functional studies for publishing, solely based on one mutant allele (being either T-DNA, natural mutation, transposon, EMS, radiation etcetera), even in most low-impact plant specific journals. Always additional proof is requested under the form of complementation assays or the generation of independent mutant alleles.

Despite that VIGS is a very quick and easy method and requiring very few work compared to the generation of stable transgenics/mutant alleles, in this manuscript, the authors have made one VIGS construct per gene only (which is even worse than having only one 'mutant allele' in terms of reliability), and have used only three plants for each VIGS experiment, which is very low compared to the other labs that work with VIGS (>20-100 plants/experiment). Moreover, the authors do not at all seem to question possible problems with VIGS, and are not doing any effort to convince the reader that the VIGS silencing effects might be specific for the targeted genes.

Given its notorious reputation, in my opinion using VIGS as the only tool for gene functional analysis is only acceptable in 1) plant species that cannot be transformed (thus excluding the generation of stable transgenics), AND 2) if the experiments have been properly conducted: that is, targeting at least 2 independent non-conserved regions (e.g. 5'UTR and 3'UTR) in separate experiments and having the same outcome 3) based on sufficient numbers of plants resulting in statistically sound results. 4)If possible, backed up with functional assays in transformable species.

Unfortunately, none of the above criteria have been fulfilled in this manuscript.

Note that in the last couple of years several protocols have been published for stable Agrobacterium based transformation of orchids (including Phalaenopsis orchids used in this study), therefore the authors might consider generating stable CRISPR-Cas9 alleles instead of relying on VIGS for functional analysis. This would strongly enhance the scientific impact of their future studies in orchids.
For the current study, the authors might find it too time-consuming to generate stable alleles, but should at least redo the VIGS experiments using separately 5' and 3' UTR regions of the targeted genes, and on a higher number of plants.

For the case of petal senescence, the authors have sought to generate additional proof by an expression analysis of B-class genes in Arabidopsis WT and the etr1 mutant, but this only a further correlation. I do not understand why the authors have not analyzed petal senescence in 35S:AP3 or 35S:PI lines, which should have delayed petal senescence if the claim of the authors would be correct, and would have been a more convincing experiment than the one presented in the manuscript.

Similarly, for identification of protein complexes , usually independent techniques are required as additional proof. In this study, everything is based on one technique (FRET).

Reviewer #2 (Remarks to the Author):

In this paper, Hsu et al investigate the function of MADS-box genes in orchid flower development. They use VIGS to show that different MADS-box paralogs affect flower development in subtly different ways. In addition, they show that B-class genes have a role in regulating flower senescence, and AGL6 genes have roles in floral abscission. There is an impressive amount of data in this paper, which I enjoyed reading. My suggestions for improvement are below, listed in order of importance:

1. The authors show that these MADS-box complexes may form quartets using FRET analysis in Nicotiana benthamiana leaves (Extended Data Fig. 3), they also show through VIGS that individual genes have distinct functions in flower development. From this they infer that (a) the same quartets that they detected in Nicotiana form in orchids, and (b) that it is differentiation between these quartets that is critical in the variation in gene function they observe in their VIGS analysis. These inferences are never made clear. Instead, the authors say things like, 'Here we show the existence of functional L' and SP' complexes in orchid and to find that AP3/PI/AGL6 genes have evolved to acquire novel functions during evolution' (sentence from the abstract). They do not show the existence of any MADS-quartets in orchids. The language around this point should be softened, and the inferences the authors are making should be made clear throughout the manuscript. The whole abstract needs to be re-written to more accurately reflect the authors' findings.
2. Similarly, the authors cannot say much about evolution. There is no outgroup in their analyses or discussion. They are assuming that because what they have observed in their VIGS experiments has not been reported for eudicots (but see below), that the orchid gene function must be novel. Without outgroups, the authors have no way of knowing whether the 'novel' functions they have observed are derived or ancestral. Statements about evolution like the one highlighted above, and in the discussion should be clarified or removed.
3. A function for an E-class gene in abscission has been described – the j2 mutant of tomato (Soyk et al, 2017, Cell 169:1142–1155). This paper has not been cited, and should definitely be discussed in this manuscript.
4. One aspect of this paper that I particularly liked was the functions for floral MADS-box genes outside of floral organ identity specification. Apart from the abscission mentioned above, there is a role for PI-like genes in suppressing fruit development in apple (Yao et al, 2001, PNAS 98: 1306–1311). There may be other examples, at least this Yao et al paper should be included/discussed in this manuscript.
5. In the figures, the authors label AGL6 'OL6' or 'L6', but call the genes in the flower images. The figures would be easier to interpret if the labeling was kept standard – and all were labeled as 'AGL6'in the figures, text, and figure legends. This is particularly confusing because the authors refer to L and L' quartets, distinct to the L6 genes.

Reviewer #3 (Remarks to the Author):

The manuscript entitled "Multifunctional evolution of B and AGL6 MADS box genes in orchids" by Hsu al. was reviewed for publication in Nature Communications manuscript (NCOMMS-20-26415-T). This article is a tour de force of molecular genetics of orchid flower development, exploring the molecular components of petal, sepal, lip identities, in addition to and flower pigmentation, senescence and pedicel abscission. This is an extremely data rich manuscript that takes a lot of time to go through and follow, but the authors have done an outstanding job in presenting this deep and wide-ranging data set on orchid flower development.

Comments and Corrections

Figure 1q, Ds and Ls are not capitalized like in the text and legend, change to DS and LS, or does the lower case have meaning?

Figure 2, while in Figure 1, the VIGS experiments are indicated by "VIGS" at the end of the name (e.g. Figure 1l, "F894-OL6-2-VIGS"), which helps the reader evaluate the data, in Figure 2, the headings do not include "-VIGS", which is confusing at first after Figure 1. These figures are data rich, to say the least, small improvements to help the reader evaluate the data is helpful. In addition, the "V" label was then again used in Figure 3, which helps. My suggestion is to use the same symbol, the simplest, "V", to indicate VIGS plants in all Figures. To give an example how this can be confusing, for Figure 2k, at first glance, it is not clear these are VIGS plants, but indeed they are. In addition, the "M" in Figure k-1 is not defined in the legend, I suppose "M" stands for mock?

Figure 4, while the abscission zone (AZ) cells clearly look different between the control and the L6-1-V (Figure 4j and k), typically the AZ is observed best by longitudinal sections. In addition, it is not clear at what position (in expanded data file, it is stated "slices were cut from stalk base) in the pedicel the samples were taken for the micrographs and histology shown in Figure 4 j-k, g and h. The claim that the AZ is not developed in the L6-1-V may be true, but the anatomical data shown in the manuscript do not clearly show this. To be sure, longitudinal sections that show clearly the AZ in wildtype should be compared with those of the L6-1-V plants. The rest of the data is very convincing, i.e. lower BOP1/2 expression and increase in lignification, but it would be better to show clearly the lack of AZ in these VIGS plants by longitudinal sections. An increase in lignin as shown in Figure 4g and h could take place in the AZ without the abolishment of the AZ entirely. Without this extra data, I do not agree that it can be concluded that the AZ has not developed, or has been abolished as concluded.

How do the authors conclude that there is an unknown "X" protein for senescence and unknown "Y" protein for pedicel abscission? I am not clear on how that can be concluded. I am also unclear how it can be concluded that there are complexes involved in both these processes? Couldn't this data be explained without the formation of a complex?

Answer to Reviewer #1:

1. Since at least more than a decade, it has become completely unacceptable in plant science to present gene functional studies for publishing, solely based on one mutant allele (being either T-DNA, natural mutation, transposon, EMS, radiation etcetera), even in most low-impact plant specific journals. Always additional proof is requested under the form of complementation assays or the generation of independent mutant alleles.

Ans: (1) Since the VIGS technique is based on the knockdown of a target gene expression and sequentially created a knockdown (not knockout) mutant for the gene, we think each independent VIGS experiment for a target gene could be thought to represent an independent event for generating a similar silencing mutant phenotype with a different degree of silencing for a gene.

(2) In orchid, many reports for functional analysis of various genes using the VIGS technique have been published during the past years, including the one we published in Nature Plants (Hsu et al., Nature Plants 1, 15046, 2015) regarding the finding of a conserved principle (Perianth code) for orchid perianth formation. Thus, this technique has become very useful and has been considered and accepted as a method to study orchid gene function.

2. Despite that VIGS is a very quick and easy method and requiring very few work compared to the generation of stable transgenics/mutant alleles, in this manuscript, the authors have made one VIGS construct per gene only (which is even worse than having only one 'mutant allele' in terms of reliability), and have used only three plants for each VIGS experiment, which is very low compared to the other labs that work with VIGS (>20-100 plants/experiment). Moreover, the authors do not at all seem to question possible problems with VIGS, and are not doing any effort to convince the reader that the VIGS silencing effects might be specific for the targeted genes.

Ans: (1) As with the answer for question #1, many reports of the functional analysis of various genes in orchid using the VIGS technique have been published during the past years, including the one we published in Nature Plants (Hsu et al., Nature Plants 1, 15046, 2015) regarding the finding of a conserved principle (Perianth code) for orchid perianth formation. Thus, this technique has become very useful and has been considered and accepted as a method to study orchid gene function.

(2) Regarding the question about one VIGS construct per gene only, before the VIGS experiments performed in this study, we did test the effects caused by using different independent nonconserved regions for a target gene. For example, two different

regions (one in K/C domain, one in C domain) for *OAGL6-2* have been used to construct two VIGS constructs (*OAGL6-2*-VIGS-K/C and *OAGL6-2*-VIGS-C) for *OAGL6-2* and the phenotypes of the VIGS-plants analyzed. The result showed that a similar big lips phenotype (lips converted into sepal/petal) was observed in both kinds of *OAGL6-2*-VIGS-plants. One new Extended Data Figure 13 describing these results was provided in the revised manuscript. Thus, we thought that using one *OAGL6-2*-VIGS construct (with the sequences in the highly variable C-terminal region) should be sufficient to obtain the silencing phenotype for the *OAGL6-2* gene. This assumption should also be true for other target genes as long as the DNA fragments chosen contained highly gene-specific sequences for the targeted genes to ensure that the VIGS silencing effects were specific for the targeted genes. Therefore, in subsequent VIGS experiments in this study, just one DNA fragment specific for a target gene was chosen for a *Phalaenopsis* genes VIGS experiment.

(3) Since our target MADS box genes for the VIGS experiment are *AGL6*- and B-function MADS box genes, the sequences selected and used for each gene must be highly gene-specific, as explained above, to avoid any off-target effects. Thus, in this study, the sequences were checked very carefully, and the fragment (approximately 150 bp) containing the specific sequences in the highly variable C-terminal region for each target MADS box gene was designed, amplified and inserted into the VIGS vector pCymMV-Gateway for the VIGS experiment. Two new Extended Data Figures 14 and 15 describing the detailed positions and corresponding sequences in the gene used in the VIGS experiment for each *Phalaenopsis* MADS box gene are provided in the revised manuscript. For *PeMYB11* and *PeMYB12* genes, specific sequences for each gene in the variable region located downstream from the highly conserved R2R3 MYB DNA-binding domain were chosen for the VIGS experiment. A new Extended Data Figure 16 describing the detailed position and corresponding sequences in the gene used in the VIGS experiment for Phalaenopsis *PeMYB11* and *PeMYB12* genes was provided in the revised manuscript.

(4) Regarding the question about only three plants for each VIGS experiment, we apologize for not clarifying how many plants we eventually used for each gene in the VIGS experiment. We stated that only three plants were used for each VIGS experiment in the Methods section. However, it is only the description of the procedure that we used in each VIGS experiment. The real fact is that we performed many VIGS experiments for each gene tested in different varieties of *Phalaenopsis* orchids. For example, for *Phalaenopsis* V3, within a three-year period (from 2017-05-16 to 2020-03-31), 15 independent VIGS experiments were performed for *OAGL6-2*, and a total 66 plants with the same mutant phenotype were obtained and analyzed. For *OAGL6-1*, a total 15 independent VIGS experiments were performed,

and a total of 48 plants with the same mutant phenotype were obtained and analyzed. For *OAP3-1*, a total 6 independent VIGS experiments were performed, and a total of 18 plants with the same mutant phenotype were obtained and analyzed. For *OAP3-2*, a total 6 independent VIGS experiments were performed, and a total of 18 plants were obtained and analyzed (since V3 flowers are white, no pigmentation change was observed). For *OPI*, a total 4 independent VIGS experiments were performed, and a total of 14 plants with the same mutant phenotype were obtained and analyzed. VIGS was not performed for *PeMYB11* and *PeMYB12* in V3 since V3 flowers are completely white.

For *Phalaenopsis* F894 (with pink color of flowers), within a three-year period (from 2017-09-01 to 2020-03-31), 9 independent VIGS experiments were performed for *OAGL6-2*, and a total 27 plants with the same mutant phenotype were obtained and analyzed. For *OAGL6-1*, a total 9 independent VIGS experiments were performed, and a total 29 plants with the same mutant phenotype were obtained and analyzed. For *OAP3-1*, a total 6 independent VIGS experiments were performed, and a total 18 plants with the same mutant phenotype were obtained and analyzed. For *OAP3-2*, a total 5 independent VIGS experiments were performed, and a total 14 plants with the same mutant phenotype were obtained and analyzed. For *OPI*, a total 3 independent VIGS experiments were performed, and a total 8 plants with the same mutant phenotype were obtained and analyzed. For *PeMYB11*, a total 5 independent VIGS experiments were performed, and a total 14 plants with the same mutant phenotype were obtained and analyzed. For *PeMYB12*, a total 6 independent VIGS experiments were performed, and a total 18 plants with the same mutant phenotype were obtained and analyzed.

We also tested several VIGS experiments (from 2019-09-03 to 2020-01-21) for *Phalaenopsis* KA38501 with orange-yellow color of flowers. There were 3 independent VIGS experiments each for *OAGL6-2* and *OAGL6-1*, and a total 8 and 12 plants each with the mutant phenotype were obtained and analyzed. There were 2 independent VIGS experiments each for *OAP3-1*, *OAP3-2* and *OPI*, and a total of 8, 4 and 7 plants each with the mutant phenotype were obtained and analyzed. There were 3 independent VIGS experiments each for *PeMYB11* and *PeMYB12*, and a total 6 and 11 plants each with the mutant phenotype were obtained and analyzed.

In summary, for *OAGL6-2*, 27 independent VIGS experiments were performed, and 101 plants with the mutant phenotype were obtained; for *OAGL6-1*, 27 independent VIGS experiments were performed, and 89 plants with the mutant phenotype were obtained; for *OAP3-1*, 14 independent VIGS experiments were performed, and 44 plants with the mutant phenotype were obtained; for *OAP3-2*, 13 independent VIGS experiments were performed, and 36 plants with the mutant

phenotype were obtained; for *OPI*, 9 independent VIGS experiments were performed, and 29 plants with the mutant phenotype were obtained; for *PeMYB11*, 8 independent VIGS experiments were performed, and 20 plants with the mutant phenotype were obtained; for *PeMYB12*, 9 independent VIGS experiments were performed, and 29 plants with the mutant phenotype were obtained.

(5) Based on the data described above, we were not only duplicated numerous independent VIGS experiments but also generated many VIGS plants for each target gene tested. Furthermore, we also performed VIGS experiments for each target gene by using not only one but three different varieties of *Phalaenopsis* orchids, and similar mutant phenotypes were observed. Thus, we have confidence that the phenotype we observed for each target gene is likely reflecting their function. To make this point clearly, three new Supplementary Tables 4-6, describing the total number of VIGS experiments and the plants analyzed for each gene in three different varieties of *Phalaenopsis* orchids, have been provided in the Methods section of the revised manuscript.

(6) As indicated by the reviewer, to make it clear that VIGS silencing effects in this study might be specific for the targeted genes, a paragraph in the Methods section was rewritten to describe how the DNA fragments containing gene-specific sequences for the targeted genes were chosen to ensue the VIGS silencing effects are specific for the targeted genes in the revised manuscript (p.17, lines 4-15 in the Methods section). For this purpose, three new Extended Data Figures 14, 15 and 16, describing the detailed positions and sequences specifically used for target genes, have been provided in the revised manuscript.

3. Given its notorious reputation, in my opinion using VIGS as the only tool for gene functional analysis is only acceptable in 1) plant species that cannot be transformed (thus excluding the generation of stable transgenics), AND 2) if the experiments have been properly conducted: that is, targeting at least 2 independent non-conserved regions (e.g. 5'UTR and 3'UTR) in separate experiments and having the same outcome 3) based on sufficient numbers of plants resulting in statistically sound results. 4) If possible, backed up with functional assays in transformable species. Unfortunately, none of the above criteria have been fulfilled in this manuscript.

Ans: (1) Regarding the question about one VIGS construct per gene only. Please see answers (2) and (3) for question #2.

(2) Regarding the question about only three plants for each VIGS experiment. Please see answers (4) and (5) for question #2.

(3) A large number of VIGS-plants for the same target gene in three different varieties of *Phalaenopsis* orchids (see the answer for question #2) all showed the same altered

phenotype, indicating that the specific gene silencing for each target gene has been achieved and that off-target effects had not likely occurred in our study.

4. Note that in the last couple of years several protocols have been published for stable Agrobacterium based transformation of orchids (including Phalaenopsis orchids used in this study), therefore the authors might consider generating stable CRISPR-Cas9 alleles instead of relying on VIGS for functional analysis. This would strongly enhance the scientific impact of their future studies in orchids. For the current study, the authors might find it too time-consuming to generate stable alleles, but should at least redo the VIGS experiments using separately 5' and 3' UTR regions of the targeted genes, and on a higher number of plants.

Ans: (1) As indicated by the reviewer, protocols have been published for the stable Agrobacterium-based transformation of orchids. However, most reported cases were ectopic expression of a gene and not the knockdown or knockout the genes. Thus, the generation of stable CRISPR-Cas9 alleles is not only time-consuming but also still difficult to generate the silencing or knockout mutations. We hope the reviewer can accept our explanation and agree that the generation of stable CRISPR-Cas9 alleles for our target genes is not essential for the current analysis of the genes in this study.

(2) As the answer to questions #2 and 3, to test the effects caused by using different independent nonconserved regions for a target gene, two different regions (one in K/C domain, one in C domain) for *OAGL6-2* have been used to construct two VIGS constructs (*OAGL6-2*-VIGS-K/C and *OAGL6-2*-VIGS-C) for *OAGL6-2*, and similar big lips phenotypes (lips converted into sepal/petal) were observed in both *OAGL6-2*-VIGS-plants. We thus thought that using one construct with the sequences in the highly variable C-terminal region for *Phalaenopsis* MADS box genes or with the sequences in the variable region located downstream from the highly conserved R2R3 MYB DNA-binding domain for *PeMYB11* and *PeMYB12* genes should be sufficient to obtain the silencing phenotype for these target genes. Therefore, in subsequent VIGS experiments, one DNA fragment specific for a target gene was chosen for *Phalaenopsis* genes in this study. Four new Extended Data Figures 13-16 regarding the answers to this question were provided in the revised manuscript. We hope the reviewer can accept our explanation regarding this issue.

(3) As the answer to questions #2 and 3, a large number of VIGS-plants for the same target gene in three different varieties of *Phalaenopsis* orchids all showed the same altered phenotype, indicating that the specific gene silencing for each target gene has been achieved and that off-target effects had not likely occurred in our study.

5. For the case of petal senescence, the authors have sought to generate additional

proof by an expression analysis of B-class genes in Arabidopsis WT and the etr1 mutant, but this only a further correlation. I do not understand why the authors have not analyzed petal senescence in 35S:AP3 or 35S:PI lines, which should have delayed petal senescence if the claim of the authors would be correct, and would have been a more convincing experiment than the one presented in the manuscript.

Ans: The main theme for this study is to identify the unexpected and additional functions for B and AGL6 proteins that determine lip and sepal/petal identities in orchids, and we found that the orchid B genes *OAP3-1/OPI* have functions outside of floral organ identity specification by regulating perianth senescence. Determining whether there is any correlation for *AP3/PI* orthologues in other plant species that might also possibly be involved in the regulation of this process is interesting but should be considered as supporting data. Thus, we only tested the expression of *Arabidopsis AP3/PI* during different stages of flower development to find the correlation but did not perform any additional experiments in *Arabidopsis.* We did find that there is a positive correlation for the possibility that *Arabidopsis AP3/PI* might have this similar function. Thus, we can only state that "A similar downregulation of *AP3/PI* expression during petal/tepal development/senescence... revealing the possibly conserved role of *AP3/PI* orthologs in regulating flower senescence in flowering plants..." in the Discussion (p.11, line 22 to p.12, line 2). To avoid an unclear or overly strong statement, we also rewrote the inferences about *AP3/PI* genes in the Discussion by adding "likely" in the sentence as "AP3/PI proteins also likely evolved to have a conserved function in Arabidopsis, lily and orchid in regulating petal (Arabidopsis), tepal (lily) and sepal/petal (orchid) senescence" (p.13, lines 2-4). We also deleted the previous Extended Data Figure 12 regarding the evolution of B (*AP3/PI*) and *AGL6* genes in plants in the revised manuscript.

6. Similarly, for identification of protein complexes, usually independent techniques are required as additional proof. In this study, everything is based on one technique (FRET).

Ans: (1) In this study, FRET analysis for orchid MADS-box proteins was performed in *N. benthamiana* leaf cells as described in our previous publication (Hsu et al., Nature Plants, 1, 15046, 2015). Thus, this method could possibly be used alone to interpret the possible formation of any MADS-quartets in tobacco leaf live cells. Since the main theme for this study is to identify the unexpected and additional functions for B and AGL6 proteins that determine lip and sepal/petal identities in orchids, but not the study of the formation of MADS-quartets, to avoid any unnecessary confusion, we thus softened the language we used to interpret the result. For example, the word "likely" was added in the sentence regarding the identification

of the protein complexes throughout the revised manuscript. For example, in the Abstract, the sentence was changed to "Here, we showed that the functional L' (OAP3-1/OAGL6-2/OPI) and SP' (OAP3-2/OAGL6-1/OPI) complexes likely exist by FRET analysis in tobacco leaf cells..." (p.1, lines 15-17). The word "likely" was also added throughout the revised manuscript (p.3, lines 17, 18, 21).

Answer to Reviewer #2:

1. The authors show that these MADS-box complexes may form quartets using FRET analysis in Nicotiana benthamiana leaves (Extended Data Fig. 3), they also show through VIGS that individual genes have distinct functions in flower development. From this they infer that (a) the same quartets that they detected in Nicotiana form in orchids, and (b) that it is differentiation between these quartets that is critical in the variation in gene function they observe in their VIGS analysis. These inferences are never made clear. Instead, the authors say things like, 'Here we show the existence of functional L' and SP' complexes in orchid and to find that AP3/PI/AGL6 genes have evolved to acquire novel functions during evolution' (sentence from the abstract). They do not show the existence of any MADS-quartets in orchids. The language around this point should be softened, and the inferences the authors are making should be made clear throughout the manuscript. The whole abstract needs to be re-written to more accurately reflect the authors' findings.
Ans : (1) In this study, FRET analysis for orchid MADS-box proteins was performed in *N. benthamiana* leaf cells. We apologize for not addressing this clearly in the previous manuscript. We chose tobacco leaf cells for testing to decrease the difficulty of adjusting the protein expression level and to avoid interference from endogenous floral organ MADS-box proteins. As suggested by the reviewer, we added "tobacco leaf cells" in the rewritten sentences in the revised manuscript in the Abstract (p.1, line 17), Results (p.3, lines 12-15) and Methods (p.16, line 19) to clarify the system we used.
(2) As indicated by the reviewer, we did not directly show the existence of any MADS-quartets in orchids. However, since the main theme for this study is to identify the unexpected and additional functions for B and AGL6 proteins that determine lip and sepal/petal identities in orchids, but not the study of the formation of MADS-quartets, to avoid any unnecessary confusion, we thus softened the language we used to interpret the result. For example, the word "likely" was added in the sentence regarding the identification of the protein complexes throughout the revised manuscript. For example, in the Abstract, the sentence was changed to "Here, we

showed that the functional L' (OAP3-1/OAGL6-2/OPI) and SP' (OAP3-2/OAGL6-1/OPI) complexes likely exist by FRET analysis in tobacco leaf cells…" (p.1, lines 15-17). The word "likely" was also added throughout the revised manuscript (p.3, lines 17, 18, 21).

(3) Furthermore, we provided the protein name along with the presumed complex name (p.1, lines, 15-16, 20-21, p.2, lines 1-2) to more accurately reflect the finding of the function for the proteins. Thus, some sentences in the Abstract were rewritten accordingly.

2. Similarly, the authors cannot say much about evolution. There is no outgroup in their analyses or discussion. They are assuming that because what they have observed in their VIGS experiments has not been reported for eudicots (but see below), that the orchid gene function must be novel. Without outgroups, the authors have no way of knowing whether the 'novel' functions they have observed are derived or ancestral. Statements about evolution like the one highlighted above, and in the discussion should be clarified or removed.

Ans: As indicated by the reviewer, the inferences about the evolution of orchid B and *AGL6* genes in the previous manuscript are uncertain and questionable. Since the main theme for this study is to identify the unexpected and additional functions for B and AGL6 proteins that determine lip and sepal/petal identities in orchids, but not the study of the evolution of the B and AGL6 protein functions, to avoid any unnecessary confusion, we rewrote the inferences regarding the evolution of the B and *AGL6* genes in the Discussion and deleted the original Extended Data Figure 12 regarding the evolution of the B *(AP3/PI)* and *AGL6* genes in plants in the revised manuscript.

3. A function for an E-class gene in abscission has been described – the j2 mutant of tomato (Soyk et al, 2017, Cell 169:1142–1155). This paper has not been cited, and should definitely be discussed in this manuscript.

Ans 3: As indicated by the reviewer, a paragraph describing the tomato E-class genes *J2/SEP4* (Soyk et al, 2017, Cell 169:1142–1155) and *SLMBP21* (Liu et al, 2014, Plant J 77:284–296) in pedicel abscission and the comparison for the possible mechanism regulated by orchid *OAGL6-1* and tomato *J2/SEP4* and *SLMBP21* in controlling the abscission of the pedicel was added to the Discussion section (p.12, lines 15 to 22) in the revised manuscript. In addition, these two papers were also cited in the revised manuscript.

4. One aspect of this paper that I particularly liked was the functions for floral MADS-box genes outside of floral organ identity specification. Apart from the

abscission mentioned above, there is a role for PI-like genes in suppressing fruit development in apple (Yao et al, 2001, PNAS 98: 1306–1311). There may be other examples, at least this Yao et al paper should be included/discussed in this manuscript.

Ans 4: As suggested by the reviewer, the reference (Yao et al, 2001, PNAS 98: 1306–1311) was added in the revised manuscript, and a paragraph describing this reference regarding the functions of B genes outside of floral organ identity specification was added to the Discussion section (p.12, lines 2-7).

5. In the figures, the authors label AGL6 ‘OL6’ or ‘L6’, but call the genes in the flower images. The figures would be easier to interpret if the labeling was kept standard – and all were labeled as ‘AGL6’in the figures, text, and figure legends. This is particularly confusing because the authors refer to L and L’ quartets, distinct to the L6 genes.

Ans 5: As suggested by the reviewer, the gene names for *OAGL6-1* and *OAGL6-2* were consistently labeled and kept standard as their full names “OAGL6-1” and “OAGL6-2” in the figures, text, and figure legends throughout the revised manuscript. The full names for “OAP3-1”, “OAP3-2” and “OPI” were also labeled in the figures, text, and figure legends in the revised manuscript.

Answer to Reviewer #3:

1. Figure 1q, Ds and Ls are not capitalized like in the text and legend, change to DS and LS, or does the lower case have meaning?

Ans: As suggested by the reviewer, the "Ds" and "Ls" were changed to "DS" and "LS" throughout the Figures and legends in the revised manuscript.

2. Figure 2, while in Figure 1, the VIGS experiments are indicated by “VIGS” at the end of the name (e.g. Figure 1l, “F894-OL6-2-VIGS”), which helps the reader evaluate the data, in Figure 2, the headings do not include “-VIGS”, which is confusing at first after Figure 1. These figures are data rich, to say the least, small improvements to help the reader evaluate the data is helpful. In addition, the “V” label was then again used in Figure 3, which helps. My suggestion is to use the same symbol, the simplest, “V”, to indicate VIGS plants in all Figures. To give an example how this can be confusing, for Figure 2k, at first glance, it is not clear these are VIGS plants, but indeed they are. In addition, the “M” in Figure k-1 is not defined in the legend, I suppose “M” stands for mock?

Ans: Thank you for reviewer's suggestion. We used a simple “V” to indicate the VIGS plants in all Figures. In addition, we also labeled the full names for the genes tested and the name for the control "Mock" in all Figures to help the reader evaluate the data. For example, OAGL6-1-V, OAGL6-2-V, OAP3-1-V, OAP3-2-V, OPI-V, PeMYB11-V and PeMYB12-V have been used to label the VIGS plants in all Figures in the revised manuscript.

3. Figure 4, while the abscission zone (AZ) cells clearly look different between the control and the L6-1-V (Figure 4j and k), typically the AZ is observed best by longitudinal sections. In addition, it is not clear at what position (in expanded data file, it is stated “slices were cut from stalk base) in the pedicel the samples were taken for the micrographs and histology shown in Figure 4 j-k, g and h. The claim that the AZ is not developed in the L6-1-V may be true, but the anatomical data shown in the manuscript do not clearly show this. To be sure, longitudinal sections that show clearly the AZ in wildtype should be compared with those of the L6-1-V plants. The rest of the data is very convincing, i.e. lower BOP1/2 expression and increase in lignification, but it would be better to show clearly the lack of AZ in these VIGS plants by longitudinal sections. An increase in lignin as shown in Figure 4g and h could take place in the AZ without the abolishment of the AZ entirely. Without this extra data, I do not agree that it can be concluded that the AZ has not developed, or has been abolished as concluded.
Ans: (1) As indicated by the reviewer, we only showed the cross view for the pedicel, which involved staining by phloroglucinol, in the original Figure 4 j-k, g and h. In these figures, the dark red color for lignin was clearly observed in the pedicels of *OAGL6-1*-VIGS *Phalaenopsis* V3; however, the AZ structure in the pedicel was not shown. In this revised manuscript, sections of longitudinal views of the junction of the pedicel and the connected stem stained by phloroglucinol were provided for both wild-type control and *OAGL6-1*-VIGS *Phalaenopsis* V3 (new Figures 4g-h and o-p). The results not only showed that the dark red color for lignin staining was clearly observed in *OAGL6-1*-VIGS *Phalaenopsis* V3 pedicel but also that the red color for lignin extended throughout the pedicel and penetrated into the stem of the *OAGL6-1*-VIGS *Phalaenopsis* V3. In contrast, no dark red color for lignin staining was observed throughout the pedicel until the junction of the stem in the control flower. The strong lignin formation in the *OAGL6-1*-VIGS *Phalaenopsis* V3 pedicel was further confirmed by the strong yellow color staining by Auramine O in the longitudinal views (new Extended Data Figure 8a-b). These new Figures also showed that small differentiated cells formed in the AZ of the control flower pedicel (new Figure 4g, o and new Extended Data Figure 8c, e) were also observed in the junction

of the pedicel and stem in *OAGL6-1*-VIGS pedicel (new Figure 4h, p and new Extended Data Figure 8d, f); however, their formation was clearly altered in the *OAGL6-1*-VIGS pedicel by the penetration of lignin throughout the pedicel to the stem (new Figure 4p and new Extended Data Figure 8d, f). These results revealed that the defect in abscission of the *OAGL6-1*-VIGS pedicel was likely due to the strong increase in lignin formation in the pedicel, which tightly connected the pedicel to the stem and subsequently prevented the abscission of the pedicel from the stem. During this process, AZ was likely formed, although its further development and function were altered in the *OAGL6-1*-VIGS pedicel. Thus, upon manual pedicel removal from the senescent flower, broken cells at the fracture plane and a flattened cavity were observed in the *OAGL6-1*-VIGS pedicel AZs (Figure 4m). Based on these additional results, the Results section for "OAGL6-1 regulates the abscission of pedicels" was rewritten in the revised manuscript (p. 9, line 8 to p.11, line 2).
(2) In addition, a description of the position of the pedicel samples taken for the micrographs and histology was provided in the Methods section as "The junctions for the *Phalaenopsis* flower pedicel and stem were longitudinally cut, whereas 2-mm-thick *Phalaenopsis* pedicle slices were crossly cut from the base of the pedicel using a blade and put into 24-well plates" (p.15, lines 4-6 in the Methods section). In addition, a section for th**e** "Calcofluor White and Auramine O staining" was also provided in the Methods section (p.15, lines 10-18 in the Methods section) in the revised manuscript.

4. How do the authors conclude that there is an unknown “X” protein for senescence and unknown “Y” protein for pedicel abscission? I am not clear on how that can be concluded. I am also unclear how it can be concluded that there are complexes involved in both these processes? Couldn’t this data be explained without the formation of a complex?
Ans: Plant MADS-box proteins may function as higher-order tetrameric complexes based on the floral quartet model. We thus proposed that OAP3-1/OPI may regulate flower senescence in a tetrameric complex with unknown proteins X, whereas OAGL6-1 may regulate pedicel abscission in a tetrameric complex with unknown proteins Y. However, since in planta evidence for MADS-box tetramers remains scarce, we agree that it is not necessary to conclude the formation of the tetrameric complex for OAP3-1/OPI with X and OAGL6-1 with Y, as we used in the previous manuscript. We thus softened the language we used by deleting expressions such as "OAP3-1/OPI could form complex with unknown proteins X.” or "OAGL6-1 could form complex with unknown proteins Y.” from the text, figures and legends in the revised manuscript. However, we thought that it may help the reader to understand the

function of OAP3-1/OPI in regulating flower senescence and OAGL6-1 in regulating pedicel abscission by rewriting the sentences "(OAP3-1+OPI) may form an independent senescence-associated complex (S complex) in suppressing sepal/petal senescence" (p.7, line 20) and "OAGL6-1 proteins may form an abscission complex (A) to promote the abscission of pedicels" (p.22, lines 6-7) in the revised manuscript. Accordingly, the symbols for senescence complex (S) and abscission complex (A) were retained in the figures, whereas the unknown proteins X and Y were deleted.

Reviewer #1 (Remarks to the Author):

My main concern during previous review round was the lack of independent proof for the experiments and main conclusions of the manuscript. In particular, the authors have performed their functional studies using VIGS, but did not provide any additional controls to demonstrate that the observed phenotypes are indeed caused by silencing of the genes under investigation. This is a major point, as VIGS is well known for causing off-target silencing (well documented in the literature). The reason for that is that the dsRNA generated during viral replication, after recognition through Dicer-like riconucleases, will be processed into 21-24 nucleotide fragments (siRNA). Then, based on sequence homology, both plant mRNA and siRNA associate with an RNA-induced silencing complex and will be degraded. Therefore, very short stretches of homology between otherwise non-homologous genes potentially can result in off-target effects. The only way to be 100% sure of the result is therefore to do for every newly targeted gene at least 2 independent VIGS experiments based on the silencing of two non-conserved and non-overlapping regions in the gene. In such a way each construct will lead to two non-overlapping sets of 21-24bp siRNAs. More recently, MIR VIGS (Virus-induced gene silencing using artificial microRNAs) has also been developed in order to reduce the risk of off-target gene silencing (e.g. https://pubmed.ncbi.nlm.nih.gov/23386298/).

In the previous review round, I specifically requested to perform independent VIGS experiments for all the genes studied. See “For the current study, the authors might find it too time-consuming to generate stable alleles, but should at least redo the VIGS experiments using separately 5’ and 3’ UTR regions of the targeted genes, and on a higher number of plants”.

Please find below my further comments on the author’s replies:

(author’s reply) Regarding the question about one VIGS construct per gene only, before the VIGS experiments performed in this study, we did test the effects caused by using different independent nonconserved regions for a target gene. For example, two different regions (one in K/C domain, one in C domain) for OAGL6-2 have been used to construct two VIGS constructs (OAGL6-2-VIGS-K/C and OAGL6-2-VIGS-C) for OAGL6-2 and the phenotypes of the VIGS-plants analyzed. The result showed that a similar big lips phenotype (lips converted into sepal/petal) was observed in both kinds of OAGL6-2-VIGS-plants. One new Extended Data Figure 13 describing these results was provided in the revised manuscript. Thus, we thought that using one OAGL6-2-VIGS construct (with the sequences in the highly variable C-terminal region) should be sufficient to obtain the silencing phenotype for the OAGL6-2 gene.This assumption should also be true for other target genes as long as the DNA fragments chosen contained highly gene-specific sequences for the targeted genes to ensure that the VIGS silencing effects were specific for the targeted genes. Therefore, in subsequent VIGS experiments in this study, just one DNA fragment specific for a target gene was chosen for a Phalaenopsis genes VIGS experiment.

Comment: Because this gives information on the reproducibility of the OAGL6-2 silencing phenotype only, this never can lead to the assumption or conclusion that all other VIGS constructs targeting the other genes in the study must be specific too (since each new construct leads to a new unique set of siRNAs that need to be tested for possible off-target effects). I sincerely regret that the authors have not added proof for the other genes as well, as I otherwise find the results very interesting and beautifully documented.

(author’s reply) Since the VIGS technique is based on the knockdown of a target gene expression and sequentially created a knockdown (not knockout) mutant for the gene, we think each independent VIGS experiment for a target gene could be thought to represent an independent event for generating a similar silencing mutant phenotype with a different degree of silencing for a gene.

Comment: This is true for the targeted gene, but equally true for potential off-targets, so this just shows phenotypic reproducibility of the VIGS construct. Note that unintended off-target effects are reproducible too!

(author's reply) In orchid, many reports for functional analysis of various genes using the VIGS technique have been published during the past years, including the one we published in Nature Plants (Hsu et al., Nature Plants 1, 15046, 2015) regarding the finding of a conserved principle (Perianth code) for orchid perianth formation. Thus, this technique has become very useful and has been considered and accepted as a method to study orchid gene function.

Comment: Not every editor or reviewer is familiar with VIGS, and in general it takes time before the pitfalls of certain techniques become widely known. A typical example is the RNAi silencing technique, for which in the beginning also no additional proof was asked for.

(author's reply) A large number of VIGS-plants for the same target gene in three different varieties of Phalaenopsis orchids (see the answer for question #2) all showed the same altered phenotype, indicating that the specific gene silencing for each target gene has been achieved and that off-target effects had not likely occurred in our study.

Comment: Since there is very high overall conservation of the coding sequence between these varieties, it means that besides the intended silencing target, possible off-target homology is likely also to be conserved between these varieties. Again, this demonstrates reproducibility of the phenotype caused by the VIGS construct, but not that there is no off-target effect.

Reviewer #2 (Remarks to the Author):

This revised version of the manuscript is much improved. My own comments and suggestions were dealt with, and I appreciated the extra detail on the VIGS methods. This paper presents interesting results about MADS-box complex formation and MADS-box gene function in Phalaenopsis, and I think is a good contribution to the field of floral development.

Reviewer #3 (Remarks to the Author):

The authors have addressed my questions and revised the manuscript according to my comments and suggestions. I have no further comment and I am satisfied.

Answer to Reviewer #1:

In the previous review round, I specifically requested to perform independent VIGS experiments for all the genes studied. See "For the current study, the authors might find it too time-consuming to generate stable alleles, but should at least redo the VIGS experiments using separately 5' and 3' UTR regions of the targeted genes, and on a higher number of plants".

Please find below my further comments on the author's replies:

1. (author's reply) Regarding the question about one VIGS construct per gene only, before the VIGS experiments performed in this study, we did test the effects caused by using different independent nonconserved regions for a target gene. For example, two different regions (one in K/C domain, one in C domain) for OAGL6-2 have been used to construct two VIGS constructs (OAGL6-2-VIGS-K/C and OAGL6-2-VIGS-C) for OAGL6-2 and the phenotypes of the VIGS-plants analyzed. The result showed that a similar big lips phenotype (lips converted into sepal/petal) was observed in both kinds of OAGL6-2-VIGS-plants. One new Extended Data Figure 13 describing these results was provided in the revised manuscript. Thus, we thought that using one OAGL6-2-VIGS construct (with the sequences in the highly variable C-terminal region) should be sufficient to obtain the silencing phenotype for the OAGL6-2 gene. This assumption should also be true for other target genes as long as the DNA fragments chosen contained highly gene-specific sequences for the targeted genes to ensure that the VIGS silencing effects were specific for the targeted genes. Therefore, in subsequent VIGS experiments in this study, just one DNA fragment specific for a target gene was chosen for a Phalaenopsis genes VIGS experiment.
Comment: Because this gives information on the reproducibility of the OAGL6-2 silencing phenotype only, this never can lead to the assumption or conclusion that all other VIGS constructs targeting the other genes in the study must be specific too (since each new construct leads to a new unique set of siRNAs that need to be tested for possible off-target effects). I sincerely regret that the authors have not added proof for the other genes as well, as I otherwise find the results very interesting and beautifully documented.
Ans: As suggested by the reviewer, in addition to *OAGL6-2*, we performed additional VIGS experiments in the revised manuscript by using the second independent DNA fragment for all other genes to further demonstrate the lacking of off-target for all the genes and results obtained as described below.

1. For other four MADS box genes (*OAGL6-1*, *OAP3-1*, *OAP3-2* and *OPI*), two different regions (one in the K domain, and one in the C domain) were used to construct two VIGS constructs for *OAGL6-1* (*OAGL6-1*-VIGS-K and *OAGL6-1*-VIGS-C), *OAP3-1* (*OAP3-1*-VIGS-K and *OAP3-1*-VIGS-C), *OAP3-2* (*OAP3-2*-VIGS-K and *OAP3-2*-VIGS-C) and *OPI* (*OPI*-VIGS-K and *OPI*-VIGS-C), and similar altered phenotypes were observed in both kinds of VIGS-plants for each gene as described below.

(1) Similar altered phenotypes such as decreased pigmentations in petals/sepals, increased dark color and lignin formation phenotypes in pedicels and enhanced curvature of the edge in lateral sepals, were observed in *OAGL6-1*-VIGS-K and *OAGL6-1*-VIGS-C flowers. A new Supplementary Figure 14 described this result was provided in the revised manuscript.

(2) Similar altered phenotypes such as decreased pigmentations in sepals and early senescence of sepals were observed in *OAP3-1*-VIGS-K and *OAP3-1*-VIGS-C flowers. A new Supplementary Figure 15 described this result was provided in the revised manuscript.

(3) Similar altered phenotype such as decreased pigmentations in petals was observed in *OAP3-2*-VIGS-K and *OAP3-2*-VIGS-C flowers. A new Supplementary Figure 16 described this result was provided in the revised manuscript.

(4) Similar altered phenotypes such as decreased pigmentations in petals/sepals and early senescence of sepals were observed in *OPI*-VIGS-K and *OPI*-VIGS-C flowers. A new Supplementary Figure 17 described this result was provided in the revised manuscript.

2. For two MYB genes (*PeMYB12* and *PeMYB11*), two different regions with the sequences in the variable region located downstream from the highly conserved R2R3 MYB DNA-binding domain for *PeMYB11* and *PeMYB12* were used to construct two VIGS constructs for *PeMYB12* (*PeMYB12*-VIGS-1 and *PeMYB12*-VIGS-2) and *PeMYB11* (*PeMYB11*-VIGS-1 and *PeMYB11*-VIGS-2), and similar altered phenotypes were observed in both kinds of VIGS-plants for each gene as described below.

(1) Similar altered phenotypes such as decreased pigmentations in petals/sepals were observed in *PeMYB12*-VIGS-1 and *PeMYB12*-VIGS-2 flowers. A new Supplementary Figure 18 described this result was provided in the revised manuscript.

(2) Similar altered phenotypes such as reduced red spot formation in lateral sepal and lobes of lips were observed in both *PeMYB11*-VIGS-1 and *PeMYB11*-VIGS-2 flowers. A new Supplementary Figure 19 described this result was provided in the revised manuscript.

3. Based on the results obtained that the same altered phenotypes were observed for each gene by using the two independent gene-specific target sequences in VIGS experiment, we believe that the specific gene silencing for each target gene has been achieved and that off-target concern had not likely occurred in our VIGS experiments. We sincerely appreciated the carefulness for the reviewer 1 to ask us to further verify our data by performing these additional experiments.

2. (author's reply) Since the VIGS technique is based on the knockdown of a target gene expression and sequentially created a knockdown (not knockout) mutant for the gene, we think each independent VIGS experiment for a target gene could be thought to represent an independent event for generating a similar silencing mutant phenotype with a different degree of silencing for a gene.
Comment: This is true for the targeted gene, but equally true for potential off-targets, so this just shows phenotypic reproducibility of the VIGS construct. Note that unintended off-target effects are reproducible too!
Ans: We agree the reviewer's comment and additional VIGS experiments by using the second independent DNA fragment for all other genes to further demonstrate the lacking of off-target for all the genes were performed in the revised manuscript as described in the answer for comment #1.

3. (author's reply) In orchid, many reports for functional analysis of various genes using the VIGS technique have been published during the past years, including the one we published in Nature Plants (Hsu et al., Nature Plants 1, 15046, 2015) regarding the finding of a conserved principle (Perianth code) for orchid perianth formation. Thus, this technique has become very useful and has been considered and accepted as a method to study orchid gene function.
Comment: Not every editor or reviewer is familiar with VIGS, and in general it takes time before the pitfalls of certain techniques become widely known. A typical example is the RNAi silencing technique, for which in the beginning also no additional proof was asked for.
Ans: We agree and appreciate the reviewer's comment.

4. (author's reply) A large number of VIGS-plants for the same target gene in three different varieties of Phalaenopsis orchids (see the answer for question #2) all showed the same altered phenotype, indicating that the specific gene silencing for each target gene has been achieved and that off-target effects had not likely occurred in our study.
Comment: Since there is very high overall conservation of the coding sequence

between these varieties, it means that besides the intended silencing target, possible off-target homology is likely also to be conserved between these varieties. Again, this demonstrates reproducibility of the phenotype caused by the VIGS construct, but not that there is no off-target effect.

Ans: We agree the reviewer's comment and additional VIGS experiments by using the second independent DNA fragment for all other genes to further demonstrate the lacking of off-target for all the genes were performed in the revised manuscript as described in the answer for comment #1.

Reviewer #1 (Remarks to the Author):

Dear authors,

I appreciate the extra efforts done to convince me (and potential readers) of the reproducibility of the VIGS results. Doing this systematically also for future experiments will certainly enhance the impact of your work.

Answer to Reviewer #1:

Dear authors,

I appreciate the extra efforts done to convince me (and potential readers) of the reproducibility of the VIGS results. Doing this systematically also for future experiments will certainly enhance the impact of your work.

Ans: We sincerely appreciate the reviewer's affirmation of our work and agree the reviewer's comment.
